# Block‐Copolymer‐Architected Materials in Electrochemical Energy Storage

**DOI:** 10.1002/smsc.202300074

**Published:** 2023-11-14

**Authors:** Jörg G. Werner, Yuanzhi Li, Ulrich Wiesner

**Affiliations:** ^1^ Department of Mechanical Engineering Boston University 110 Cummington Mall Boston MA 02215 USA; ^2^ Division of Materials Science and Engineering Boston University 15 St. Mary's Street Boston MA 02215 USA; ^3^ Department of Materials Science and Engineering Cornell University 330 Bard Hall Ithaca NY 14850 USA

**Keywords:** bottom-up fabrication, hierarchical electrodes, ordered nanostructured electrodes, self-assembly, structure direction

## Abstract

The multiscale architecture of electrochemical energy storage (EES) materials critically impacts device performance, including energy, power, and durability. The pore space of nano‐ to macrostructured electrodes determines mass transport within the electrolyte and defines the effective energy density. The dimensions of the active charge‐storing materials can increase stability during cycling by accommodating strains from electrochemical–mechanical coupling while also defining surface area that increases capacitive charge storage, decreases charge‐transfer resistance, but also leads to low efficiency and degradation from interfacial reactions. Thus, elucidating and developing a fundamental understanding of these correlations requires materials with precisely tunable nanoscale architectures. Herein, approaches that take advantage of the nanoscale control offered by block copolymer (BCP) self‐assembly are reviewed and insights gained from associated nanoscale phenomena observed in EES are highlighted. Systematic studies that use custom‐tailored BCPs to reveal fundamental nanostructure–property–performance relationships are emphasized. Importantly, most reports of nanostructured materials utilize low loadings and thin electrodes and results represent mass transfer limitations at the particle scale. However, as cell‐level performance involves mass transport over 10–100s of micrometers, recently emerging BCP‐based processes are further highlighted, leading to hierarchical meso/macroporous materials needed for creating multiscale structure–performance relationships and next‐generation energy storage material architectures.

## Graphical abstract

Synthetic approaches for energy storage materials with controlled nanostructures utilizing block copolymer self‐assembly that elucidate nanostructure–property–performance relationships are reviewed. The impact of mass transfer in electrodes beyond the sub‐micrometer scale to showcase the need for tunable hierarchically porous electrodes is further discussed, and emerging multiscale block copolymer assembly approaches to fabricate such advanced architectures are highlighted.

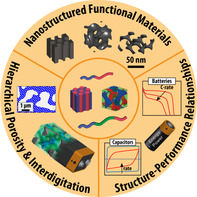

## 1 Introduction

The ability to store electric energy effectively and efficiently enables the sustainable and sustained use of renewable energy generation technologies, as well as the development and advancement of electrification in transportation, mobility, sensing, and other emerging applications.^[^ ^1^ ^]^ For example, renewable energy sources such as solar and wind come with new challenges for the electrical grid, as they cannot be actively and arbitrarily switched on when demand is high, requiring transitory storage solutions such as electrochemical energy storage (EES).^[^ ^2^ ^]^ The important performance metrics for grid storage are cost, scalability, and long‐term cycle stability and rate capability,^[^ ^3^, ^4^ ^]^ while applications with portable energy storage needs such as transportation and electronics require large amounts of energy in small volumes and at low mass, often paired with the need for fast charging capability.^[^ ^4^, ^5^, ^6^ ^]^ Thus, the desired performance metrics for energy storage devices depend on their application space. EES in the form of batteries, electrochemical double‐layer capacitors (EDLCs) and their combinations are attractive solutions due to their excellent performance in at least some of these key parameters. However, for EES to enable widespread electrification of the transportation sector and adoption of renewable energy sources, significant improvements and advances are required, as exemplified by the energy storage targets set by the United States Department of Energy (US DOE), including extreme fast charging (<15 min) with system energy density above 235 Wh kg^−1^ for electric vehicles or the Long Duration Storage Shot for the grid.^[^ ^7^, ^8^ ^]^

Each active part of an EES cell (electrodes, electrolyte, separator) consists of a number of distinct phases to fulfill all the necessary functions, which include electronic and ionic conductivity, ionic accessibility, and structural integrity, as well as redox activity.^[^ ^9^ ^]^ Their performance is defined by both the intrinsic properties and multiscale architecture of these materials: the maximum amount of stored energy is determined by the charge storage capacity of the material as well as its absolute fraction within a cell, while the speed of accessing or storing the energy (power) is dominated by mass transport and pore space architecture, while many degradation mechanisms depend on the characteristic primary dimensions and surface area of the material.^[^ ^10^, ^11^, ^12^ ^]^ Improving these very important characteristics of EES devices often leads to conflicting optimization requirements: for example, increasing charging rate or power capability by introducing higher porosity yields a decrease in energy storage on the device scale and vice versa. This trend is also evident in the major classes of EES devices currently used that fall into the distinct categories of batteries and EDLCs with high energy and power densities, respectively, as illustrated in the Ragone plot in Figure 2a. An understanding of the structure–property–performance relationships is therefore required to deterministically tailor the key performance metrics of EES devices to the various demands for energy storage.

**Figure 1:**
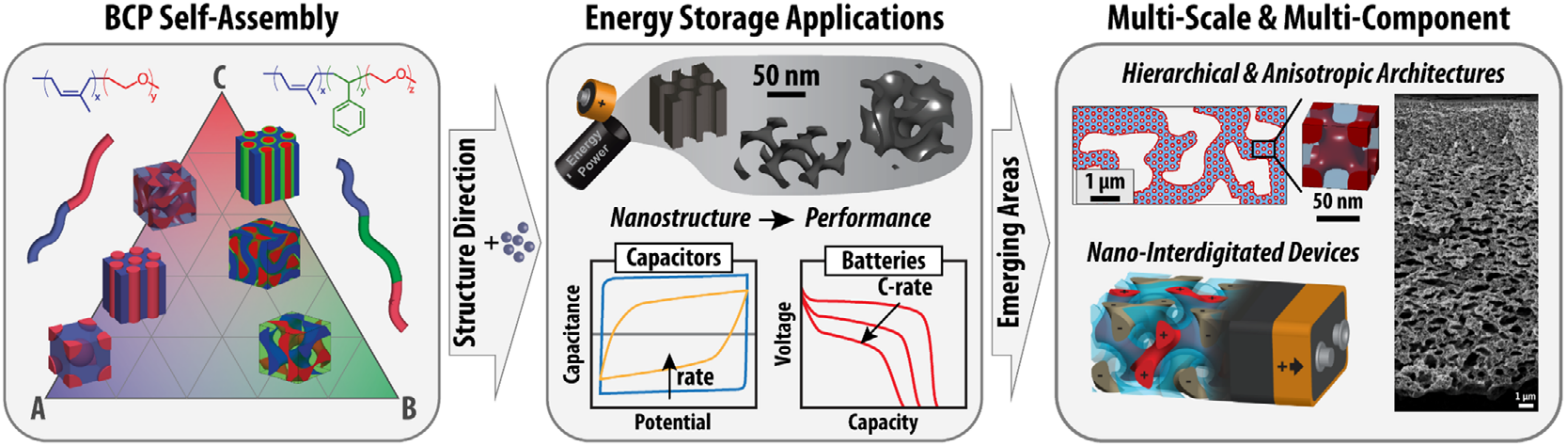
Overview of this review's content: BCP self‐assembly is used to fabricate nanostructured and mesoporous functional materials with tunable parameters to explore structure–performance relationships in supercapacitors, pseudocapacitors, and batteries. The review also provides an outlook into emerging processes and technologies using BPCs to create hierarchically porous and anisotropic/graded material architectures, as well as 3D nanointerdigitated energy storage devices. For right‐most panel: Top left image: Adapted with permission.^[^ ^34^ ^]^ Copyright 2013, American Association for the Advancement of Science. Right image: Adapted with permission.^[^ ^191^ ^]^ Copyright 2015, Royal Society of Chemistry. Bottom left image Adapted with permission.^[^ ^204^ ^]^ Copyright 2018, Royal Society of Chemistry.

In this review following a brief introduction to EES and block copolymer (BCP) self‐assembly, we highlight creative approaches to structure‐direct several classes of EES materials and composites using BCPs (**Figure** **1**). We discuss how custom‐tailored BCPs enable the formation of ordered and homogeneous nanostructures with tunable characteristic pore and material dimensions typically ranging from 3 to 50 nm with a focus on research that utilizes BCP structure direction to systematically study the impact of the (nano)structural parameters on EES performance to derive structure–property relationships. However, it is important to note that most reports of BCP‐architected and other nanostructured EES materials are performed with low material loadings, rendering the reported results representative only for particle‐level mass transfer limitations in thin electrodes that have limited applicability. With the recognition that cell‐level performance also involves and is partially dominated by mass transport over 10–100s of micrometers, hierarchical porosity with a balance between macropores and mesoporosity everywhere is required in future high‐performing EES electrodes, and an application‐tailored balance between the levels and architectures of porosity is required.^[^ ^13^ ^]^ Thus, we also emphasize examples of hierarchical BCP‐derived materials in EES where available, and dedicate the last section to the emerging field of BCP‐architected multiscale structured materials with control over meso‐ and macroporosity and gradation.

### 1.1 EES in Batteries and EDLCs

**Figure 2:**
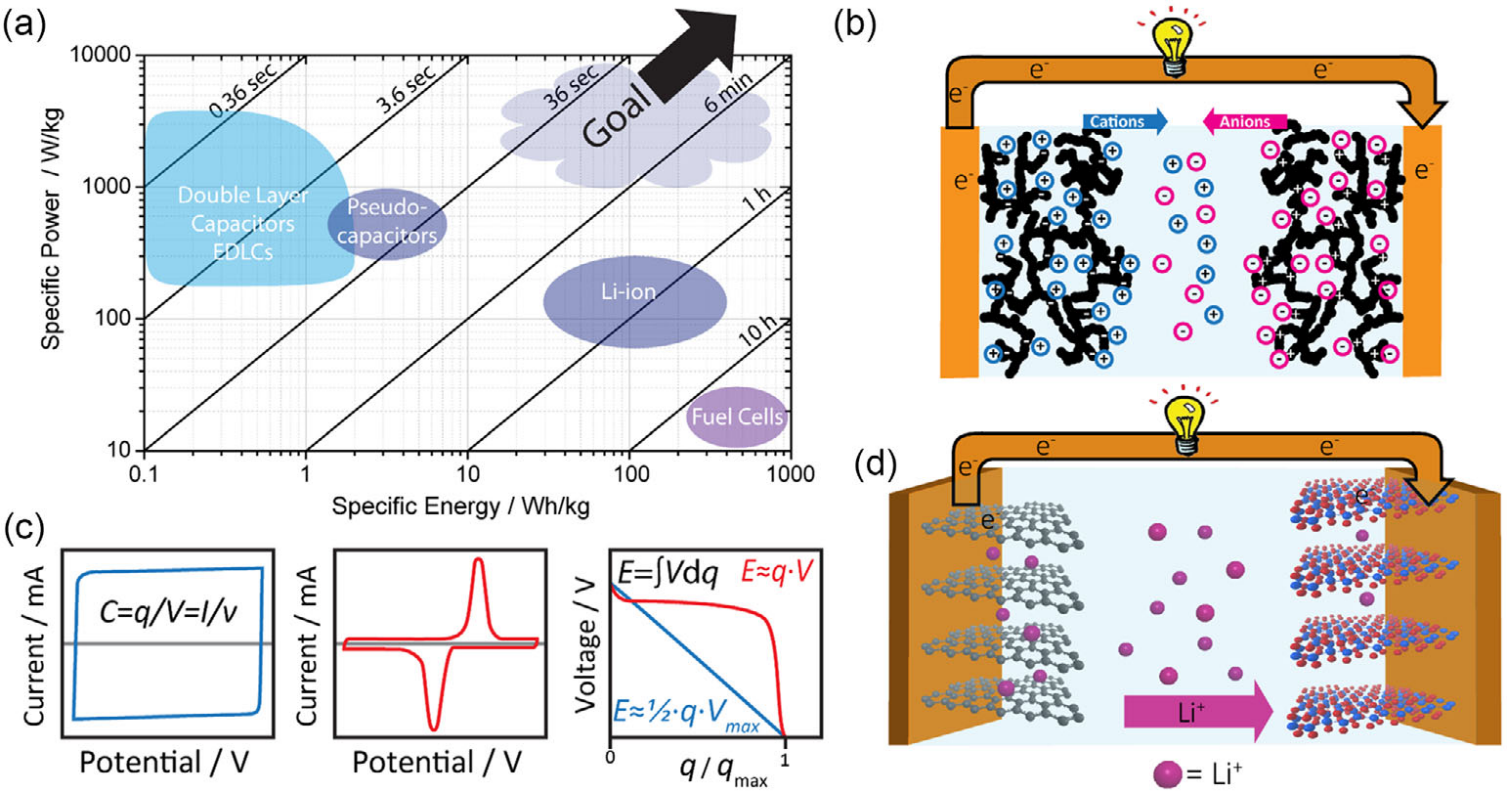
EES: a) specific power density versus energy density with indicated operation ranges of common EES devices. Diagonal black lines represent total (dis)charge times (as indicated) for corresponding specific power–energy pair. b) Schematic representation of the working principle of EDLCs with ionic charge accumulation on the large surface area of a porous electrode immersed in an electrolyte upon application of a voltage. c) Schematic representations of cyclic voltammograms (left and middle) and galvanostatic discharge (right) for double‐layer capacitance (blue) and Faradaic redox electrodes (red) (*C*: capacitance; *q*: stored charge; *V*: voltage; *I*: current; *v*: sweep rate; *E*: energy). d) Schematic representation of the working principle of a LIB with layered anode (graphite) and cathode (lithium cobalt oxide) that (de)intercalate lithium ions as Faradaic charge storage mechanism.^[^ ^15^ ^]^

The two main charge‐storing mechanisms in EES are surface charge accumulation and chemical, Faradaic reactions, which are utilized in EDLCs and batteries, respectively. In EDLCs, energy is stored in the form of charges on the large surface area of a porous electrode immersed in an electrolyte that are counter‐balanced by ionic charges that accumulate in the double layer at the surface, as illustrated in **Figure** **2**b.^[^ ^14^ ^]^ Upon application of a constant current (*I*), for example, the voltage linearly increases and decreases during charge and discharge, respectively, while at a constant voltametric sweep rate (*v*) in cyclic voltammetry (CV), the current is constant, as shown in Figure 2c.^[^ ^15^ ^]^ The capacitance is defined as the amount of charge stored per volt. For high energy density, both the capacitance and the applicable voltage play an important role. High capacitance is reached for conducting electrode materials with high surface areas, often carbonaceous, and liquid electrolytes with high ionic strength and voltage stability, resulting in capacitances of up to 180 F g^−1^ (based on electrode material mass) for pure EDLCs.^[^ ^15^ ^]^ The absence of chemical reactions and ion diffusion in the bulk of solid materials enables very fast rates of charge accumulation/release and, hence, high currents can be applied to charge and discharge capacitors endowing them with high power densities up to 10 kW kg^−1^.^[^ ^16^, ^17^ ^]^ At the same time, the absence of chemical reactions limits the amount of stored charge, and the energy that can be stored in capacitors is typically well below 10 Wh kg^−1^, which is increased in so‐called pseudocapacitive materials that undergo surface‐confined redox reactions, as described in more detail later in this review.^[^ ^7^, ^17^ ^]^

Charge storage via chemical reactions is employed in batteries that involve electron transfer, reduction, and oxidation (“redox”). Chemical redox reactions allow the entire bulk of the active electrode material to be utilized for energy storage, as opposed to just the surface, leading to energy densities of over 250 Wh kg^−1^ in commercial lithium‐ion batteries (LIBs).^[^ ^9^, ^18^ ^]^ The voltage of a battery is determined by the difference in chemical redox potentials of the reactions used in the anode and cathode.^[^ ^18^ ^]^ During charging of a LIB, the cathode material is oxidized while lithium ions are extracted from the cathode into the electrolyte, and simultaneous reduction of the anode happens in concert with lithium uptake from the electrolyte, as illustrated in Figure 2d. Hence, the materials that undergo these redox reactions in batteries determine the amount of charge storage possible per unit mass of active material, as well as the voltage, setting the specific energy (Figure 2c). The high ion‐transfer flux required in batteries, slow diffusion and long diffusion distances, as well as solid‐state chemical reactions that exhibit sluggish kinetics, all result in power densities orders of magnitude smaller than found in EDLCs. Nanostructuring of active materials decreases their solid‐state dimensions and increases their surface area, potentially allowing nanomaterials to bridge the gap between high‐energy batteries and high‐power capacitors.^[^ ^19^ ^]^ However, nanomaterials in EES cause issues of their own, including large amounts of dead volume due to their high porosity leading to poor translation of improvements in materials to device performance, as well as detrimental side reactions at the larger surfaces and interfaces.^[^ ^20^ ^]^

In this review, we describe advances in the use of BCP self‐assembly to control the nano‐architecture of EES materials and components, to develop structure–property relationships, and to eventually narrow the gap between high power and high energy EES devices. Importantly, application‐relevant batteries utilize electrode thicknesses beyond 50 μm, much thicker than typically employed in EES nanomaterials research. The thick electrodes render macroscale mass transport an inevitable contributor to device performance, requiring hierarchically architected electrodes. We highlight dual‐templating approaches to achieve such architectures in the last section of this review.

### 1.2 BCP Self‐Assembly and Structure Direction

The self‐assembly of BCPs is a powerful tool to obtain periodically ordered polymeric morphologies on the nanoscale, as well as to confine the synthesis and assembly of functional organic and inorganic materials into periodic nanostructures. Many chemically distinct polymers are thermodynamically incompatible upon mixing and phase separate on a macroscopic scale like oil and water.^[^ ^21^, ^22^ ^]^ This is due to the low entropy of mixing for polymers that scales inversely with the numbers of monomers in the polymer chain (degree of polymerization, *N*). BCPs contain at least two chemically distinct polymer chains covalently attached to one another, typically at their chain ends, preventing phase separation over macroscopic length scales and leading to microphase separation on the molecular scale (5–100 nm).^[^ ^23^, ^24^ ^]^ Two polymer chains A and B attached to each other in a linear macromolecule are the simplest BCP architecture, termed diblock copolymer. The free energy of mixing, $\Delta F_{m},$ for a two‐component polymer system may be expressed as
(1)

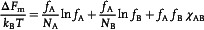

with the Boltzmann constant, *k*_B_, the temperature, *T*, the Flory–Huggins interaction parameter, *χ*_AB_, and the volume fractions, *f*_A/B_, and degree of polymerizations, *N*_A/B_, of blocks A and B.^[^ ^25^ ^]^ While the first two entropic terms are always negative and favor mixing (0 < *f*_A/B_ < 1; *f*_A_ = 1 − *f*_B_), their magnitude decreases for larger polymers with increasing degree of polymerization *N*_A/B_. The Flory–Huggins interaction parameter, *χ*_AB_, is positive for polymers without specific attractive interactions such as hydrogen bonding, charge–charge interactions, or *π–π* interactions, and can be estimated using the interaction energies, *ε*_AA_/*ε*_BB_ and *ε*_AB_, between similar and dissimilar monomers, respectively

(2) $$\chi_{\text{AB}}=\frac{z}{k_{B}T}\left[\varepsilon_{\text{AB}}-1/2\left(\varepsilon_{\text{AA}}+\varepsilon_{\text{BB}}\right)\right]$$

with the number of nearest neighbors per monomer, *z*.^[^ ^26^ ^]^ For most polymer systems, the Flory–Huggins interaction parameter scales approximately inversely with temperature *T*, leading to miscibility at elevated temperature for some polymer mixtures.

**Figure 3:**
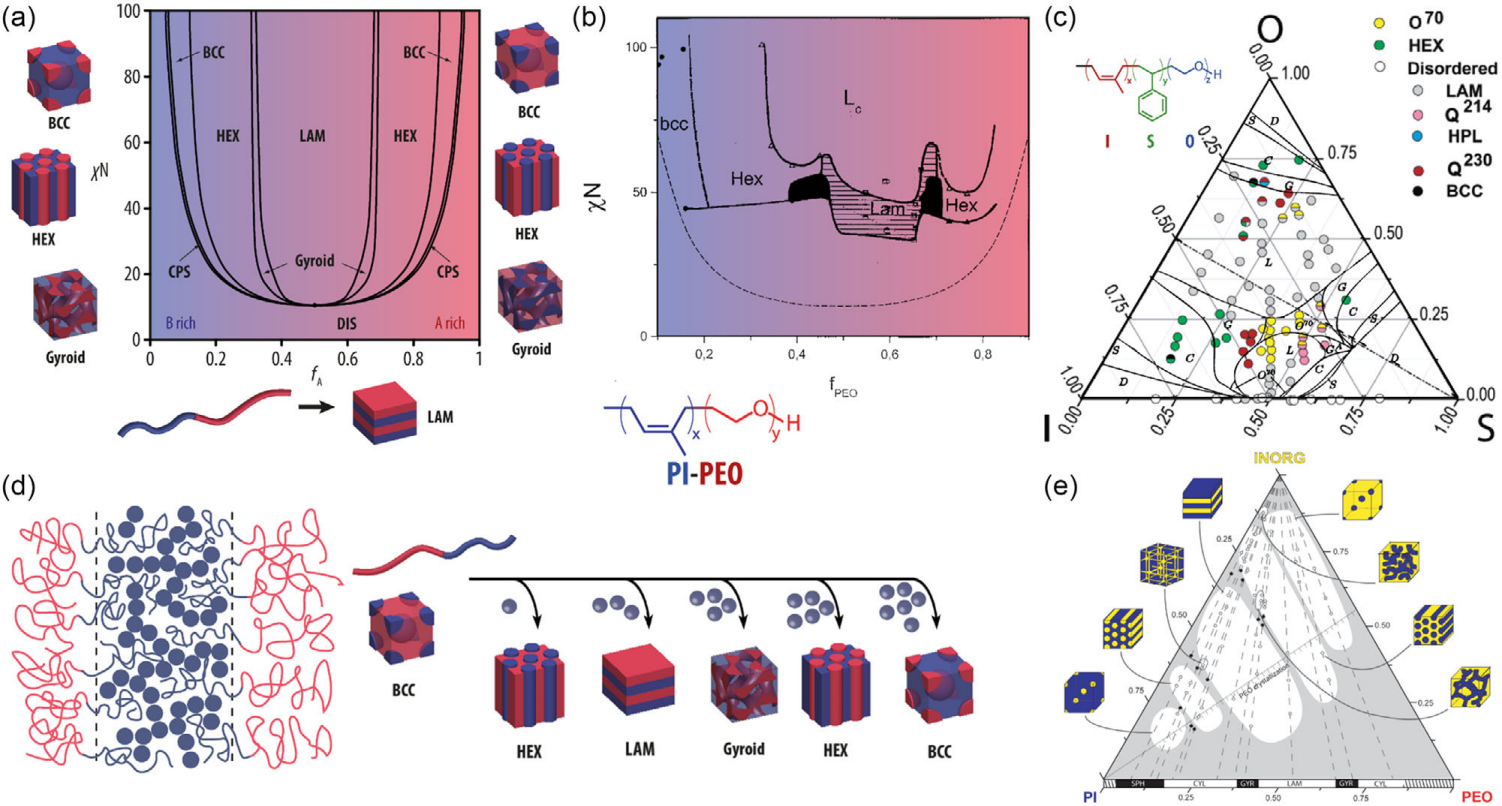
BCP self‐assembly and structure direction. a) Theoretical phase map and equilibrium morphologies of a diblock copolymer. Adapted with permission.^[^ ^27^ ^]^ Copyright 1996, American Chemical Society. b) Experimental phase diagram of the diblock copolymer poly(isoprene)‐*block*‐poly(ethylene oxide) (PI‐*b*‐PEO, or IO). Adapted with permission.^[^ ^29^ ^]^ Copyright 2001, American Chemical Society. c) Overlay of a simulated and an experimental phase map of the triblock terpolymer poly(isoprene)‐*block*‐poly(styrene)‐*block*‐poly(ethylene oxide) (PI‐*b*‐PS‐*b*‐PEO, or ISO). Colored dots are experimentally obtained equilibrium morphologies at the given composition of ISOs with molar mass between 5.8 and 43 kg mol^−1^. Phase boundaries are simulated using statistical segment lengths *b*_I_ = 6.0 Å, *b*_S_ = 5.5 Å, and *b*_O_ = 7.8 Å, and interaction parameters *χ*_IS_N = 11.0, *χ*_SO_N = 14.2, and *χ*_IO_N = 45.8. Adapted with permission.^[^ ^30^, ^31^ ^]^ Copyright 2007, American Chemical Society. d) Schematic illustration of the selective incorporation of small additives into one of the BCP phases (left) and phase tunability with different amounts of selective additives (right).^[^ ^24^ ^]^ e) Ternary phase map of various IO diblock copolymers with organically modified aluminosilicate sol nanoparticles (INORG). (e) Adapted with permission.^[^ ^49^ ^]^ Copyright 2009, American Chemical Society.

Self‐consistent field theory (SCFT) of a symmetric diblock copolymer suggests that microphase separation occurs if *χ*_AB_ *N* is greater than 10.5, with an average degree of polymerization *N*.^[^ ^21^ ^]^ In such symmetric cases, the interface between the two microphase separated blocks containing their covalent junction point is planar. With alternating blocks along the stacking direction, symmetric diblock copolymers assemble in the so‐called lamellar morphology. Thermodynamically stable morphologies for diblock copolymers with volume ratios increasingly dissimilar to 1 are bicontinuous double gyroid (*G* ^D^), hexagonally packed cylinders (Hex), body‐centered cubic (BCC), and closed‐packed cubic (CPC). A simulated phase diagram of a diblock copolymer with respect to *f*_A_ (=1 − *f*_B_) and *χ*_AB_ *N* is depicted in **Figure** **3**a.^[^ ^27^, ^28^ ^]^ The various morphologies with increasing interfacial curvature away from the lamellar arrangement are a result of the balance between the minimization of interfacial energy by reducing their contact area and the reduction of chain stretching, an entropic penalty that occurs when polymer chains deviate from their preferred coiled conformation to decrease the interfacial area at their covalent junction.

Due to the low mobility of polymers and their sluggish kinetics, especially for high glass transition temperature, *T*_g_, polymers, self‐assembly often has to be induced by controlled methods. Experimentally, the self‐assembly of BCPs into their thermodynamically stable morphologies is commonly achieved either thermally or through controlled solvent removal. Thermally stable BCPs can be heated above their glass transition temperature to increase chain mobility and accelerate microphase separation and self‐assembly. However, for polymers with order‐to‐disorder or order‐to‐order transition temperatures (*T*_ODT_, *T*_OOT_) well below their glass transition temperature, as well as thermally unstable polymers, thermodynamically stable morphologies are not obtained upon heating. Hence, in most experimental BCP self‐assembly systems, the slow evaporation of a nonselective solvent from BCP solution is used to induce microphase separation, a process termed evaporation‐induced self‐assembly (EISA). An experimental phase diagram of the diblock copolymer poly(isoprene)‐*block*‐poly(ethylene oxide) (PI‐*b*‐PEO, or IO) is shown in Figure 3b.^[^ ^29^ ^]^ The apparent differences between idealized theoretical and experimental phase diagrams are due to the simplified assumptions made for simulations, such as equal monomer volume and aspect ratio, absence of fluctuations, or noncrystallizable blocks.^[^ ^28^ ^]^ Inclusion of monomer architectures yield simulated phase diagrams that resemble experimental ones more closely, as evident for the linear triblock terpolymer poly(isoprene)‐*block*‐poly(styrene)‐*block*‐poly(ethylene oxide) (PI‐*b*‐PS‐*b*‐PEO, or ISO) shown in Figure 3c.^[^ ^30^, ^31^ ^]^ While numerous architectures such as star, comb, and branched BCPs are possible and their self‐assembly has been studied, linear di‐ and triblock copolymers (AB vs ABA) and triblock terpolymers (ABC) are most commonly employed for structure direction of functional materials.^[^ ^24^ ^]^ **Table** **1** provides an overview of BCPs that are commonly used for structure‐directing functional EES materials.

**Table 1:** Overview of BCPs commonly used for structure directing functional materials

Full name	Abbreviations	Chemical structure	Synthesis methods	References
Poly(ethylene oxide‐*block*‐propylene oxide)‐*block*‐ethylene oxide)	Pluronics, PEO‐*b*‐PPO‐*b*‐PEO	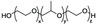	Commercially available	[70]
Poly(isoprene‐*block*‐ethylene oxide)	PI‐*b*‐PEO, IO	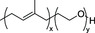	Stepwise carb‐ and oxo‐anionic polymerization	[29]
Poly(isoprene‐*block*‐styrene‐*block*‐ethylene oxide)	PI‐*b*‐PS‐*b*‐PEO, ISO	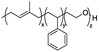	Stepwise carb‐ and oxo‐anionic polymerization	[30]
Poly(styrene‐*block*‐ethylene oxide)	PS‐*b*‐PEO, PEO‐*b*‐PS, SO	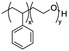	Atom‐transfer radical polymerization (ATRP) onto PEO; stepwise carb‐ and oxo‐anionic polymerization	[33, 34]
Poly(ethylene‐*co*‐butylene‐*block*‐ethylene oxide)	PEB‐*b*‐PEO, KLE	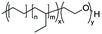	Oxo‐anionic polymerization onto Kraton Liquid	[86]
Poly(styrene‐*block*‐4‐vinyl pyridine)	PS‐*b*‐P4VP, SV	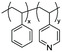	Stepwise carb‐anionic polymerization	[44]
Poly(isoprene‐*block*‐styrene‐*block*‐4‐vinyl pyridine)	PI‐*b*‐PS‐*b*‐P4VP, ISV	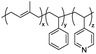	Stepwise carb‐anionic polymerization	[143]
Poly(styrene‐*block*‐acrylic acid)	PS‐*b*‐PAA	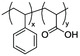	ATRP followed by hydrolysis	[75]
Poly((fluoro)styrene‐*block*‐lactic acid)	P(f)S‐*b*‐PLA	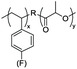	ATRP (PS‐OH) followed by ring‐opening polymerization	[96]
+Poly(n‐butyl acrylate‐*block*‐acrylonitrile)	PBA‐*b*‐PAN	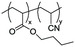	ATRP	[80]
Poly(methyl methacrylate‐*block*‐acrylonitrile)	PMMA‐*b*‐PAN	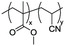	ATRP	[81]
Poly(acrylic acid‐*block*‐acrylonitrile)	PAA‐*b*‐PAN	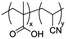	ATRP followed by hydrolysis	[84]

The reproducible and consistent utilization of BCP self‐assembly to fabricate ordered nanostructured EES materials relies on their precise synthesis with regard to molar mass, block fractions, and sequence, as well as macromolecular architecture (e.g., linear vs grafted). Sequential living polymerization techniques are most commonly used to obtain well‐defined and low‐dispersity BCPs, with living anionic polymerization and atom‐transfer radical polymerization (ATRP) at the forefront (Table 1).^[^ ^32^ ^]^ For amphiphilic BCPs comprising hydrophobic and hydrophilic blocks, step‐wise syntheses may be employed using different polymerization reactions. For example, the often‐utilized poly(styrene)‐*block*‐poly(ethylene oxide) (PS‐*b*‐PEO, or SO) is obtained from carb‐anionic polymerization of styrene initiated by *sec*‐butyl lithium followed by cation exchange to potassium and oxo‐anionic polymerization of ethylene oxide, as well as by ATRP of styrene onto a PEO macroinitiator.^[^ ^33^, ^34^ ^]^ Numerous more synthesis strategies have been developed, some of which are sure to find their way into the fabrication of EES nanomaterials, and we refer the interested reader to specialized reviews.^[^ ^35^ ^]^

The selective incorporation of inorganic or organic precursors and nanoparticles into one domain of the self‐assembled BCP morphology enables the precise synthesis of nanostructured functional materials. If the additive can be sufficiently cross‐linked following self‐assembly, dissolution or removal of the BCP yields homogeneous nano‐objects or periodically ordered mesoporous materials depending on the dimensionality and continuity of the initial BCP composite domain.^[^ ^36^, ^37^, ^38^ ^]^ This structure direction using BCPs has expanded the breadth of accessible functional materials with BCP morphologies to amorphous oxides, crystalline oxides, nitrides, metals, carbon materials, semiconductors, and recently even to superconductors.^[^ ^39^, ^40^, ^41^, ^42^, ^43^, ^44^, ^45^, ^46^ ^]^ Key to the successful structure direction, and for obtaining precise functional nanomaterials using EISA, are the use of ultrasmall precursors or nanoparticles, a good common solvent system for all constituents, selective attractive interactions of the functional additive with one of the BCP blocks, and sufficiently high loading of the additive in one of the phases to ensure structural integrity upon removal of the structure‐directing BCP.^[^ ^24^, ^41^, ^47^ ^]^ The first successful incorporation of inorganic materials into larger molar mass BCP bulk morphologies via EISA was demonstrated by Wiesner et al. using PI‐*b*‐PEO and organically modified aluminosilicate sol nanoparticle precursors.^[^ ^48^ ^]^ The selective incorporation of the inorganic sol precursor into the PEO domain of the self‐assembling BCP during EISA not only directed the structure of the sol–gel‐derived inorganic material into a BCP nanostructure, but enabled control over the final morphology through the BCP‐to‐additive ratio, as illustrated in Figure 3d. While the parent BCP self‐assembled into a cubic morphology of PEO spheres packed in a PI matrix, the addition of increasing amounts of inorganic additive yielded hexagonally packed PEO‐additive cylinders and a lamellar morphology. This demonstrated the qualitative analogy between increasing PEO and inorganic swelling additive volume fraction, and paved the way to the tremendous opportunity space BCP structure direction offers. The structural tunability of the system with BCP composition and BCP‐to‐aluminosilicate sol ratio has been summarized in a ternary phase diagram shown in Figure 3e.^[^ ^49^ ^]^ It is worth noting that the addition of hydrophilic inorganic additives not just increases the volume fraction of the hydrophilic polymer block, causing the morphological change, but it also modifies the interaction parameter between the microphase‐separating domains and alleviates polymer stretching and packing frustrations. These effects yield coassembled nanostructures not observed in the parent BCP phase diagram, as is evident in the ternary phase diagram shown in Figure 3e. The length scale of the periodicities of the BCP‐derived nanostructures depends on the molar mass of the BCP and is typically tunable from 5 to 100 nm with pore sizes after thermal processing at higher temperatures in the mesoporous regime (2–50 nm), as illustrated for gyroidal mesoporous carbon materials using the linear triblock terpolymer ISO to structure direct phenol–formaldehyde resols, a common carbon precursor.^[^ ^50^ ^]^

Another common method for the structure direction of functional materials utilizes the solution structures of BCPs. Confined inorganic sol–gel chemistry in the presence of lyotropic liquid crystalline surfactant phases has yielded a variety of ordered mesoporous oxides. Initially accomplished with ionic surfactants, the concept was later successfully extended to nonionic low‐molar mass BCP‐type surfactants.^[^ ^51^, ^52^, ^53^ ^]^ The morphologies obtained from lyotropic liquid crystal templates are qualitatively similar to those of EISA‐induced BCP bulk self‐assembly. Highly uniform pore sizes of the ordered mesoporous materials are typically in the range of 2–10 nm after template removal, owing to the small size of the templating molecules. Due to its simplicity, an alternative solution templating method involves micellar assemblies of BCPs in selective solvents. The solvent evaporation of micelle‐containing solutions with nanomaterials or precursors selectively attracted to the micelle corona yields spherical micelle packings separated by the functional additive.^[^ ^54^ ^]^ While the structures obtained from micelle templating is limited to spherical packings, the pore sizes are highly tunable and homogeneous, depending both on the molar mass of the micelle‐forming BCP and its aggregation number, and hence the solution conditions. The approach recently termed BCP persistent micelle templating (PMT) enables the independent tuning of wall thickness and pore size in ordered mesoporous oxides.^[^ ^55^ ^]^ In PMT, the micelle size is independent of solution conditions and increased additive content leads to thicker walls without changing the mesopore size, an effect that has been used, e.g., to tailor the refractive index in self‐cleaning antireflective optical coatings.^[^ ^56^ ^]^

Over the last two decades, BCP self‐assembly and structure direction has produced a number of concepts and a set of periodically ordered mesoporous materials, which have been successfully applied to EES. For a detailed and thorough summary of synthesis strategies for functional mesoporous materials from BCP self‐assembly, we refer the reader to a recent review.^[^ ^57^ ^]^ Here, we explore and review the structure–function–performance relationship of BCP‐derived nanomaterials and composites in electrodes of batteries, supercapacitors, and hybrids thereof. In particular, studies based on systematic variations of structural parameters in BCP‐directed structures are highlighted that have established fundamental correlations between nanostructure, pore size, and device characteristics and performance. These studies have provided invaluable contributions to the better fundamental understanding of energy storage mechanisms and at the same time have allowed the fabrication of improved batteries and supercapacitors. Much work described in this review is focused on functional materials obtained from BCP self‐assembly at, or close‐to, thermodynamic equilibrium. However, more recently the field of BCP‐derived functional architectures is moving toward nonequilibrium‐based structures and assemblies, e.g., enabling structural asymmetries in parameters like pore size not accessible at equilibrium, likely of importance to improve transport kinetics, as well as multimaterial polyfunctional architected nanohybrids and devices. These developments have already shown a profound impact on EES. Hence, we conclude this review by describing these approaches to provide an outlook on new opportunities, thereby highlighting the emerging but still untapped potential of BCP self‐assembly‐based materials formation for applications in EES.

## 2 BCP‐Derived Supercapacitor Materials

EDLCs store energy through ionic charge accumulation on two purely electronically conducting electrodes that are interfaced with an electrolytic (salt containing) solution.^[^ ^17^, ^58^ ^]^ The voltage that can be applied to an EDLC is limited by the electrochemical stability of the liquid electrolyte. Water‐based electrolytes such as aqueous solutions of potassium hydroxide or sulfuric acid at high molarity are limited to around 1.5 V by the electrolysis potential of water. Using organic electrolytes or ionic liquids, the accessible voltage is increased to over 3 V, doubling the energy density.^[^ ^59^ ^]^ Due to the purely physical surface charge storage mechanism, capacitors can be charged and discharged at very fast rates, yielding high power densities. However, due to the same reason, the amount of charge stored is small compared to mechanisms involving Faradaic reactions, resulting in low energy densities. So far, carbon materials with combined mesoporosity (pore sizes in the range of 2–50 nm) and microporosity (pores sizes < 2 nm) yielding surface areas up to and beyond 2000 m^2^ g^−1^ have resulted the highest gravimetric capacitances (<180 F g^−1^) in traditional EDLCs, limited to a seemingly fundamental BET‐surface‐area‐normalized capacitance of below 15 μF cm^−2^ that decreases with increasing surface area.^[^ ^60^, ^61^ ^]^ Introducing surface‐confined Faradaic reactions increases the charge storage capacity without the severe mass‐transport constraints from solid‐state diffusion that are found in many battery materials. Materials that combine such double layer and Faradaic capacitance are commonly termed pseudocapacitive.^[^ ^17^ ^]^ In this section, we highlight creative BCP‐based synthesis and templating approaches for both traditional EDLC and pseudocapacitive materials, and discuss structure–property relationships with a focus on rate capability that could be obtained thanks to the tunability of the nano‐ and sometimes macroarchitecture of the electrodes. An overview of materials and associated performance data from the research discussed in this section is given in **Table** **2** for carbon‐based materials and in **Table** **3** for transition metal‐based materials.

**Table 2:** Overview of BCP‐derived carbon‐based double‐layer and pseudocapacitive electrode materials

	Material		Porosity				Setup	Performance					References
Composition	Polymer	Morphology	Pore size [nm]	Pore volume <100 nm cm^−3^ g^−1^	Nanoporosity	Surface area [m^2^ g^−1^]	Loading [mg cm^−2^]/thickness [μm]	Low‐rate capacitance [F g^−1^] (<10 mV s^−1^ or <0.5 A g^−1^)	Medium‐rate capacitance [F g^−1^] (≈50 mV s^−1^ or ≈1 A g^−1^)	High‐rate capacitance [F g^−1^] (>1 V s^−1^ or >5 A g^−1^)	Capacitance retention at medium rate	Capacitance retention at high rate	
Carbon	PS‐*b*‐PEO	BCC in nanofibers	28	0.157	24%	635	–	184	155	135	84%	73%	[69]
Carbon	Pluronics + PVP	BCC	3	0.46	48%	659	2/–	170	140	100	82%	59%	[70]
		BCC	3	0.38	43%	575	–	215	200	140	93%	65%	
		HEX	3	0.31	38%	465	–	150	135	105	90%	70%	
N‐doped Carbon (NC)	PBA‐*b*‐PAN	Disordered bicontinuous	13	0.67	57%	500	2–4/–	134/166	80/145	60/120	60%/87%	45%/72%	[80]
NC CO2‐activated			13	1.09	69%	1140	–	151/164	–	–	–	–	
NC KOH‐activated			3/10	2.13	81%	2570	–	173/176	–	–	–	–	
S/N‐doped Carbon	PBA‐*b*‐PAN	Disordered bicontinuous	6	–		506	15/76	125	113	45	90%	36%	[82]
			14	–		488	–	165	128	89	78%	54%	
			18	0.53	51%	478	–	236	155	130	66%	55%	
			22			519	–	125	121	77	97%	62%	
N‐doped Carbon			16	0.56	53%	480	–	158	130	75	82%	47%	
N‐doped Carbon	PMMA‐*b*‐PAN	Disordered bicontinuous	6	0.41	45%	418	4/–	141	130	80	92%	57%	[81]
			10	0.46	48%	389	–	192	170	125	89%	65%	
			12	0.56	53%	350	–	207	180	145	87%	70%	
			12	0.51	50%	318	–	223	200	150	90%	67%	
			14	0.48	49%	289	–	254	235	195	93%	77%	
			15	0.46	48%	237	–	168	150	105	89%	63%	
			18	0.4	44%	213	–	137	110	82	80%	60%	
Carbon	PI‐*b*‐PS‐*b*‐P4VP	Hierarchical graded meso/macropores	20	1.69	77%	1024	0.32/8	–	140 (50 mV s^−1^)	100 (5 V s^−1^)	–	71%	[194]

**Table 3:** Overview of BCP‐derived transition metal‐based double‐layer and pseudocapacitive electrode materials

Material			Porosity				Setup	Performance					References
Composition	Polymer	Morphology	Pore size [nm]	Pore volume <100 nm cm^−3^ g^−1^	Nanoporosity	Surface area [m^2^ g^−1^]	Loading [mg cm^−2^]/thickness [μm]	Low‐rate capacitance [F g^−1^] (<10 mV s^−1^ or <0.5 A g^−1^)	Medium‐rate capacitance [F g^−1^] (≈50 mV s^−1^ or ≈1 A g^−1^)	High‐rate capacitance [F g^−1^] (>1 V s^−1^ or >5 A g^−1^)	Capacitance retention at medium rate	Capacitance retention at high rate	
TiN	PI‐*b*‐PS‐*b*‐P4VP	Hierarchical graded meso/macroporous architecture	41	0.59	81%	90	0.95/60	–	45	25	–	56%	[194]
TiN		Single‐gyroid mesoporous monoliths	24	1.29	90%	139	4.56/65	–	64	17	–	27%	[194]
PAni/MoS_2_	PS‐*b*‐PEO	Mesoporous micellar PAni on 2D MoS_2_	12	–	–	258	2	500	370	220	74%	44%	[85]
			16	–	–	221	–	420	335	175	80%	42%	
	Pluronics	Mesoporous cylindrical PAni on 2D MoS_2_	10	–	–	205	–	270	230	130	85%	48%	
TiO_2_ sol–gel	KLE	Body‐centered cubic	14	–	–	180‐200	–	533 (0.5 mV s^−1^)	267	–	50%	–	[93]
TiO_2_ nanocrystals		Distorted cubic micellar lattice	25	0.32	58%	250–300	0.03/0.15	667 (0.5 mV s^−1^)	400	–	60%	–	
T‐Nb_2_O_5_	KLE	Body‐centered cubic	14	0.09	29%	150	0.05/0.18	370 (2 mV s^−1^)	300	–	81%	–	[86]
Nb_2_O_5_ (amorphous)		Body‐centered cubic	14	0.09	29%		0.05/0.18	233 (2 mV s^−1^)	200	–	86%	–	
CeO_2_	KLE	FCC pore structure	14	0.07	34%	150	0.04/0.09	75 (0.5 mV s^−1^)	45	–	61%	–	[94]
V_2_O_5_	PfS‐*b*‐PLA	Double‐gyroid mesoporous structures	11	0.49	62%	148	0.15/1.15	–	–	155	–	–	[97]

### 2.1 Soft‐Templated Hierarchical Mesoporous Carbon Electrodes

The rate at which EDLCs and pseudocapacitors can be charged and discharged is determined by the diffusion of the ions in the electrolyte as well as the electronic conductivity of the electrodes. In traditional EDCLs with highly porous carbon materials, the resistance to ion diffusion is the dominant factor and architectural features besides high surface area are of importance to obtain high power capabilities.^[^ ^62^ ^]^ The tunability and homogeneity of pore sizes and morphologies from BCP self‐assembly has made it a valuable tool to determine structure–property relationships of electrode materials, especially in evaluating both the impact of the mesoporosity and of the micrometer‐scale architecture on capacitance and its retention (or loss) at high rates. Since the first report on BCP soft‐templated mesoporous carbon by Dai et al. almost 2 decades ago,^[^ ^44^ ^]^ a rich variety of ordered mesoporous carbons (OMCs) with pore sizes spanning from 1.5 to 40 nm have been synthesized and applied to EES and conversion.^[^ ^63^ ^]^ In this section, we highlight research on BCP‐derived carbonaceous materials as EDLC electrodes with a focus on complex and hierarchical architectural features because it is clear that electrodes with application‐relevant footprint capacitance (F cm^−2^) require an understanding and optimization of both the micro/mesopores that provide high surface area as well as larger pores of 100s of nanometers to micrometers that act as ion “highways” and reservoirs for high‐rate applications (vide supra). For an in‐depth summary of general mesoporous carbon materials in supercapacitors, we refer the interested reader to numerous and excellent dedicated reviews.^[^ ^63^, ^64^, ^65^, ^66^ ^]^

**Figure 4:**
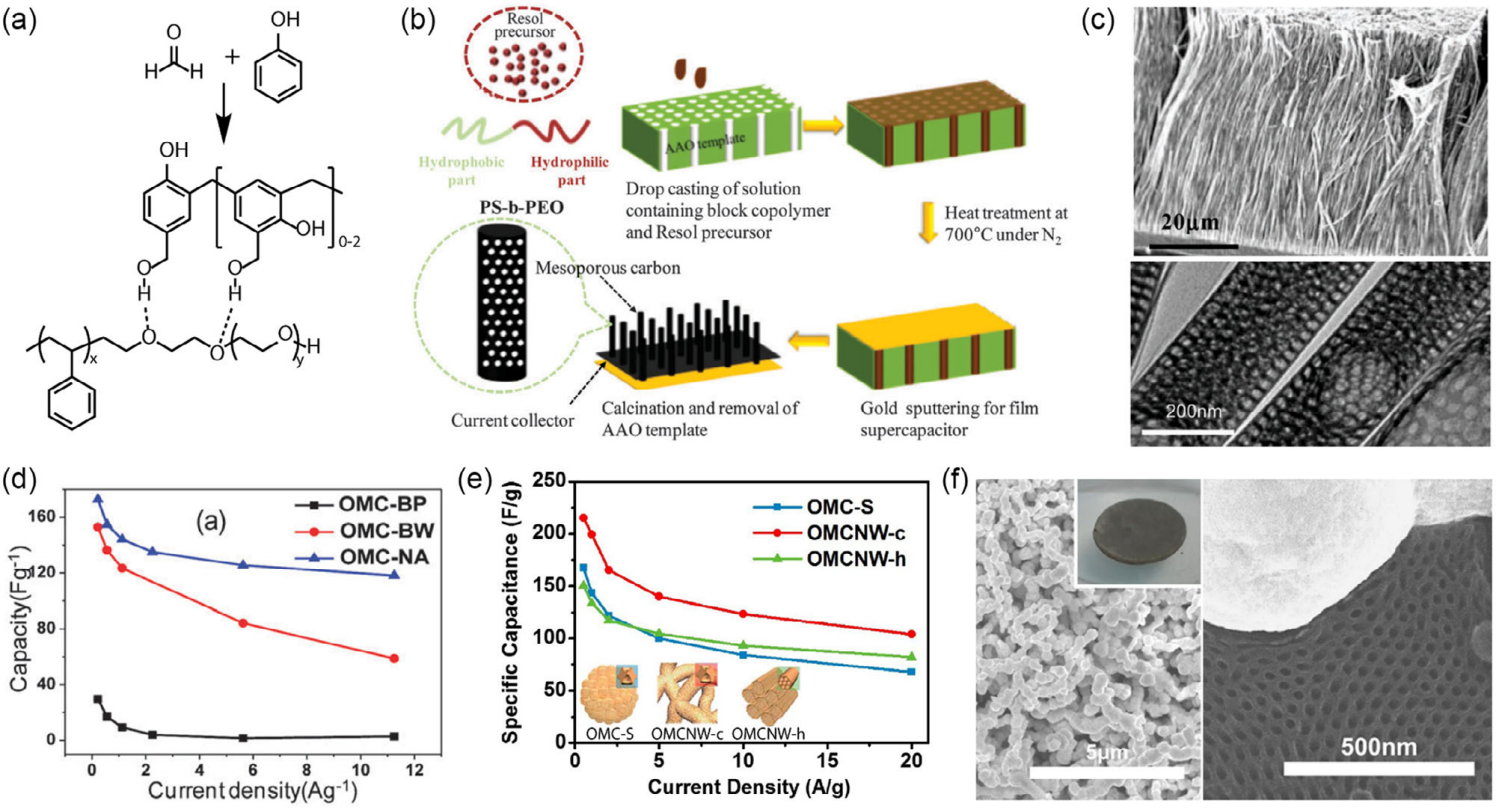
Mesoporous carbons from BCP coassembly with phenol–formaldehyde (PF) resols. a) Chemical structures of species involved in PS‐*b*‐PEO BCP structure direction of PF resols. b) Illustration of the synthesis of densely grafted mesoporous carbon nanofibers through the coassembly of PS‐*b*‐PEO with PF resols inside the pores of an anodized alumina (AAO) membrane. c) Scanning (top) and transmission (bottom) electron microscopy images of the OMC nanofibers (OMC‐NA). d) Capacitance versus current density plots of OMC‐NA, OMC bulk wires (OMC‐BW), and OMC bulk powders (OMC‐PB) when used as supercapacitor electrodes. (a–d) Adapted with permission.^[^ ^69^ ^]^ Copyright 2013, The Royal Society of Chemistry. e) Specific capacitances at various current densities of dual‐soft‐templated hierarchical mesoporous carbon structures obtained from BCP–resols–poly(vinylpyrrollidone). Adapted with permission.^[^ ^70^ ^]^ Copyright 2015, Royal Society of Chemistry. f) Scanning electron microscopy (SEM) images of a nickel nanofoam before (left) and after (right) coating with hexagonally OMC from PS‐*b*‐PEO/resols. Inset shows a photograph of the electrode disk. Adapted with permission.^[^ ^71^ ^]^ Copyright 2016, American Chemical Society.

The first reported OMC has been synthesized using a preassembled thin film of poly(styrene)‐*block*‐poly(4‐vinylpyridine) (PS‐*b*‐P4VP) and resorcinol with hexagonally packed cylindrical morphology and subsequent resorcinol cross‐linking with gaseous formaldehyde.^[^ ^44^ ^]^ Since then, oligomeric resols obtained from phenol and formaldehyde cross‐linked under basic conditions have become the dominant carbon precursor that is coassembled with amphiphilic BCPs to directly achieve (soft‐templating) ordered BCP–resols hybrids of various morphologies (**Figure** **4**a). Moderate heat treatment at 80–150 °C of the self‐assembled materials leads to further cross‐linking of the resols confined in the hydrophilic domains of the BCP, permanently fixing the nanostructure. Pyrolysis at elevated temperatures of 400–450 °C under inert conditions causes the decomposition of the BCP that introduces mesopores in place of the hydrophobic BCP domains, while temperatures above 600 °C lead to carbonization of the phenol–formaldehyde resins. By tuning the BCP composition, molar mass, and BCP‐to‐resols ratio, a variety of morphologies (hexagonally packed cylinders, cubic micellar, 3D gyroidal) and pore sizes (4–40 nm) for OMCs have been achieved.^[^ ^50^, ^67^ ^]^ The all‐organic synthesis of OMC materials also allows for the application of solvent‐annealing to obtain highly ordered gyroidal mesoporous carbon thin films.^[^ ^68^ ^]^

The facile and direct fabrication method of OMCs using the coassembly of amphiphilic BCPs with phenol–formaldehyde resols enables its application to dual‐templating methods to achieve hierarchically porous structures. As ion diffusion to the carbon surface determines the power and rate capability of EDLCs, complex architectures featuring micro‐, meso‐, and macropores can lead to good capacitance retention at high current or voltametric sweep rates. For example, by confining the coassembly of poly(styrene)‐*block*‐poly(ethylene oxide) (PS‐*b*‐PEO) and resols inside anodized alumina membranes, OMC nanofiber arrays were obtained, as illustrated in Figure 4b.^[^ ^69^ ^]^ The nanofibers exhibited diameters of around 250 nm stemming from the membrane template pore size, and 28 nm ordered mesopores from the self‐assembly of the custom‐made BCP with a molar mass of 26 kg mol^−1^. Due to the dual‐templating method, the nanofiber array is densely grafted vertically on a metal current collector without the need of conductive additives or binders (Figure 4c). At a low current density of 0.23 A g^−1^, the OMC nanofiber array exhibited a capacitance of 173 F g^−1^, a common value for mesoporous carbon materials. However, the hierarchical architecture of the OMC nanofiber array electrodes enabled the retention of 68% of the low‐rate capacitance at a high current rate of 11.25 A g^−1^. The advantage stemming from the complex architecture is evident when compared to the inferior rate capability of bulk OMC fibers and powder obtained from the same BCP–resols system (Figure 4d).^[^ ^69^ ^]^

The importance of structural and pore connectivity of mesoporous carbon electrodes in EDLC applications has been demonstrated by 0D and 3D OMCs. Using a dual soft‐templating route of a self‐assembling Pluronic–resols system with poly(vinyl pyrrolidone) (PVP), OMC spheres and OMC nanonetworks were obtained with 4 nm mesopores from the Pluronics self‐assembly and additional 60 nm macropores in the dual‐templated nanonetworks.^[^ ^70^ ^]^ The hierarchical architecture of the OMC nanonetworks allowed for a capacitance retention of over 50% upon increase of the current density from 0.5 to 20 A g^−1^, in contrast to the 0D OMC spheres with less than 40% capacitance retention (Figure 4e). Coating OMC thin films onto a 3D nickel nanofoam allows for intimate contact between a high surface area carbon material and the metal current collector (Figure 4f).^[^ ^71^ ^]^ The coassembly of PS‐*b*‐PEO with resols inside the macroporous nickel foam yielded hexagonally packed cylindrical pores of the carbon coating with a diameter of 38 nm and increased the capacitance of the nickel nanofoam by two orders of magnitude, even at a low carbon‐to‐nickel weight ratio of 0.01. The nickel–OMC composite showed an impressive 82% capacitance retention upon increasing the current rate from 1 to 200 A g^−1^, while the absolute gravimetric capacitance was rather low due to the high content and weight of nickel. Nevertheless, the work demonstrates the advantage of 3D integrated current collectors for ultrahigh‐power applications in thick (high loading per footprint) electrodes.

### 2.2 BCP‐Derived Pseudocapacitor Materials

Traditionally, batteries and electrochemical capacitors have been regarded as distinct energy storage devices, each having their own advantages, challenges, and application areas.^[^ ^7^ ^]^ In contrast to capacitors, batteries stand out for their high energy density from the use of Faradaic chemical reactions for charge storage, but suffer from sluggish kinetics and power capabilities.^[^ ^9^ ^]^ With the emerging use of renewable, intermittent energy sources and the increasing electrified transportation sector, energy storage devices with high energy and fast charge/discharge capability (power) are needed. Hence, research needs to focus on the discovery and fundamental understanding of novel electrode materials, composites, and architectures that store energy in Faradaic reactions at rates and reversibility that rival capacitive charge storage.

One solution to this challenge are surface‐confined Faradaic redox reactions that increase the amount of charge stored beyond the ionic double layer (EDLC) but without solid‐state diffusion limitations compromising the rate capability.^[^ ^16^, ^72^ ^]^ Such surface‐confined Faradaic reactions include the coupled proton–electron transfer in nitrogen‐containing compounds as well as nondiffusion‐limited intercalation in the nanometric surface layers of various metal oxides.^[^ ^73^, ^74^ ^]^ While the term “capacitance” is strictly speaking incorrect in its use for Faradaic charge transfer, these materials are typically characterized the same way as capacitors due to their additional high double‐layer capacitance. Thus, the term “pseudocapacitance” has been coined for this energy storage mechanism. To distinguish between, and evaluate the contribution of, the different charge storage mechanisms of EDLCs, pseudocapacitance, and bulk Faradaic reactions, CV under various scan rates and electrolytes is typically employed:^[^ ^72^ ^]^ current of capacitive contributions (EDLC and pseudo) scale linearly with sweep rate, while diffusion controlled bulk redox reactions as present in batteries scale with the square root of the sweep rate. Double‐layer current is independently measured using redox‐inactive ions. As with EDLCs, pseudocapacitors require high surface areas and sufficient porosity to facilitate electrolyte access to the redox‐active sites, establishing the need for nanostructured materials. In this section, we highlight creative approaches that utilize BCP self‐assembly to enable pseudocapacitive charge storage materials and identify structure–property performance relationships. It needs to be noted, however, that most of this research so far has focused on low‐loading electrodes (<2 mg cm^−2^) that only allow to probe the intrinsic material capacitance and the rate capability as determined by the mesoporosity and particle level (Table 1 and 2). A transition to application‐relevant electrode and cell dimensions and loading demands the development and understanding of hierarchical electrodes with application‐tailored multiscale porosity. Recently, novel approaches using BCPs to obtain and control such hierarchical architectures with multiscale porosity have emerged. These advances and their potential future impact as next‐generation electrodes are discussed.

#### 2.2.1 Nitrogen‐Containing Mesoporous Carbonaceous Electrodes

Some nitrogen embedded in the graphitic or pyrolytic atomic framework of carbon materials has been shown to increase the capacitance of high surface area electrodes due to the Faradaic charge storage contribution of nitrogen functional groups. While nitrogen functionality can be incorporated in various atomic environments, the most commonly found atomic arrangements in polymer‐derived nitrogen‐doped carbon are pyridinic (pyridine, pyridine oxide, pyridonic, N‐defects in graphitized carbon) as well as pyrrolic. Nitrogen is typically introduced into mesoporous carbon materials from BCP self‐assembly by supplementing the carbon precursor with nitrogen containing molecules such as dopamine, aminophenol, aniline, or melamine– and urea–formaldehyde resins.^[^ ^33^, ^75^, ^76^ ^]^ A recent study further compared the doping of gyroidal mesoporous carbon with nitrogen using pyridine‐containing resols with a solid‐state doping method using ammonia gas at high temperatures.^[^ ^77^ ^]^ The nitrogen content remaining in the mesoporous carbon material after carbonization strongly depends on the pyrolysis temperature. At higher temperature, more nitrogen (and oxygen) functionalities outgas as small molecules and the carbon content increases. Nitrogen contents up to 20 wt% have been observed for nitrogen‐doped mesoporous carbons from urea–formaldehyde resins that were structure‐directed and in situ polymerized with poly(styrene)‐*block*‐poly(acrylic acid) (PS‐*b*‐PAA) after pyrolysis at 600 °C.^[^ ^75^ ^]^ As higher electronic conductivity often requires carbonization at temperatures above 800 °C, typically obtained nitrogen contents in BCP‐derived nitrogen‐doped mesoporous carbons are well below 10 wt%.^[^ ^33^, ^76^ ^]^ However, the increase in capacitance from even a few percent nitrogen doping is significant: mesoporous carbon spheres self‐assembled from PS‐*b*‐PEO and dopamine with less than 3 wt% nitrogen exhibit a capacitance of up to 350 F g^−1^ at low rates of 0.1 A g^−1^, with 50% retention at 10 A g^−1^.^[^ ^33^ ^]^ The mesoporous carbon spheres obtained from PS‐*b*‐PEO BCP with varying PS block size but constant PEO size showed a decreasing linear relationship of both surface area and low‐rate capacitance on the mesopore size, but nonlinear behavior of the rate performance, demonstrating the need for well‐engineered architectures on all length scales to meet the requirements of a given application. In a similar recent study of PS‐*b*‐P4VP‐templated resorcinol‐based mesoporous carbon monoliths with small amounts of nitrogen (2.5 at%), a nonmonotonic relationship between pore size and surface area to specific and surface‐area‐normalized capacitance has been demonstrated.^[^ ^78^ ^]^ Apart from the unusually high capacitance (up to 500 F g^−1^ and 89 μF cm^−2^ based on surface area) for carbon‐based EDLCs, the use of purely nanoporous (<105 nm pore size) monoliths as electrodes led to high volumetric energy density of over 20 Wh L^−1^ at moderate current densities (1 A g^−1^). Importantly, by screening pore sizes from 35 to 105 nm, the study demonstrated the importance of ion diffusion in the electrolyte (pore) space even for supercapacitors and not just for batteries, which require significantly more ions to move across the electrode: while smaller pore sizes showed higher energy (volumetric and gravimetric), the largest pore size enabled the highest power, demonstrating their often inverse relationship and the need for precise multiscale control over pore space architecture and its correlation to performance metrics in energy storage electrodes and devices.

**Figure 5:**
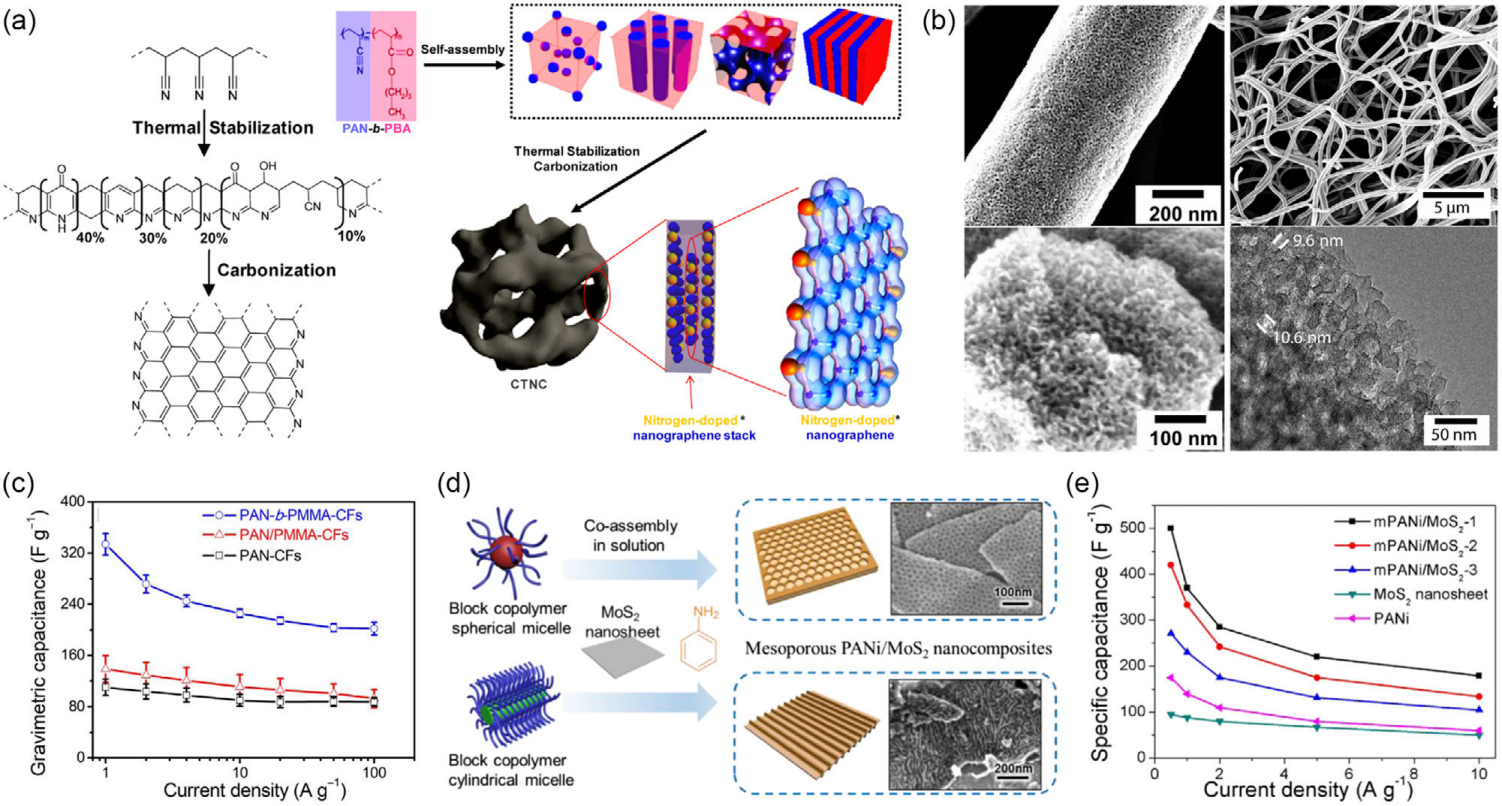
Nitrogen‐containing mesoporous carbonaceous supercapacitor materials. a) Chemical structures and illustration of carbonization steps of poly(acrylonitrile)‐containing BCPs. Adapted with permission.^[^ ^80^ ^]^ Copyright 2012, American Chemical Society. b) Electron microscopy images of mesoporous carbon fibers derived from electrospun PAN‐*b*‐PMMA. c) Capacitance versus current density plots of mesoporous BCP‐derived carbon fibers (PAN‐*b*‐PMMA‐CFs), macroporous polymer blend‐derived carbon fibers (PAN/PMMA‐CFs), and dense carbon fibers (PAN‐CFs). (b,c) Adapted under the terms of the CC‐BY Creative Commons Attribution 4.0 International license (https://creativecommons.org/licenses/by/4.0).^[^ ^83^ ^]^ Copyright 2019, The Authors, published by American Association for the Advancement of Science. d) Illustration (left) and electron microscopy images (right) of MoS_2_ nanosheets coated with BCP‐templated mesoporous aniline (mPANI/MoS_2_). e) Capacitance versus current density plots of various mPANI/MoS_2_ architectures and of pure PANI and MoS_2_ electrodes. (d,e) Adapted with permission.^[^ ^85^ ^]^ Copyright 2017, American Chemical Society.

A direct method to synthesize nitrogen‐doped mesoporous carbon is the pyrolysis of poly(acrylonitrile) (PAN) containing BCPs.^[^ ^79^ ^]^ PAN is the most used precursor for carbon fiber production. But the propensity of PAN to crystallize interferes with the self‐assembly of PAN‐based BCPs into well‐ordered structures, and microphase separation typically leads to lamellar or disordered nanostructures. PAN oxidatively cross‐links upon heat treatment in air (280 °C) forming a ladder‐type molecular structure, which is a necessary stabilization step before pyrolysis in inert atmosphere at higher temperature for carbonization.^[^ ^80^ ^]^ Using a thermally degradable second block such as poly(*n*‐butyl acrylate) (PBA) or poly(methyl methacrylate) (PMMA), mesoporous nitrogen‐doped carbon materials are obtained after EISA, oxidative stabilization, and carbonization without the need of additional carbon precursors, as illustrated in **Figure** **5**a.^[^ ^80^, ^81^, ^82^, ^83^ ^]^ It is generally observed for EDLC electrode materials that a larger specific surface area results in a higher specific capacitance due to the increased number of ions in the electrochemical double layer at the material–electrolyte interface. This trend is often also observed in N‐doped mesoporous carbon with low amount of nitrogen (<5 at%), as demonstrated by Zhong et al. using activation of OMCs to tailor the specific surface area without changing the PBA‐*b*‐PAN‐derived nanostructure. When chemically activated with KOH or thermally activated with CO_2_, the carbons exhibit specific surface areas of 1140 and 2570 m^2^ g^−1^ and specific capacitances of 151 and 173 F g^−1^, respectively. However, if pseudocapacitive charge storage is dominant over double‐layer capacitance, this simple geometric dependence becomes invalid and the presence of and accessibility to Faradaic surface groups are key factors. For example, pore sizes can be easily controlled by the size of the sacrificial block and their influence on capacitance has been demonstrated by using PMMA‐*b*‐PAN with varying PMMA block lengths and constant PAN size, resulting in seven distinct materials with pore sizes from 6 to 18 nm and decreasing surface area with increasing pore size.^[^ ^81^ ^]^ Interestingly, the maximum capacitance and rate capability of 254 F g^−1^ at 0.5 A g^−1^ and 73% retention at 10 A g^−1^, respectively, were obtained for the mesoporous carbon with 14 nm pore size and a moderate surface area of 289 m^2^ g^−1^ using 2 m KOH electrolyte. The nonmonotonic relationship between both pore size and surface area with specific capacitance was speculated to be due to the “most appropriate pore size” at 14 nm, though no physical explanation or hypothesis was provided. As the Debey length and solvation shell of ions are much smaller than any of the studied pore sizes, other factors such as nitrogen content (not determined) or macroscopic structural aspects such as electrode density may play a role as well.

The need for the oxidative stabilization or cross‐linking step in air at >250 °C before carbonization prevents incorporation of other heteroatoms as dopants into PAN‐based BCP‐derived mesoporous carbons. To overcome the challenge of codoping, Yuan et al. recently demonstrated that elemental sulfur plays a dual role to both oxidatively stabilize PAN while simultaneously doping the resulting carbon with up to 8 at% sulfur functionalities in addition to the intrinsic nitrogen dopant.^[^ ^82^ ^]^ In their electrochemical tests with 6 m KOH, the N/S‐codoped mesoporous carbon outperforms the air‐stabilized N‐doped material with 236 F g^−1^ versus 159 F g^−1^ and a high surface‐area‐normalized capacitance of 50 μF cm^−2^ at 0.1 A g^−1^ that decreases to 24 μF cm^−2^ at 10 A g^−1^. In a systematic study, the authors further identified the intermediate pore size of 18 nm to exhibit the highest capacitance and rate capability for a set of N/S‐codoped mesoporous carbons ranging in pore size from 5 to 22 nm while also demonstrating less relative capacitance loss for larger mesopores (Table 2). The origin of this pore‐size‐dependent capacity was not studied but speculated to originate from a balance between pore size and Debey length. However, at the employed electrolyte concentrations, the Debey length is an order of magnitude smaller than the pore size, and other effects such as micropore size distribution and surface roughness may contribute. Such structural effects may also explain the lower gravimetric capacitance compared to other PAN‐based BCP‐derived mesoporous carbons, though the use of a much higher material loading per footprint of electrode (15 versus 4 mg cm^−2^) most likely also plays a significant role especially at high current densities where ion transfer in the electrolyte is limiting.^[^ ^81^ ^]^

By introducing hierarchical architectural features, the rate capability of PAN‐BCP‐derived nitrogen‐doped mesoporous carbon is significantly improved. Electrospinning of polymers and polymer hybrids is a well‐established method to create high‐aspect ratio fibers and fibrous assemblies with diameters of micrometers or even sub‐micrometer length scales. Electrospinning of BCPs typically yields disordered structures due to the fast evaporation rate during the spinning process, but uniform mesoporosity with bicontinuous morphology has recently been obtained from electrospinning PAN‐*b*‐PMMA or blends of PAN with PAN‐poly(acrylic acid) BCPs.^[^ ^83^, ^84^ ^]^ For example, the PAN‐*b*‐PMMA fibers carbonized at 800 °C were around 550 nm in diameter and exhibited connected and homogeneous mesopores of 9–10 nm, as shown in Figure 5b. The mesoporous carbon fiber mats were used as supercapacitor electrodes and their performance compared to dense carbon fibers from PAN homopolymers, as well as macroporous carbon fibers from a homopolymer blend of PAN and PMMA. The BCP‐derived carbon fibers showed impressive capacitance and rate capability of 360 F g^−1^ at 1 A g^−1^ and 202 F g^−1^ at 100 A g^−1^, significantly higher than the dense or macroporous carbon fibers (Figure 5c).^[^ ^83^ ^]^ The authors calculated 37.8% of the measured capacitance to be contributed by pseudocapacitive charge storage involving the nitrogen‐active sites of the carbon. The influence of the architectural features beyond the mesoporous structure indicates again the importance of hierarchical structures for applications requiring fast mass transport or ion diffusion. At the same time, the random webs of mesoporous carbon fibers can yield excessive amounts of porosity between the fibers, leading to low volumetric and gravimetric capacitances on the device scale (including the electrolyte volume and mass). Hence, for practical energy storage applications, a careful balance has to be struck between macroporosity for fast ion accessibility and electrode density for high device‐level energy density. Correlations and optimization for this multiscale parameter space have yet to be established experimentally, likely with the help of simulations.

An intriguing multifunctional hierarchical architecture of mesoporous poly(aniline) (mPANI) thin films coated on molybdenum disulfide nanosheets (mPANI/MoS_2_) has been obtained by micellar BCP templating.^[^ ^85^ ^]^ Spherical or cylindrical micelles of PS‐*b*‐PEO or Pluronics P123 BCPs, respectively, adsorbed to the surface of MoS_2_ nanosheets templated the polymerization of aniline to form a mesoporous PANI thin film, as shown in Figure 5d. The MoS_2_ nanosheets gave rise to high capacitance of over 500 F g^−1^ at 0.5 A g^−1^, while the mesoporous PANI coating provided good electronic and ionic conductivity, leading to a capacitance retention of 179 F g^−1^ at 10 A g^−1^. The significantly higher capacitance of the hierarchical hybrid nanomaterial compared to the sum of its constituents indicated a synergistic effect of the materials and architecture (Figure 5e).

#### 2.2.2 BCP‐Derived Niobia‐Based Pseudocapacitor Materials

**Figure 6:**
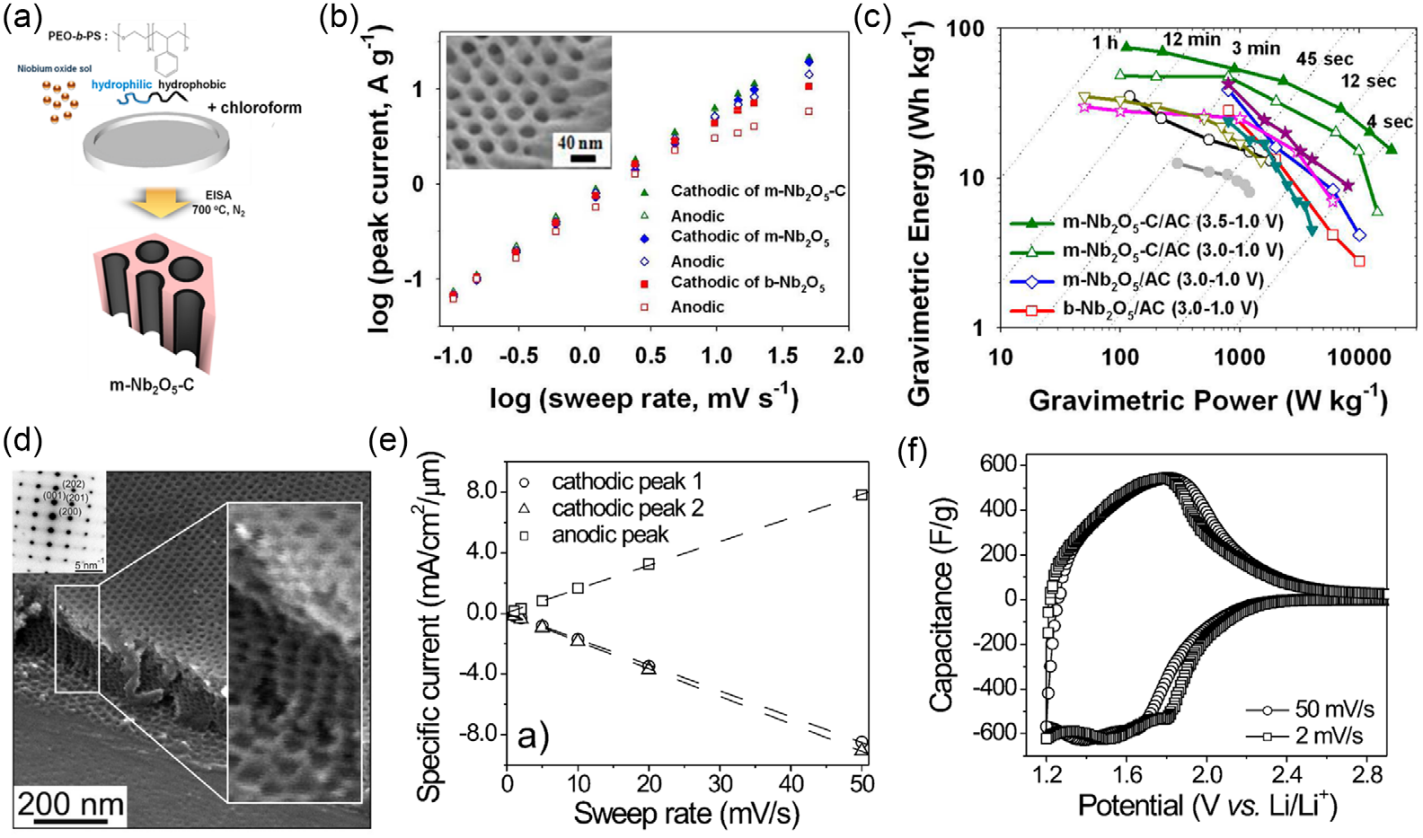
a–c) Hybrid supercapacitor with ordered mesoporous niobia‐carbon (m‐Nb_2_O_5_‐C) pseudocapacitive anode. a) Scheme of EISA of PS‐*b*‐PEO and niobia sol and subsequent pyrolysis under inert atmosphere (CASH method). b) Log–log plot of peak current versus scan rate between 0.1 and 50 mV s^−1^ showing the power law exponent of 1 for m‐Nb_2_O_5_‐C and deviation toward 0.5 for bulk niobia (b‐Nb_2_O_5_) at higher scan rates. Inset shows SEM image of m‐Nb_2_O_5_‐C. c) Ragone plot of different niobia anodes demonstrating the advantage of mesoporosity and carbon hybridization. (a–c) Adapted with permission.^[^ ^90^ ^]^ Copyright 2014, American Chemical Society. d–f) KLE‐micelle‐templated Nb_2_O_5_ thin film pseudocapacitor electrode. d) SEM image of thin film showing the ordered and open mesoporosity. Inset: electron diffraction pattern of film indicating the oriented crystal growth. e) Linear current‐sweep rate dependence of the KLE‐based Nb_2_O_5_ thin film demonstrating the capacitive charge storage behavior. f) Cyclic voltammograms (current normalized by sweep rate to display capacitance) of the KLE‐based Nb_2_O_5_ thin film at 2 and 50 mV s^−1^. (d–f) Adapted with permission.^[^ ^86^ ^]^ Copyright 2010, American Chemical Society.

Initial efforts in pseudocapacitive materials not based on organic nitrogen functionality have focused on ruthenium and manganese dioxides with ultrafast Faradaic surface reactions. Over the last decade, numerous other transition metal oxides have been found to exhibit pseudocapacitive charge storage even beyond surface redox functionalities if appropriately nanostructured. At the forefront is niobium pentoxide (Nb_2_O_5_, “niobia”) that demonstrates intercalation pseudocapacitance, resulting in a high energy density compared to surface‐confined Faradaic reactions while maintaining a reasonable rate capability only an order of magnitude lower than typical EDLCs.^[^ ^74^ ^]^ Intercalation pseudocapacitance occurs when ion intercalation leads to reduction of metal centers beyond the surface of the electrode material with capacitive properties, such as a linear relationship between current and sweep rate in CV. For niobia, intercalation pseudocapacitance is observed for the orthorhombic crystalline phase (T‐Nb_2_O_5_) with crystallite sizes on the nanoscale and at high surface areas.^[^ ^86^, ^87^ ^]^ The synthesis of crystalline mesoporous niobia from BCP self‐assembly has been extensively studied employing hydrolytic and nonhydrolytic sol–gel methods with hexagonally packed cylindrical as well as bicontinuous gyroid morphologies.^[^ ^88^, ^89^, ^90^, ^91^ ^]^ The synthesis of mesoporous crystalline oxides typically follows a process termed combined assembly by soft and hard (CASH) chemistries which enables heat treatment of BCP‐derived nanostructures to temperatures above 1000 °C without structural collapse, yielding fully crystalline ordered mesoporous inorganic materials such as titania and niobia, as illustrated in **Figure** **6**a.^[^ ^40^ ^]^ Such ordered mesoporous niobia–carbon materials were employed as the anode material in hybrid supercapacitors.^[^ ^90^ ^]^ In hybrid supercapacitors (HSC), a redox‐active electrode is paired with an EDLC electrode to increase the energy density without compromising the power capability of the device. Therefore, it is critical to match the rate capability of the redox electrode to the ultrafast charge storage of EDLC materials such as activated carbon. The CASH method enables the synthesis of high surface area orthorhombic niobia with nanosized (e.g., 12 nm) crystallites and high conductivity due to intimate carbon coating. The mesoporous carbon‐coated T‐Nb_2_O_5_ (m‐Nb_2_O_5_‐C) anode and the corresponding HSC exhibited a linear relationship between current and sweep rate up to 5 and 50 mV s^−1^, respectively, demonstrating the large pseudocapacitive contribution of the niobia even at relatively high rates, as shown in Figure 6b.^[^ ^90^ ^]^ The m‐Nb_2_O_5_‐C‐based HSC exhibited excellent power of 18.5 kW kg^−1^ at a high energy density of 15 Wh kg^−1^. However, the capacitance drop of 10% over only 1000 cycles highlighted the prevailing issue of reversibility still to be addressed in pseudocapacitors employing Faradaic processes. A very similar mesoporous carbon‐coated T‐Nb_2_O_5_ was also studied as sodium anode material.^[^ ^92^ ^]^ The importance of crystallinity, porosity, and nanostructure on the intercalation pseudocapacitance of niobia was demonstrated in micelle‐derived niobia thin films employing commercial poly(ethylene‐*co*‐butylene)‐*block*‐poly(ethylene oxide) (PEB‐*b*‐PEO) BCP, also known as KLE, as structure‐directing agents.^[^ ^86^ ^]^ Interestingly, the mesoporous niobia exhibited macroscopic crystallographic orientation despite the small crystallite size with the [0*k*0] axis parallel to the film normal, independent of the substrate used. The iso‐oriented mesoporous T‐Nb_2_O_5_ films with a thickness of around 100 nm exhibited a linear current‐to‐sweep rate relation in CV and no capacitance loss up to 50 mV s^−1^ (Figure 6d–f).

#### 2.2.3 Other BCP‐Derived Transition Metal Oxide‐Based Pseudocapacitor Materials

**Figure 7:**
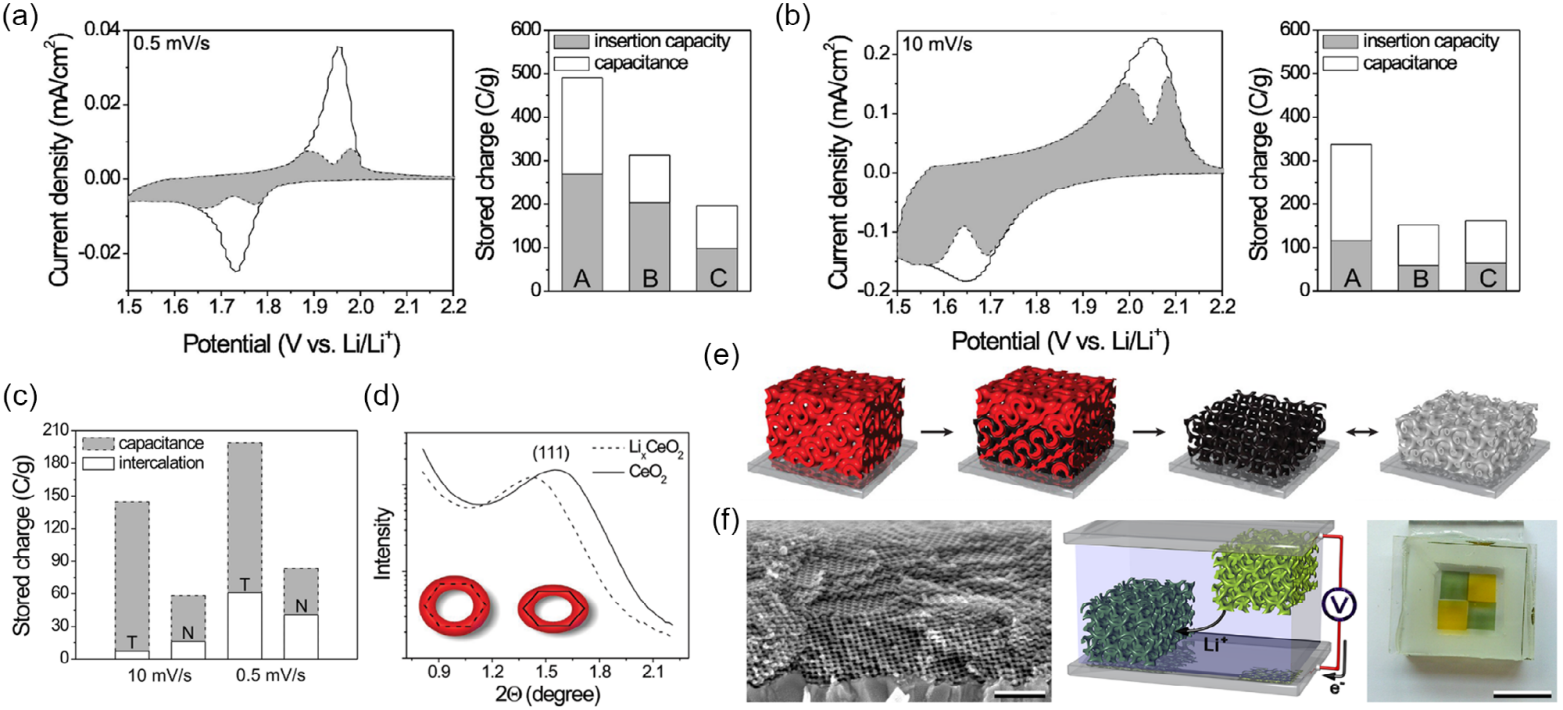
a,b) Cyclic voltamograms (CVs, left) of KLE‐templated titania nanocrystals and derived charge storage contributions (right) of various anatase titania thin film electrodes at (a) 0.5 mV s^−1^ and (b) 10 mV s^−1^: A: KLE‐templated titania nanocrystals; B: KLE‐templated sol–gel‐derived titania; C: Nontemplated titania nanocrystals. (a,b) Adapted with permission.^[^ ^93^ ^]^ Copyright 2009, American Chemical Society. c) CV‐derived charge storage contributions of KLE‐templated (T) and nontemplated (N) CeO_2_ thin film electrodes demonstrating the increased pseudocapacitance due to mesostucturing. d) SAXS patterns of lithiated and nonlithiated KLE‐templated CeO_2_ showing the mechanical deformation during electrochemical cycling. (c,d) Adapted with permission.^[^ ^94^ ^]^ Copyright 2010, American Chemical Society. e) Scheme of metal (oxide) electrodeposition inside a mesoporous double‐gyroid polystyrene (PS) matrix after removal of poly(lactic acid) (PLA) from a gyroidal PS‐*b*‐PLA thin film and subsequent electrochemical conversion leading to changing optical properties. Adapted with permission.^[^ ^96^ ^]^ Copyright 2013, American Chemical Society. f) Cross‐sectional SEM image (left) and schematic (middle) of gyroidal V_2_O_5_ electrochromic thin film pseudocapacitor. Photograph (right) shows the different colors of the electrode patches in the charged state. Adapted with permission.^[^ ^97^ ^]^ Copyright 2012, American Chemical Society.

Transition metal oxides such as titania, ceria, vanadia, manganese, and tungsten oxide also exhibit pseudocapacitance to some degree when fabricated as porous nanocrystalline architectures. BCP‐derived mesoporous metal oxides demonstrate high surface areas and accessibility for surface redox reactions, and additionally provide mechanical stress release from volume expansion that occurs due to Faradaic near‐surface intercalation or conversion reactions. Brezesinski et al. demonstrated this for various early transition metal oxides such as titania, ceria, and the above‐discussed niobia using KLE‐micellar templating of ordered mesoporous thin films.^[^ ^86^, ^93^, ^94^, ^95^ ^]^ Mesoporous anatase titania thin films from both sol–gel precursors and nanocrystals were studied to reveal their pseudocapacitance and charge storage performance. Higher insertion capacity and pseudocapacitance were observed when employing preformed nanocrystals instead of sol–gel‐derived titania, as shown in **Figure** **7**a,b.^[^ ^93^ ^]^ The authors contributed this result to the additional microporosity from the interstitial space between nanocrystals, though exposure of certain crystal faces or crystal orientations could also contribute. The electrochemical properties of KLE‐templated ceria (CeO_2_) further demonstrated the importance of mesostructure and control over the nanoarchitecture. Ceria typically exhibits low capacity and sluggish redox kinetics. However, when mesostructured with BCPs, a large pseudocapacitance can be harnessed due to accessible surface redox sites and mechanical flexibility, as shown in Figure 7c,d.^[^ ^94^ ^]^ Even in the extensively studied lithium‐ion cathode material Li_*x*_Mn_2_O_4_, a large pseudocapacitive charge storage contribution was found in KLE‐templated thin films.^[^ ^95^ ^]^ At sweep rates up to 10 mV s^−1^, both CV redox peaks (3.9 and 4.1 V versus Li/Li^+^) exhibited highly reversible electron transfer with peak separations of less than 59 mV and exponents close to 1 in the current‐sweep rate power law, with some deviation at higher sweep rates.

Transition metals typically exhibit oxidation state‐dependent colors or opaqueness, which can be used to electrically control the transmission of light of certain wavelengths. Steiner et al. studied the simultaneous electrochromic and pseudocapacitive properties of BCP‐derived gyroidal thin films of nickel oxide and vanadium oxide.^[^ ^96^, ^97^ ^]^ Spin‐coated and thermally annealed poly((4‐fluoro)styrene)‐*block*‐poly(d,l‐lactide) thin films were used as templates for electrodeposition of the active materials after poly(lactide) hydrolysis, as illustrated in Figure 7e. Symmetric pseudocapacitor devices employing transparent electrodes coated with patches of gyroidal vanadium oxide exhibited high reversibly capacitance of 155 F g^−1^ at 10 A g^−1^, over an order of magnitude larger than nontemplated vanadium oxide thin films. At the same time, the electrode patches changed color from green (discharged) to green‐blue (reduced) and yellow (oxidized) in their charged state, as shown in Figure 7f.^[^ ^97^ ^]^ Using gyroidal nickel and nickel oxide thin films as the electrodes, fast and reversible switching between black and semitransparent supercapacitors with millisecond response times was achieved.^[^ ^96^ ^]^

#### 2.2.4 BCP‐Derived Hybrid Carbon–Metal Oxide‐Based Pseudocapacitor Materials

Tungsten oxide is an attractive material for EES due to its multiple oxidation states, low cost, and relative abundance. The intrinsic electronic resistance of WO_3_ necessitates the use of conductive additives or partial reduction to WO_3−*x*_. In a comprehensive study, Jo et al. compared a BCP CASH‐derived mesoporous WO_3−*x*_–carbon hybrid material with hard‐templated mesoporous partially reduced mesoporous WO_3−*x*_.^[^ ^98^ ^]^ The 24 wt% carbon equipped the hybrid material with double‐layer capacitance in addition to the redox capacity and pseudocapacitance, leading to overall higher energy and power densities compared to mesoporous reduced WO_3−*x*_ or pure mesoporous carbons. The strategy of adding electronic conductivity to redox‐active metal oxides by hybridizing with small amounts of carbonaceous material can also be inverted by adding redox activity to carbon‐based EDLC electrodes. Hybridizing OMCs with small amounts of redox‐active transition metal oxides has increased the energy density of supercapacitors. The addition of 10–20 wt% cobalt or vanadium precursors to the synthesis of Pluronics‐derived OMCs caused higher electronic conductivity, possibly due to catalytic graphitization, as well as larger capacitance.^[^ ^99^ ^]^ The additional capacitance is attributed to the finely dispersed redox‐active metal centers in the high surface area carbon nanomaterial. Interestingly, attempts to obtain vanadium oxide–carbon hybrids through the CASH method with argon as the inert gas during pyrolysis led to the collapse of the mesostructured materials, potentially due to carbothermal reduction of vanadia, while the same procedure in nitrogen gas preserved the BCP‐derived morphology.^[^ ^100^ ^]^

PAN‐based BCPs that directly convert to mesoporous carbon can be hybridized with small amounts of transition metals or their oxides in a one‐pot method or through infiltration to yield highly dispersed pseudocapacitive nanocrystals on mesoporous carbons.^[^ ^101^, ^102^, ^103^ ^]^ For example, the addition of up to 20% iron oxide nanoparticles (Fe_2_O_3_‐NPs, <3 nm) into a PBA‐*b*‐PAN‐derived OMC increased the capacitance due to the iron oxide redox activity.^[^ ^101^ ^]^ The use of a hydrophobic PBA block and hydroxybenzyl alcohol‐capped Fe_2_O_3_‐NPs enabled the selective incorporation of the nanoparticles into the PAN‐derived carbon phase, while other syntheses schemes employing PEO‐*b*‐PAN with inorganic precursors yielded core–shell carbon–metal oxide composites.^[^ ^104^, ^105^ ^]^ More recently, mesoporous carbon fibers from PAN‐based BCPs loaded with manganese oxide or ruthenium nanocrystals have been reported with ultralarge‐specific capacitances beyond 500 F g^−1^ due to pseudocapacitive effects.^[^ ^102^, ^103^ ^]^ Importantly, the use of fibers enabled high specific electrode loadings (up to 7 mg cm^−2^) and electrode footprint‐normalized capacitances (>3 F cm^−2^), demonstrating the need for hierarchical electrode architectures. However, excessive macroporosity in fiber‐based electrodes cause low volumetric loadings (tap density < 0.35 g cm^−3^), and development of application‐relevant and ‐tailored electrodes requires a clear understanding of correlations and structure–performance relationships between capacitance, rate capability, electrode thickness, and meso‐ and macroporosity.

## 3 BCP‐Derived Battery Electrode Materials

The two key metrics of performance for batteries are energy and power (charge/discharge rate), normalized by mass or volume of the battery.^[^ ^9^ ^]^ While the energy of a battery is defined by the capacity of the electrodes, their electrochemical potential difference (voltage), and the fraction of those materials within the cell, the power is dominated and limited by multiscale mass transport.^[^ ^106^ ^]^ The structure from the nano‐ to macroscale of the electrode and the battery has therefore a defining impact on its performance metrics. Beyond geometrical considerations, nanosizing of active charge‐storing materials has enabled capacity increases for some, and even the cycling of other materials that are irreversible at larger dimensions, e.g., due to mechanochemical degradation.^[^ ^19^ ^]^ Understanding both the intricate correlation between local mass transfer, reaction kinetics, and porosity, as well as the relationship between phase behavior during solid‐state reduction/oxidation and nanostructural features, requires systematic studies of composition–structure–property relationships in synthetically tunable systems. In this section, we highlight research that demonstrates the utility of BCP self‐assembly to aid with the understanding and successful utilization of advanced electrode materials batteries, starting with the negative electrode (anode: 3.1–3.7, **Table** **4**) followed by cathode materials (3.8–3.10, **Table** **5**).

**Table 4:** BCP‐derived anode materials with systematic variations in composition and structural parameters

Material			Porosity				Setup	Performance (*not stable in cycling)					References
Composition (wt ratio)	Polymer	Morphology	Pore size [nm]	Pore volume <100 nm cm^−3^ g^−1^	Nanoporosity	Surface area [m^2^ g^−1^]	Loading [mg cm^−2^]	Low‐rate capacity (≈0.1 C)/[mAh g^−1^]	Medium‐rate capacity (≈1 C)/[mAh g^−1^]	High‐rate capacity (5–1 °C) [mAh g^−1^]	Capacity retention at medium rate	Capacity retention at high rate	
TiO_2_–C (84:16)	PI‐*b*‐PEO	Disordered cylinders	17	0.3	52%	154	–	200	140	70.0	70%	35%	[112]
TiO_2_	Pluronics	Microparticles with disordered cylindrical mesopores	3	0.18	43%	201	0.7	240	180	60	75%	25%	[113]
	PS‐*b*‐PEO		7	0.16	40%	65	0.7	220	170	110	77%	50%	
			12	0.15	39%	38	0.7	200	160	100	80%	50%	
Li_4_Ti_5_O_12_–C (90:10)	PI‐*b*‐PEO	Disordered cylinders	20	0.148	34%	69	–	145	136	115	94%	79%	[121]
TiNb_2_O_7_	PS‐*b*‐PEO	Hierarchical macro/mesoporous	25	0.23	49%	60	1.1–1.4	257	240	190	93%	74%	[158]
		Disordered mesoporous	28	0.21	46%	57	1.1–1.4	210	190	145	90%	69%	
	PS‐*b*‐PEO	HEX	40	0.37	60%	74	1.1–1.4	289	240	180.0	83%	62%	[122]
Nb_2_O_5_–C (89:11)	PS‐*b*‐PEO	HEX	30	0.25	50%	76	0.4–1.4	200	150	80	75%	40%	[90]
Nb_2_O_5_		HEX	50	0.3	58%	40	0.4–1.4	182	150	110	82%	60%	
WO_*x*_/C (8:2)	PS‐*b*‐PEO	HEX	30	0.2	49%	48	1.0–1.5	613	290	100	47%	16%	[124]
WO_3_		HEX	30	0.13	48%	29	1.0–1.5	373	118	0	32%	0%	
NbS_2_–C (64:36)	PI‐*b*‐PS‐*b*‐PEO	Double‐gyroid matrix (powder)	12	0.33	50%	390	1.3	400	215	20	54%	5%	[130]
		Double‐gyroid matrix monolith	12	0.33	50%	390	21.6	110	15	–	14%	–	
NiCo_2_O_4_	PMPEGMA‐*b*‐PBA	Disordered	9	0.14	46%	19	0.05	–	754*	–	–	–	[206]
		Disordered	28	0.13	44%	35	0.05	–	754*	–	–	–	
Sn–@C–SiO_2_ (8.5:91.5)	PS‐*b*‐PEO	HEX Sn NW in C–SiO_2_ matrix	17	0.36	76%	427	–	460	350	175	76%	38%	[135]
Sn–@C–SiO_2_ (49:51)		Sn nanoparticles in C–SiO_2_ matrix	none	0.18	49%	326	–	690	400	200	58%	29%	

**Table 5:** BCP‐derived cathode materials with systematic variations in structural properties

Material			Porosity				Setup	Performance (*not stable in cycling)					References
Composition (wt ratio)	Polymer	Morphology	Pore size [nm]	Pore volume <100 nm cm^−3^ g^−1^	Nanoporosity	Surface area [m^2^ g^−1^]	Loading [mg cm^−2^]	Low‐rate capacity (≈0.1 C) [mAh g^−1^]	Medium‐rate capacity (≈1 C) [mAh g^−1^]	High‐rate capacity (5‐1 °C) [mAh g^−1^]	Capacity retention at medium rate	Capacity retention at high rate	
LiFePO_4_/C	PI‐*b*‐PEO	Disordered micellar	40–100	0.23	26%	36.3	0.75 ± 0.24	165	155	135	94%	82%	[149]
Sulfur/carbon (1:1)	ISO	Double gyroid	15	0.8	18%	318	>0.8	425	300	–	71%	–	[155]
		Double gyroid	16	0.89	22%	606	>0.8	525	375	–	71%	–	
		Double gyroid	15	1.32	36%	1076	>0.8	450	–	–	–	–	
		Double gyroid	14	2.45	57%	2029	>0.8	600	475	–	79%	–	
		Double gyroid	39	1.15	31%	202	>0.8	500	–	–	–	–	
		Double gyroid	39	1.56	42%	692	>0.8	475	–	–	–	–	
		Double gyroid	40	2.25	55%	1341	>0.8	350	–	–	–	–	
		HEX	25	0.46	0%	167	>0.8	350	–	–	–	–	
		Alternating gyroid	31	1.41	39%	348	>0.8	350	–	–	–	–	
Sulfur/carbon (1:2)	PS‐*b*‐P4VP	Double gyroid	20	1.4	32%	885	–	667	287	–	43%		[156]
Sulfur/TiN (7:3)	PS‐*b*‐PEO	Hierarchical macro/mesoporous	32	0.3	0%	47	1.5–2.0	1040	800	664	77%	64%	[157]
Sulfur/TiN (7:3)		HEX	37	0.3	0%	66	1.5–2.0	750	600	150	80%	20%	

### 3.1 BCP‐Derived Titania Anode Materials

Titanium(IV) oxide (“titania”) is a transition metal oxide of interest as active anode material in LIBs. Titania is attractive due to its abundance, low cost, low toxicity, and compatibility with various nanostructuring methods.^[^ ^107^ ^]^ Amorphous and anatase titania are studied predominantly for EES. Micrometer‐sized anatase converts to lithium titanate with a maximum of around 0.5 lithium per titanium (Li_0.5_TiO_2_) upon lithiation at room temperature, corresponding to a theoretical gravimetric capacity of 168 mAh g^−1^.^[^ ^108^ ^]^ For nanosized anatase, up to one lithium per titanium can be stored, effectively doubling the specific capacity purely due to nanostructuring.^[^ ^109^ ^]^ Furthermore, the small length scales of nanotitania allows for high electrolyte accessibility and short solid‐state ion‐diffusion distances, increasing reaction kinetics, rate capability, and power density. Due to the inherently low electronic conductivity of titania, however, compositing or hybridization with conductive materials such as carbon is necessary.

Numerous hydrolytic and nonhydrolytic sol–gel syntheses of nanostructured mesoporous titania have been reported using BCP self‐assembly.^[^ ^40^, ^104^, ^105^, ^110^ ^]^ In particular, the coassembly of oxide precursors with BCPs containing *sp* ^2^‐hybridized carbon converting to carbonaceous material upon pyrolysis (CASH method) is an attractive one‐pot method to obtain highly crystalline nanostructures percolated or coated with electronically conductive carbon material.^[^ ^40^ ^]^ The reverse process, coating OMCs with titania using top‐down methods such as atomic layer deposition (ALD), has been demonstrated to be feasible only in thin films.^[^ ^111^ ^]^ The impact of the carbon on the electrochemical performance of these ordered mesoporous titania–carbon hybrids can be directly studied by removing it through mild calcination in air.

**Figure 8:**
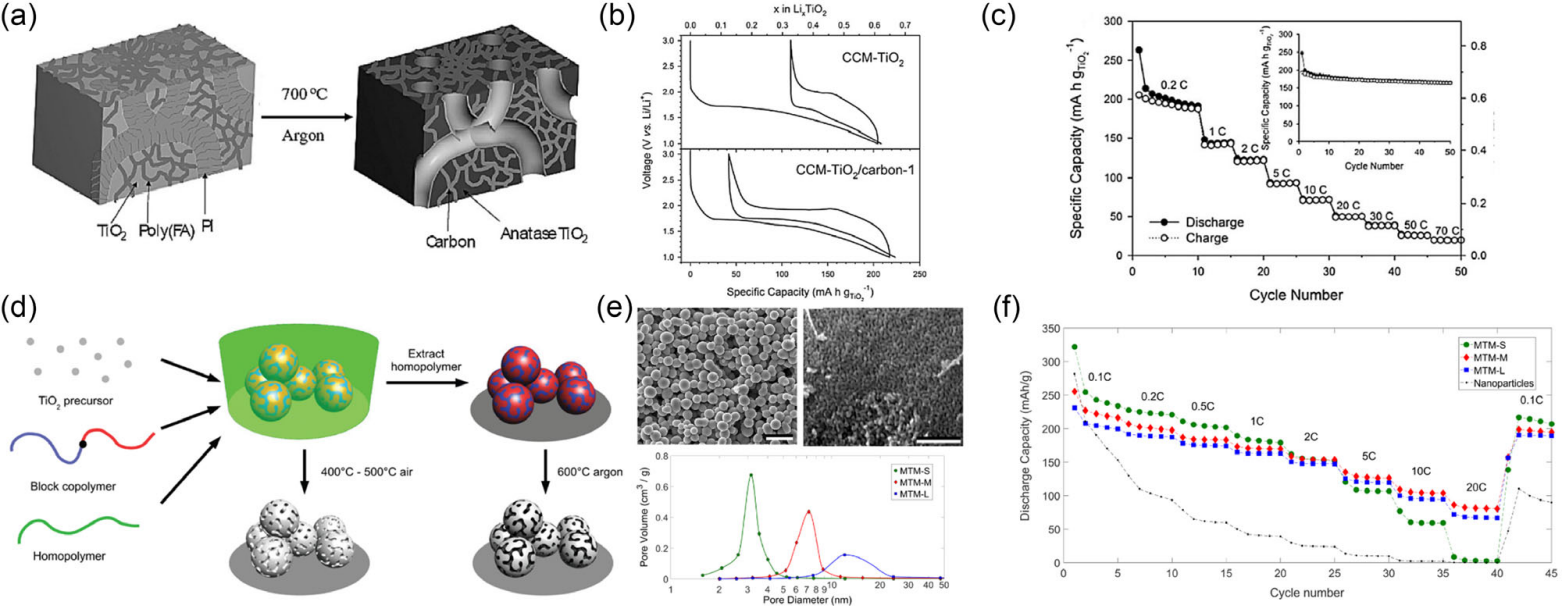
a–c) Mesoporous titania–carbon composites. (a) Schematic of “one‐pot” synthesis through the carbonization of mesostructured PI‐*b*‐PEO/amorphous TiO_2_/poly(furfuryl alcohol) hybrids. (b) First galvanostatic discharge–charge curves at 0.2 C of mesoporous titania (top) and titania–carbon hybrid (bottom). (c) Galvanostatic rate and cycling (inset) performance of mesoporous titania–carbon hybrid. (a–c) Adapted with permission.^[^ ^112^ ^]^ Copyright 2011, Wiley‐VCH. d–f) Mesoporous titania microspheres (MTM). (d) Schematic of “one‐pot” synthesis through dual polymer micro‐ and macrophase separation and BCP–titania coassembly followed by heat treatment. (e) SEM images at different magnifications (top; scale bars: left: 5 μm; right: 200 nm) and BJH pore size distribution from nitrogen sorption measurements (bottom) of MTMs derived from different molar mass BCPs for various mesopore sizes (S: small, M: medium, L: large). (f) Galvanostatic discharge capacities at various rates of MTMs with different mesopore sizes and of nontemplated titania nanoparticles. (d–f) Adapted with permission.^[^ ^113^ ^]^ Copyright 2017, American Chemical Society.

Amorphous and anatase titania–carbon nanocomposites with pore sizes of a few nanometers to over 20 nm from the self‐assembly of Pluronics and custom‐made BCPs have been studied as anode material in LIBs,^[^ ^112^, ^113^, ^114^, ^115^, ^116^ ^]^ and sodium‐ion batteries.^[^ ^117^ ^]^ Lee et al. reported the use of mesoporous anatase–carbon hybrids using PI‐*b*‐PEO as structure‐directing BCP in combination with nonhydrolytic titania sol–gel chemistry and furfuryl alcohol as a carbon precursor.^[^ ^112^ ^]^ The heat‐treated hybrid contained anatase nanocrystallites “wired” with carbonaceous material from the furfuryl alcohol with mesopores of 17.4 nm diameter coated with a thin graphitic carbon layer resulting from the PI degradation, as illustrated in **Figure** **8**a. The work demonstrated the effects of titania porosity and nanostructuring on electrochemical performance. The small crystallite size of the anatase leads to a high lithium insertion capacity of 0.75 per titanium, as compared to only 0.15 for nonporous anatase. The presence of nanopercolating carbon decreased the irreversible capacity loss after the first cycle from more than 50% to 20%, as shown in Figure 8b. The four orders of magnitude increased conductivity of the titania–carbon composite yielded better rate capability and higher reversibility compared to the same material without carbon (Figure 8c). The retention of almost 20% capacity at a rate of 30 C (2 min theoretical (dis)charge) is particularly noteworthy for a material with low overall porosity and surface area of 0.3 cm^3^ g^−1^ and 154 m^2^ g^−1^, respectively.

High porosity, small material dimensions, and large surface area typically enable high‐rate capability due to increased electrolyte access and decreased charge‐transfer resistance. However, high porosity lowers the overall volumetric capacity of electrodes and high surface area anodes cause excessive side reactions with the electrolyte leading to poor efficiency. A balance between porosity, surface area, connectivity, and content of active material is therefore crucial to achieve high‐performing electrodes, and the required specific and volumetric energy and power is often application dependent, opening the opportunity for demand‐tailored electrode architectures. The tunable mesoporosity and nanoscopic materials’ dimensions, distributions, and proximity in the form of macroscopic hybrids makes BCP‐derived anode materials attractive for battery applications, but nanoscale control offered by traditional BCP coassembly is not sufficient to address all aspects, especially ion transport in the electrolyte through thick electrodes.

To address the balance of macroscopic electrode architecture of mesoporous active materials, Fischer et al. synthesized mesoporous titania and titania–carbon hybrid microspheres from combined BCP self‐assembly and polymer macrophase separation.^[^ ^113^ ^]^ In this process, BCPs and titania sol–gel precursors are coassembled by solvent evaporation in the presence of large homopolymers that exhibit no interaction with the other components.^[^ ^118^ ^]^ During solvent evaporation, the coassembling BCP and inorganic precursor domains macrophase separate from the homopolymer as spherical inclusions. Upon selective removal of the homopolymer and BCP via heat treatment in air or inert atmosphere, crystalline mesoporous titania or titania–carbon hybrid microspheres were obtained, respectively, with pore sizes of 3, 7, and 12 nm tunable by the choice of nanostructure‐directing BCP (Figure 8d,e). The high surface area of the 3 nm pore size titania microspheres obtained from Pluronics caused irreversible lithium insertion and fast capacity fading during cycling and at higher rates. The mesoporous titania microspheres with 7 and 12 nm obtained from custom‐made PS‐*b*‐PEO exhibited good cyclability and rate capability with over 100 mAh g^−1^ at a rate of 1 C, as shown in Figure 8f. Interestingly, the study showed very little influence of the in situ formed carbon on the electrochemical properties, except a reduction in anatase crystallite size and corresponding slight increase in the lithium insertion capacity. It is possible that the low temperature employed during heat treatment and carbonization (600 °C) yielded a low degree of graphitization and, therefore, insignificant differences in electronic conductivity between microspheres with and without carbon. Importantly, the study demonstrated that hierarchical structures such as mesoporous microspheres enable the use of mesoporous electrode materials at high packing fraction in a hierarchical electrode architecture leading to application‐relevant energy and power densities at the cell level.

### 3.2 BCP‐Derived LTO and Mixed Titanium Oxide Anode Materials

**Figure 9:**
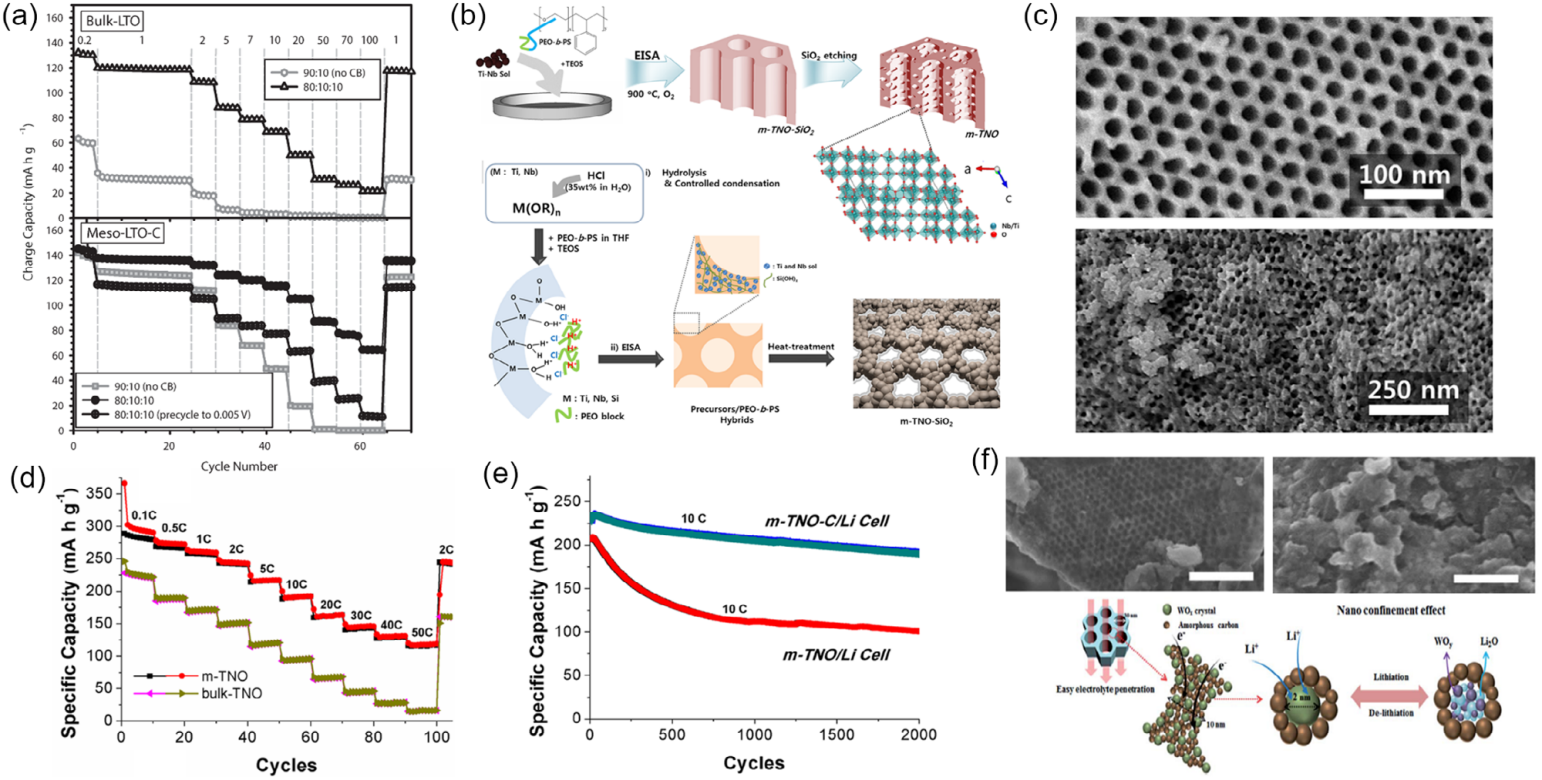
a) Galvanostatic discharge capacities at various rates of nontemplated LTO (top) and mesoporous LTO–carbon composites (bottom) with (black) and without (gray) carbon additives. Adapted with permission.^[^ ^121^ ^]^ Copyright 2011, Wiley‐VCH. b–e) Mesoporous titanium‐niobium oxide (m‐TNO) anode. b) Scheme of EISA of a structure‐directing BCP with titanium, niobium, and silica precursors and subsequent heat treatment and silica etching. c) SEM images of m‐TNO before (top) and after (bottom) silica etching. d) Galvanostatic discharge capacities at various rates of m‐TNO and bulk TNO demonstrating the beneficial effect of mesoporosity. e) Galvanostatic discharge capacities of m‐TNO with (blue) and without (red) postsynthesis carbon coating demonstrating its beneficial effect at high rates. (b–e) Adapted with permission.^[^ ^122^ ^]^ Copyright 2014, American Chemical Society. f) Ordered mesoporous tungsten oxide (m‐WO_3_) anodes. Top: SEM images of m‐WO_3_ after 30 charge–discharge cycles with (left) and without (right) carbon hybridization (scale bars: 250 nm). Bottom: Scheme of the mesoporous m‐WO_3_/carbon nanohybrid with its proposed beneficial confinement effect for enhanced cycling stability. Reproduced with permission.^[^ ^124^ ^]^ Copyright 2017, Royal Society of Chemistry.

LTO (Li_4_Ti_5_O_12_) is a very attractive anode material in LIBs for its zero‐strain and fast lithium‐ion insertion.^[^ ^119^ ^]^ The theoretical reversible capacity of 3 lithium ions per unit formula, corresponding to 175 mAh g^−1^, at a potential of around 1.5 V versus Li/Li^+^ limits the energy density of LTO anodes compared to others. At the same time, the higher reduction potential mitigates the risk of lithium metal deposition and reduces unwanted electrolyte side reactions such as solid electrolyte interface formation.^[^ ^120^ ^]^ Thus, LTO is gaining interest as an anode material for high power LIBs, and can already be found in commercial devices. However, the low electronic conductivity necessitates the hybridization of LTO with conductive material to fully utilize its high‐rate capabilities. To this end, Kang et al. employed the CASH method to synthesize mesoporous LTO with a custom‐made PI‐*b*‐PEO (30 kg mol^−1^) as the structure‐directing BCP, and titanium tetraisopropoxide, lithium ethoxide, and oxalic acid as the sol–gel LTO precursors.^[^ ^121^ ^]^ The large mesopores of 20 nm were lined with graphitized carbon formed by the PI block during pyrolysis of the coassembled hybrid at 700 °C under inert atmosphere. The carbon layer contributed less than 10 wt% to the mesoporous hybrid, but enabled the material to be cycled at 0.2 C (5 h theoretical discharge) without any conductive additives. The material exhibited exceptional high‐rate operation with additional 10 wt% carbon black: over 50% capacity retention at a rate of 10 C (36 s theoretical discharge) and over 100 mAh g^−1^ at 1 C with only 10% capacity loss over 500 cycles, as shown in **Figure** **9**a.

To increase the capacity for high‐rate titanium‐based anodes, mesoporous ternary and mixed titanium oxides such as TiNb_2_O_7_ (TNO) and TiO_2_–SnO_2_ structure directed by BCPs have been reported.^[^ ^122^, ^123^ ^]^ The insertion of five lithium ions into TNO in the potential range of 1–2 V versus Li/Li^+^ yields a theoretical capacity of 388 mAh g^−1^, well above the capacity of LTO but with the same safety and stability advantages of a higher reduction potential. Jo et al. synthesized mesoporous crystalline TNO with a large pore size of 40 nm using PS‐*b*‐PEO and silica tetraorthosilicate as a silica precursor in addition to the hydrolytic transition metal sol–gel mixture. The addition of silica endows the coassembled nanostructure with high thermal stability, enabling crystallization of TNO at 900 °C and the retention of the highly ordered hexagonally packed nanostructure without the use of the CASH method, as illustrated and shown in Figure 9b,c. The silica is removed by a facile postsynthesis sodium hydroxide etch. The highly ordered and crystalline mesoporous TNO exhibited remarkable rate capability with 190 mAh g^−1^ at 1 C and 116 mAh g^−1^ at 5 C (Figure 9d). When a postsynthesis carbon coating was applied, the ordered mesoporous TNO could be cycled with an exceptional stability, still exhibiting a capacity of 191 mAh g^−1^ after 2000 cycles at 1 C, as shown in Figure 9e.

### 3.3 Other BCP‐Derived Transition Metal Oxide and Sulfide Anode Materials

The use of BCPs to obtain numerous other mesoporous electrochemically active metal oxides has been demonstrated. Large BCPs enable accessible and continuous pores with sizes of 5–50 nm while providing sufficient wall thickness between the pores to enable the formation of nanocrystallites of 5–30 nm. Nanosized crystallites can suppress unwanted phase transformations and accommodate mechanical stresses better during ion insertion/desertion and conversion reactions than larger crystals. For example, tungsten oxide (WO_3_) is considered an affordable anode material for LIBs with high volumetric energy storage capability due to its high density. During the lithium‐ion intercalation‐initiated conversion reaction to tungsten metal, six lithium ions per unit formula can theoretically be stored, corresponding to a capacity of 694 mAh g^−1^ or 4966 mAh cm^−3^. By coassembling PS‐*b*‐PEO and tungsten hexachloride with and without resols, Jo et al. studied the effect of nanodispersed carbon on the EES performance of highly ordered hexagonally packed mesoporous WO_3_ with large pore sizes around 30 nm.^[^ ^124^ ^]^ The highly dispersed carbon from the resols confines and limits the metal oxide crystallite size in the 10 nm‐thick walls, leading to a significantly increased cycling performance with a capacity of 443 mAh g^−1^ after 100 cycles at 250 mA g^−1^. The study demonstrated that the nanodispersed carbon phase not only reduces the internal resistance of the hybrid material and enables better rate capability, but also induces a less detrimental solid–electrolyte interface formation compared to the pure oxide. Furthermore, postmortem analysis of the electrode materials showed the collapse of the ordered nanostructure for the pure oxide material, while it was preserved during cycling for the ordered WO_3_–carbon hybrid, as shown in Figure 9f. The thorough analysis revealed the multitude of beneficial properties the nanodispersed carbon adds to ordered mesoporous oxides, including lower electronic resistance, crystal growth confinement, interface modification, and structural stabilization.

**Figure 10:**
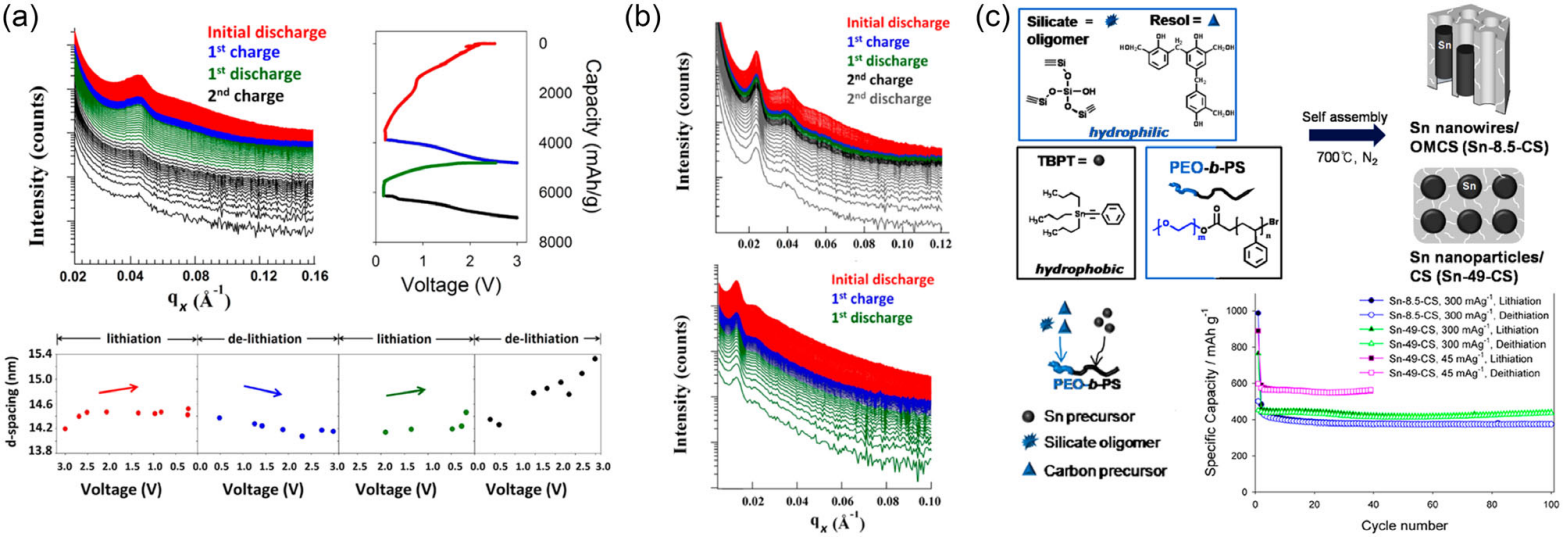
a,b) 1D operando grazing incidence small‐angle X‐ray scattering (GISAXS) traces of mesoporous NiCo_2_O_4_ thin film anodes with (a, top) 9 nm, (b, top) 16 nm, and (b, bottom) 28 nm pore size during galvanostatic cycling showing the retention of the mesostructure only for the larger pore sizes. Adapted with permission.^[^ ^206^ ^]^ Copyright 2017, American Chemical Society. (a, bottom) Changes in *d*‐spacing over the first two cycles of the mesoporous NiCo_2_O_4_ thin film anodes with 9 nm pore size showing the complete loss of structure in the second delithiation. c) Tin‐embedded carbon–silica composites from BCP coassembly with hydrophilic and hydrophobic precursors of carbon/silica and tin, respectively: scheme of the “one‐pot” synthesis (top) and galvanostatic discharge capacity (bottom right) with 8.5 wt% (blue circles) and 49 wt% (green triangles and pink squares) tin loading at various rates. (a–c) Adapted with permission.^[^ ^135^ ^]^ Copyright 2013, American Chemical Society.

Later transition metal oxides such as iron, cobalt, and nickel oxide undergo insertion and conversion reactions with alkali metal ions at low electrochemical potentials, rendering them attractive as anode materials for their ease of fabrication and low cost.^[^ ^125^, ^126^, ^127^, ^128^ ^]^ As with other electrochemical reactions, mesoporosity and nanosized material dimensions enable good electrolyte accessibility and buffering of mechanical stresses during charge and discharge. For example, mesoporous nanocrystalline α‐Fe_2_O_3_ (hematite), structure directed KLE BCPs, could insert more lithium ions than bulk materials.^[^ ^125^ ^]^ The nanostructured thin film exhibited reversible intercalation of up to 1 lithium ion per unit formula without the detrimental and irreversible phase transformation to Li_*x*_Fe_2_O_3_ that is already observed for microcrystalline hematite at 10 times less lithium content. Utilizing a similar BCP micelle templating, Vogt et al. studied mesoporous films of ternary Ni_*x*_Co_3−*x*_O_4_ (*x* = 1, 1.5, 2) as sodium‐ion anode material.^[^ ^126^ ^]^ The use of micelles instead of homogeneous BCP self‐assembly enables the structure direction of different materials of varying composition but constant nanostructure and pore sizes.^[^ ^55^ ^]^ The ternary nickel cobalt oxides demonstrated relatively poor cycling performance as sodium‐ion anodes, but the uniform structural features across all compositions allowed the conclusion that Ni_2_CoO_4_ exhibited the best (electro)chemical stability of the studied compositions. The same group studied a similar BCP micelle‐templated NiCo_2_O_4_ system as lithium‐ion anode with operando wide‐ and small‐angle X‐ray scattering. By employing BCPs with differently sized hydrophobic block lengths, thin films of NiCo_2_O_4_ with pore sizes of 9, 16, and 28 nm and increasing mesopore wall thickness were used as conversion anodes. The study found that an almost complete loss of mesoporosity in the first lithiation for the 9 nm pore size materials accompanied by irreversible formation of Li_2_O caused significant capacity loss within 2 cycles. The larger pore‐sized materials with thicker pore walls retained their mesoporosity and exhibited less irreversible lithium insertion over multiple cycles, leading to better cyclability, as shown in **Figure** **10**a,b. The mesostructures of all samples were significantly distorted during the reaction with lithium, while the material phase segregated on the atomic scale into mostly amorphous nickel and cobalt oxide after lithium extraction. The report offers insight into the importance of maintaining mesoporosity in conversion electrodes during cycling. However, the micelle templating method yields materials with relatively poor long‐range order, making quantitative conclusions difficult. Applying operando X‐ray scattering to highly ordered Ni–NiO gyroid electrodes may offer even more insight into the relation between the evolution of atomic and mesostructural order during electrochemical cycling, and their impact on electrode performance metrics.^[^ ^128^ ^]^

Templating of 2D van der Walls materials such as metal dichalcogenides into ordered nanostructures has been difficult to achieve and only poorly explored, despite a strong interest for their use in energy storage devices.^[^ ^129^ ^]^ While exfoliated 2D materials easily form highly porous materials, controlling their porosity to balance energy density with material accessibility for the electrolyte is challenging due to a lack of suitable precursor chemistry that is compatible with soft templating. In an interesting BCP‐templating and thermochemical conversion approach, Fleischmann et al. have recently demonstrated the synthesis of gyroidal niobium disulfide (NbS_2_).^[^ ^130^ ^]^ Their process combined the known coassembly of ISO with niobium ethoxide (EISA) with pyrolysis and sulfidization under hydrogen atmosphere with elemental sulfur upstream, yielding monolithic materials with NbS_2_ nanocrystals engulfed in a highly porous gyroidal carbon shell. The gyroidal composite was discharged to low potentials (0.05 V vs Li/Li^+^) inducing a conversion reaction with initially over 450 mAh g^−1^ capacity, but showed significant capacity loss over cycling. Additionally, a purely mesoporous NbS_2_–C monolith was employed as a binderless electrode with high volumetric capacity (≈150 mAh cm^−3^), but only at low rate of ≈C/10. Hence, in addition to extending the vast literature on BCP‐derived metal oxides to commonly hard‐to‐template metal sulfides by using posttemplating thermochemical conversion with retention of the ordered nanostructure, this study illustrates the opportunity and challenges of translating mesoporous materials to application‐relevant electrodes with practical loadings (thickness) and energy density. The encountered severe mass‐transfer limitations in electrodes of 10–100s of micrometers in thickness with only sub‐micrometer porosity for application‐relevant rates will only be overcome through introduction of additional macroporosity in hierarchical architectures.

### 3.4 BCP‐Derived Alloying Anode Materials

Lithium alloying materials have gained significant interest as potential anodes for their ultrahigh capacity. Silicon lithium alloys in particular have gathered significant attention due to their abundance and high gravimetric capacity.^[^ ^131^ ^]^ However, the lithium intake is accompanied by an expansion of multiple times the initial silicon volume, leading to pulverization of the material. These challenges are mitigated with the use of nanosized silicon such as nanowires to accommodate the volume expansion during cycling. Interestingly, templated mesoporous silicon from BCP self‐assembly has been reported, but not yet tested as lithium‐ion anodes.^[^ ^132^, ^133^, ^134^ ^]^ We believe that the higher effective density in BCP‐derived mesoporous silicon paired with continuous electronic pathways of nanonetwork architectures may prove advantageous. Other lithium‐alloying materials such as BCP‐templated germanium and tin have been reported as anodes.^[^ ^135^, ^136^ ^]^ Mesoporous GeO_2_, GeO_2_–carbon, and Ge–GeO_2_–carbon hybrids with a large pore sizes around 40 nm were obtained using PS‐*b*‐PEO coassembly with resols and hydrolytic germanium sols as carbon and germanium oxide precursors, respectively. The oxide could be partially reduced to elemental germanium with mild reductive heat treatment, a synthetic advantage of germanium over silicon. The incorporated carbonaceous material supplies structural support during heat treatment and BCP decomposition, prevents excessive crystal growth of the inorganic component, and significantly increases the electronic conductivity. Interestingly, the Ge–GeO_2_–carbon hybrid material exhibited the highest coulombic efficiency of 72.5% in the first cycle and over 95% over subsequent cycles combined with the highest reversible capacity of over 1000 mAh g^−1^ after 90 cycles. The authors studied the mechanism of the electrochemical reactions in detail and concluded that the mesostructure in combination with highly dispersed nanosized germanium and carbon enabled the efficient and reversible lithium extraction from Li_2_O that forms during GeO_2_ reduction and germanium reoxidation during charging. Furthermore, the high area density afforded by the one‐pot BCP templating method led to the highest reported areal capacity for germanium‐based anodes at the time.

BCP coassembly is typically achieved using hydrophilic precursors of the desired functional material that selectively interact with the hydrophilic block of an amphiphilic BCP such as PEO or P4VP and get incorporated into the hydrophilic domain of the final self‐assembled nanostructure. However, the hydrophobic phase can also be used to incorporate functional materials, yet typically at lower fractions due to the weaker compatibilizing power of hydrophobic interactions. Utilizing such a multimaterial multiphase structure direction concept, Hwang et al. synthesized tin‐embedded carbon–silica composites using the coassembly of PS‐*b*‐PEO with hydrophilic carbon and silica precursors and hydrophobic tributylphenyl tin, which selectively incorporates into the PS phase.^[^ ^135^ ^]^ With increasing amounts of tin precursor, an ordered mesoporous hexagonal structure, a macrophase separated morphology, and a nonporous, homogeneous micellar morphology were produced, as illustrated in Figure 10c. Interestingly, both the ordered mesoporous hybrid with 8.5 wt% tin and the micelle‐derived nonporous hybrid with 49 wt% tin exhibited excellent reversible capacity retention of around 90% their theoretical capacity over 100 cycles (Figure 10c). This work demonstrated that control is gained beyond nanostructural features to include compositions of composite electrodes that may result in various avenues to stabilize conversion anode materials as nanostructures while ensuring a sufficient electrode density to achieve application‐relevant energy densities and specific energies.

### 3.5 BCPs to Control Plating in Lithium Metal Batteries

The use of lithium metal as the anode is a highly desired step for next‐generation batteries because the anode could effectively take up an ultrasmall fraction of volume and mass within the cell.^[^ ^137^ ^]^ Lithium metal anodes would especially be game changing if paired with high‐capacity cathodes such as sulfur.^[^ ^138^ ^]^ However, the inhomogeneous plating of lithium during the charging process causes the formation of dendrites, which leads to both loss of lithium (“dead lithium”) and internal shorting of the battery over cycling.^[^ ^139^ ^]^ It has been predicted that these degradation and safety issues may be overcome if controlled and homogeneous lithium‐ion transport at the anode–electrolyte interface can be established.^[^ ^140^, ^141^, ^142^ ^]^ Over the last one to two decades, the homogeneiety and high porosity of BCP‐derived membranes have been employed to precisely control mass transport of species in water filtration.^[^ ^143^, ^144^, ^145^ ^]^ In an analogous approach, Han et al. recently demonstrated that PS‐*b*‐PMMA‐derived thin films with homogeneous vertical pore channels of 15 nm diameter at high pore density can act as a mass‐transfer homogenizing protective layer for lithium metal deposition.^[^ ^146^ ^]^ After UV cross‐linking of the PS and removal of PMMA by reactive ion etching, a multilayer architecture of silver NPs (Ag‐NPs) reduced graphene oxide (rGO), and the mesoporous PS thin film was established on copper foil. Dense lithium plating below the rGO‐PS layers was observed up to 3 mAh cm^−2^ only if all three layers were employed, indicating that lithophilicity (Ag‐NPs) in combination with homogeneous electronic conductivity and lithium‐ion transport is required. This report demonstrated that homogeneous mesoporous BCP‐derived structures could serve as separators that enforce homogeneous mass transfer at the sub‐100 nm scale, a distinct departure from the widely studied concept of BCP solid electrolytes such as PS‐*b*‐PEO. However, polymer separators of 10s of micrometers in thickness, as commonly employed in LIBs, with only mesoporosity would represent a severe barrier to lithium‐ion transfer and detrimentally increase cell resistance. As the homogenizing effect for lithium mass transfer is only required locally at the anode–electrolyte interface, separators akin to asymmetric BCP filtration membranes that utilize BCP self‐assembly combined with nonsolvent‐induced phase separation to produce membranes with well‐ordered, densely packed, uniform pores in the top surface could present an advanced separator solution for lithium metal batteries that ensures both high bulk and homogeneous local lithium‐ion transport.^[^ ^143^, ^144^ ^]^

### 3.6 BCP‐Derived Carbon Anodes for Sodium Batteries

Graphitic carbon has been the most used active anode material in commercial LIBs in the last three decades. While much research has focused on studying mesoporous and BCP‐derived carbon materials as supercapacitor electrodes, to date only limited efforts have been devoted to understanding the effects of BCP‐derived nanoarchitected carbon anodes in LIBs.^[^ ^147^, ^148^ ^]^ The push for batteries that utilize cheaper and more abundant materials than lithium, however, has led researchers to study BCP‐derived nanostructured carbons as anode material in sodium‐ion batteries. Sodium is unable to effectively intercalate into traditional graphitic carbon due to its larger ion size compared to lithium, but nongraphitic carbons are able to reversibly insert and extract sodium ions. Jo et al. studied the effect of nanostructure on the capacity, rate capability, and cyclability of OMC anodes in sodium‐ion batteries by using custom‐made BCPs of different molar mass as structure‐directing agent.^[^ ^147^ ^]^ Three OMCs with hexagonally packed cylindrical pores of 6, 33, and 60 nm diameter with wall thicknesses of 5, 5, and 8 nm, respectively, were synthesized and tested as sodium‐ion anodes. Interestingly, the OMC with 33 nm pore size and 5 nm wall thickness showed the highest reversible capacity at low rates and best capacity retention at high rates, even though electrochemical impedance spectroscopy (EIS) revealed decreased ion resistance in larger pores. The better rate capability of the 33 nm over the 60 nm pore size OMC was possibly due to the thinner carbon wall that influences the solid‐state sodium‐ion insertion kinetics. The results demonstrate the importance of not only pore size and surface area on capacity and kinetics, but also the nanoscaled dimension of the active material itself, i.e., its wall thickness. The deconvolution of all influential structural factors is often hard to obtain when studying nanomaterials, but BCP self‐assembly is a powerful tool to control morphological parameters individually to understand their influence on performance. For example, the total rate capability of battery electrodes is determined by factors, including solid‐state diffusion/reaction rates within the material, the ion diffusion within the porosity of the secondary particles (0.5–10 μm), and ion diffusion across the cell (50–200 μm). Thus, depending on the material, the secondary particle size, and the electrode/cell thickness, the optimal pore size for ion diffusion will differ.

### 3.7 BCP‐Derived Lithium Iron Phosphate Cathodes

**Figure 11:**
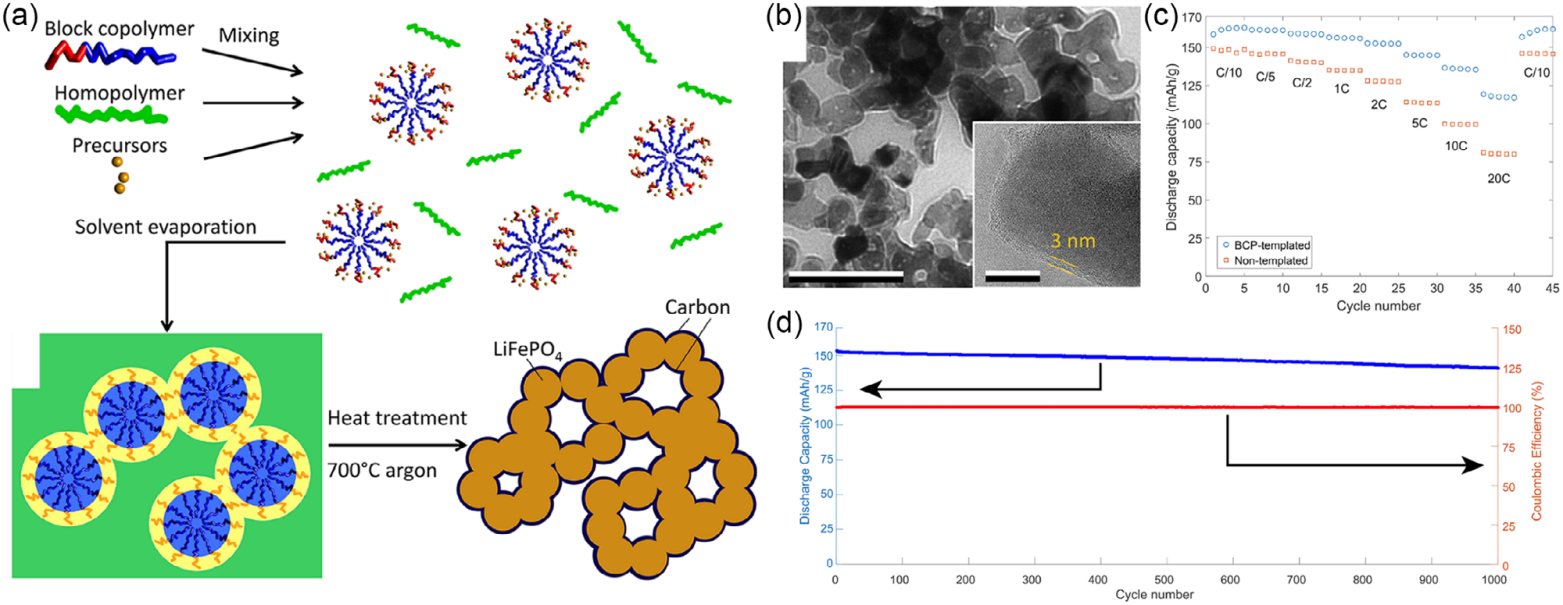
Polymer‐templated LiFePO_4_/C nanonetworks. a) Scheme of structure formation using BCP micelle templating of LiFePO_4_ in the presence of carbonizable homopolymer. b) Transmission electron microscopy (TEM) images of the crystalline carbon‐coated LiFePO_4_ nanonetwork. Scale bars are 200 and 20 nm (inset). c,d) Galvanostatic rate and cycling performance of BCP‐templated LiFePO_4_/C nanonetworks (c: blue circles at various rates; d: at 1 C) and nontemplated LiFePO_4_/C (c, red squares). (a–d) Adapted with permission.^[^ ^149^ ^]^ Copyright 2018, American Chemical Society.

Despite its lower capacity (170 mAh g^−1^) and low conductivity, lithium iron phosphate (LFP, LiFePO_4_) has emerged as a viable competitor to replace lithium cobalt oxide based materials as cathode material in LIBs, in part due to its increased safety and abundance, as well as high power capability when nanosized and coated with conductive material. The structural stability of the crystalline LFP framework during lithium insertion and extraction and short ion diffusion distances in nanosized LFP lead to high reversibility and rate capability during cycling, respectively. However, the low intrinsic conductivity necessitates intimate contact of the charge‐storing LFP with current collecting material. Carbon‐coated mesoporous and nanosized crystalline inorganic materials are accessible through BCP coassembly and calcination under inert atmosphere, i.e., the CASH method (vide supra).^[^ ^40^ ^]^ In an adaptation of the CASH method, Fischer et al. synthesized mesoporous LFP–carbon composites through the coassembly of PI‐*b*‐PEO with lithium chloride, iron acetylacetonate, and phosphoric acid in the presence of poly(styrene) homopolymer and crystallization at 700 °C under argon, as illustrated in **Figure** **11**a.^[^ ^149^ ^]^ The BCP and homopolymer provided intimate carbon coating and LFP crystallite growth restricted to 34 nm (Figure 11b), in contrast to hard‐templated or Pluronics‐derived LFP–carbon composites.^[^ ^150^, ^151^ ^]^ The small crystallite size and homogeneous carbon coating provided short lithium‐ion diffusion distances and electronic conductivity, respectively, enabling remarkable cyclability, efficiency, and rate performance, as shown in Figure 11c,d. At a rate of 20 C, the BCP‐derived LFP–carbon composite exhibited a reversible capacity of 117 mAh g^−1^ and retained 92% of its 1 C capacity over 1000 cycles.

### 3.8 BCP‐Derived Sulfur‐Composite Cathodes

One of the most intriguing novel cathode materials investigated over the last 20 years is elemental sulfur, due to its very high abundance, low toxicity, and high gravimetric capacity of 1672 mAh g^−1^. The high capacity and low price of sulfur make it an attractive cathode material for lithium‐based batteries despite its low discharge potential of 2–2.5 V versus Li/Li^+^. However, the complex redox chemistry of sulfur and the multiple species involved pose a significant challenge for its utilization. During the reduction of the nonpolar S_8_ molecules to the ionic Li_2_S, numerous lithium polysulfides (Li_*x*_S_*y*_; *x* = 1,2; *y* = 2–8) are formed, some of which are soluble in common electrolytes (*x* ≥ 4), leading to significant material and capacity loss upon repeated battery cycling. Additionally, sulfur and lithium sulfide are insulating, making the use of conductive additives such as carbon necessary. In 2009, Ji et al. demonstrated the use of OMC, obtained through hard‐templating from BCP‐derived ordered mesoporous silica, as a viable method to retain the soluble polysulfides in the carbon–sulfur nanohybdrid during charge and discharge while at the same time providing electronic conductivity.^[^ ^152^ ^]^ The high surface area (1976 m^2^ g^−1^) and pore volume (2.2 cm^3^ g^−1^) combined with the small pore size of 3–4 nm of the OMC provided intimate contact between the melt‐infused sulfur and the carbon host. The ordered nanohybrid containing a high 70 wt% sulfur loading exhibited unprecedented cycling performance, with over 80% retention after 20 cycles of the initial 1005 mAh g^−1^. Extensions of the system used OMC nanospheres and nanofibers obtained from the self‐assembly of the commercial Pluronics BCP F127 with tetraethylorthosilicate (TEOS) and phenyl–formaldehyde resols cast inside inverse opal and hollow nanofibers of silica, respectively.^[^ ^153^, ^154^ ^]^ The inclusion of silica in the mesoporous material provided additional microporosity after silica etching, and potentially left over polar functionality from incomplete etching, extending the lifetime of the cathode materials with a retention of over 700 mAh g^−1^ after 100 cycles.

**Figure 12:**
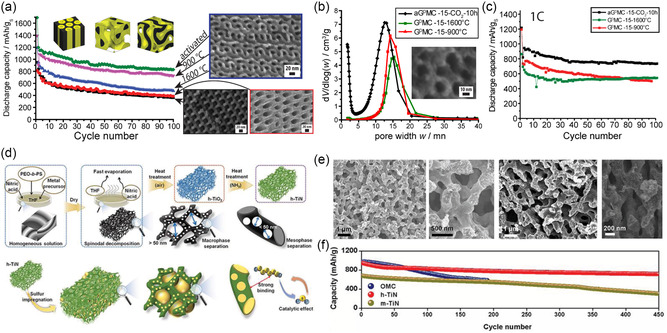
a–c) BCP‐derived ordered carbon–sulfur composites in lithium–sulfur batteries. a) Galvanostatic cycling performance at 0.1 C (left) and SEM images (right) of 1600 °C carbonized single gyroidal (black), hexagonally packed cylindrical (red), and double gyroidal mesoporous carbon (blue, G^D^MC), as well as 900 °C carbonized (purple) and activated (green) G^D^MC as sulfur host (1:1 wt). b) BJH pore size distribution from nitrogen adsorption and c) galvanostatic cycling performance at 1 C of 1600 °C (green), 900 °C (red) carbonized, and activated (black) G^D^MC. (a–c) Adapted with permission.^[^ ^155^ ^]^ Copyright 2015, American Chemical Society. d–f) Hierarchically porous titanium nitride (h‐TiN) as sulfur host in Li–S batteries. d) Scheme of the synthesis procedure utilizing BCP and titania–sol coassembly and nitric acid macrophase separation, followed by calcination, nitridation with ammonia, and sulfur infiltration into the meso‐ and macropores. e) SEM images of hierarchically porous h‐TiO_2_ (1st, 2nd), and h‐TiN (3rd, 4th). f) Galvanostatic cycling performance of h‐TiN (red), mesoporous TiN (gold, m‐TiN), and OMC (blue, OMC) at 0.5 C. (d–f) Adapted with permission.^[^ ^157^ ^]^ Copyright 2018, Wiley‐VCH

The importance of micro‐ and mesoporosity, as well as polar functionality, was further demonstrated by a comprehensive study on the impact of structural parameters on capacity retention and rate capability by Werner et al. Triblock terpolymers of PI‐*b*‐PS‐*b*‐PEO with custom‐tailored molar masses and compositions were employed to synthesize OMCs with various morphologies and pore sizes, and tested as sulfur hosts in lithium–sulfur batteries.^[^ ^50^, ^155^ ^]^ The morphologies included a single‐gyroid mesoporous network, hexagonally packed mesoporous cylinders, and double‐gyroid mesoporous matrices with pore sizes ranging from 15 to 39 nm. The highly ordered nanostructures were stable at carbonization temperatures up to 1600 °C. The wide range of accessible carbonization temperatures enabled the study of surface functionality of OMCs and its impact on lithium–sulfur batteries, as shown in **Figure** **12**a. Highly carbonized materials at 1600 °C contained lower amounts of oxygen, virtually negligible microporosity, and higher conductivity than their 900 °C analogues. Interestingly, the morphology and pore size of the studied carbons only showed minor impact on capacity retention, with the double gyroidal mesoporous carbon as the best performing high‐temperature OMC with a capacity of 489 mAh g^−1^ after 100 cycles. The carbonization temperature had a more significant impact on the capacity retention: at the lower carbonization temperature of 900 °C, the higher microporosity and oxygen surface functionality increased the reversible capacity by over 50% at 0.1 C. However, the lower conductivity reduced the rate capability of these carbons. Another aspect of the work demonstrated the successful use of physical activation using carbon dioxide gas at 950 °C subsequent to the high‐temperature carbonization at 1600 °C. The activation process introduced a high content of nanopores with sizes below 4 nm and increased the pore volume and surface area by factors of three and six, respectively, without mesostructure collapse (Figure 12b). The highly activated double gyroidal mesoporous carbon exhibited much better reversibility and rate performance than any of the nonactivated analogues with 830 and 731 mAh g^−1^ after 100 cycles at 0.1 and 1 C, respectively (Figure 12a,c). Another study on gyroidal mesoporous carbon as sulfur host derived from gyroidal mesoporous PS‐*b*‐P4VP through hard‐templating also demonstrated the beneficial effect of bicontinuous carbon hosts in combination with micro‐ and mesoporosity.^[^ ^156^ ^]^

Improving the chemical interaction between the conductive host and the polysulfide species increases capacity retention in lithium–sulfur batteries. Very recently, Lim et al. successfully demonstrated the applicability of their hierarchically ordered (macro/mesoporous) titanium nitride (h‐TiN) as conductive and strongly interacting sulfur host.^[^ ^157^ ^]^ The synthesis method combines the coassembly of PS‐*b*‐PEO and titania precursors with macrophase separation of nitric acid during EISA. The process enables the direct, one‐pot synthesis of titanium dioxide and other metal oxides as bicontinuous macroporous networks with mesoporosity within its sub‐micrometer‐sized network struts, as shown in Figure 12d,e.^[^ ^157^, ^158^ ^]^ h‐TiN was obtained after nitridation of the corresponding h‐TiO_2_ in ammonia at elevated temperatures without structural collapse. Despite the low mesopore volume of 0.3 cm^3^ g^−1^, the hierarchical titanium nitride exhibited excellent cycling performance with 745 mAh g^−1^ capacity after 450 cycles at 0.5 C, and 581 mAh g^−1^ capacity after 1000 cycles at 5 C, a 71.7% and 88% capacity retention, respectively (Figure 12f). The chemical bonding between the titanium nitride surface and various polysulfide species allows for their retention even with most of the sulfur present in macropores, which is in stark contrast to the less‐interacting carbon analogues.

### 3.9 BCP‐Derived Lithium–Oxygen Cathodes

The use of gaseous oxygen instead of solids as the active material on the cathode side of lithium batteries is very intriguing, due to the possibility of using environmental air. It is also very challenging, however, because the discharge intermediates and products are solid and highly reactive. The formation of various lithium oxides occurs on the surface of a porous current collector, often carbonaceous materials, leading to the irreversible formation of a variety of side products such as lithium carbonate. Mesoporous noncarbonaceous current collectors have the potential to avoid undesired side reactions as well as pore clogging, and provide sufficient accessibility to reactants and electrolyte. Kim et al. designed and synthesized ordered mesoporous titanium nitride from BCP self‐assembly with a large pore size of 37 nm and tested its performance as a lithium oxygen cathode in comparison to OMC with similar morphology.^[^ ^159^ ^]^ The titanium nitride with hexagonally packed porous cylinders was obtained from the corresponding PS‐*b*‐PEO‐derived titania through solid‐state reduction in ammonia at 700 °C. The mesoporous titanium nitride electrode exhibited significantly better electrochemical stability during constant potential and constant capacity cycling compared to its carbon analogue, leading to a higher reversible capacity and longer lifetime of the lithium oxygen batteries. However, the decomposition of the ether‐based liquid electrolyte and binder still caused build‐up of side products during extended operation, yet to a lesser extent than in carbonaceous materials. These side reactions could be suppressed using the redox mediator lithium iodide, extending the lifetime to over 280 cycles at constant 480 mAh g^−1^ capacity.

### 3.10 BCP‐Derived Solid‐State Cathode–Electrolyte Nanohybrids

**Figure 13:**
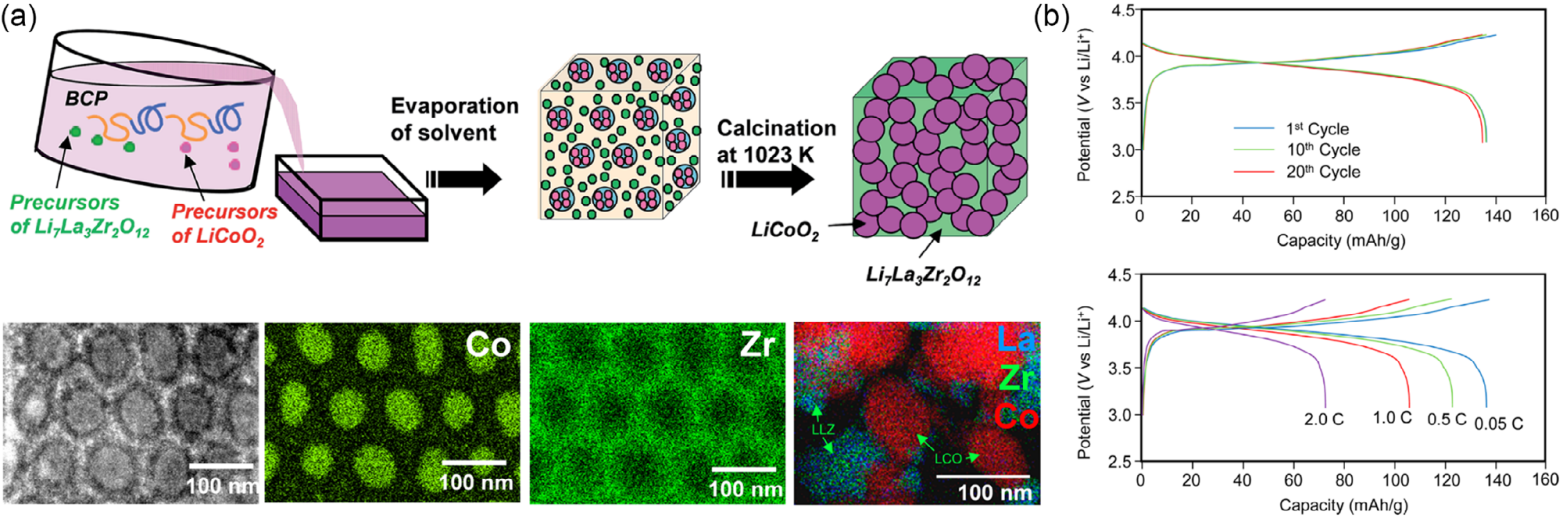
BCP‐derived bicontinuous LCO‐LZZO nanocomposite. a) Scheme of fabrication procedure (top) and dark‐field TEM image (bottom 1st) with corresponding energy‐dispersive X‐ray spectroscopy (EDS) map of cobalt (2nd) and zirconium (3rd) of the composite before heat treatment and an overlaid EDS map of the composite after calcination at 750 °C (bottom 4th). Adapted with permission.^[^ ^160^ ^]^ Copyright 2017, Royal Society of Chemistry. b) Galvanostatic charge–discharge curves at 0.05 C (top) and various rates (bottom) of the BCP‐derived LCO–LZZO solid‐state cathode. Reproduced with permission.^[^ ^161^ ^]^ Copyright 2016, American Chemical Society.

The commonly used liquid organic electrolytes in LIBs cause significant safety challenges due to their high volatility and flammability. Solid electrolytes such as lithium‐ion conducting polymers, ceramics, and glasses offer a safer alternative. Apart from lower ionic conductivity compared to their liquid counterparts, solid‐state electrolytes suffer from poor contact and high charge‐transfer resistance to the active charge‐storing electrode materials. Nanocomposites offer high interfacial areas for fast transfer kinetics between materials of complementary functionalities, such as redox activity and electronic conductivity, as discussed above for metal oxide–carbon nanohybrids. The combination of solid‐state ion conductors with the redox‐active material on the nanoscale would be beneficial for fast and efficient ion diffusion, but is challenging, particularly at high active material content and utilization. These two properties are often opposites, as high active material content decreases the amount of its electrolyte interface and, hence, its accessibility and charge‐transfer kinetics. To address this issue, advanced 3D integrated hybrid architectures on the nanoscale are necessary. Wakayama et al. recently reported on a lithium cobalt oxide (LiCoO_2_, LCO) cathode hybridized on the nanoscale with the solid electrolyte lithium lanthanum zirconium oxide (Li_7_La_3_Zr_2_O_12_, LLZO) from a one‐pot coassembly route with PS‐*b*‐P4VP and inorganic precursors of varying hydrophilicity.^[^ ^160^, ^161^ ^]^ The cobalt precursor selectively incorporated into the spherical P4VP domains of the self‐assembled ordered micellar structure, while the LLZO precursors accumulated in the PS matrix (**Figure** **13**a). The distinct spatial dispersion of the reagents in the BCP morphology enabled the formation of a bicontinuous LCO–LLZO hybrid material upon calcination at 550 °C with domain dimensions of 10s of nanometers.^[^ ^161^ ^]^ To achieve the necessary crystallinity and electrochemical properties of the two inorganic materials, however, calcination at a higher temperature of 750 °C was used, which led to some coarsening of the structure. Among various LCO–LLZO compositions, the lowest impedance was found for 90 wt% LCO with a high utilization of 98% of its theoretical capacity (Figure 13b). The work demonstrated numerous important parameters of advanced solid‐state battery electrode architectures: 1) high interfacial area between solid‐state electrolyte and redox‐active material for fast charge‐transfer kinetics requires nanoscale dimensions of hybridization; 2) bicontinuous morphologies with 3D interconnected electrolyte (LLZO) and conducting material (LCO) enable low electrode impedance; and 3) appropriate crystallinity and low impurity content are necessary, but can cause the destruction of nanoscale integrated morphologies. Further improvements to this system could potentially be made by including a small amount of 3D integrated current collecting material in the nanohybrid and crystal growth inhibitor, such as the homogeneous carbon coating afforded by the CASH method.

## 4 BCPs as Additives and Active Materials in Electrodes

A wide variety of available monomer functionalities and molecular architectures have enabled researchers to synthesize polymers with an exceptional range of properties, including electronic (semi)conductivity, high ion mobility, or even Faradaic redox activity. The combination of polymers with distinct functionalities in the form of a BCP enables the hybridization of these properties into a single material with discrete nanoscale phase dimensions. Batteries require a diverse set of functionalities in their electrodes to function properly: redox activity for charge storage, ionic conductivity for charge balance, and electronic conductivity for charge extraction, as well as structural integrity for longevity. Very few materials combine all these properties in one, and electrodes typically consist of a composite of individual materials executing one of the necessary functions each, such as carbon for conductivity, polymeric binders for mechanical integrity, redox active particles for charge storage, and porosity for liquid or solid electrolyte access. Intimate contact and spatial continuity of these materials and functionalities is crucial because ion and electron extraction/addition start at the redox‐active site and end at the opposite electrode and current collector, respectively. BCPs with intrinsic nanoscale separation of functionalities offer a path toward rational electrode design. In this section, we discuss a number of selected examples demonstrating the capabilities of BCPs as multifunctional materials in battery electrodes, both as ion‐ and electron‐conducting binders and as active redox material.

### 4.1 BCPs as Multifunctional Electrode Additives

Commonly employed composite electrodes contain polymeric binders, such as poly(vinylidene difluoride) (PVDF), to endow the film of powdered active materials and conductive additives with structural stability and prevent disconnection from the current collector. However, the binder adds dead weight to the electrode and thereby lowers the overall energy density. Similarly, a conductive additive and electrolyte is often necessary for proper battery function, but their weight and volume reduce the overall charge storage density. Combining all these functionalities into one material might offer a viable path toward increasing the relative content of charge storing active material in the electrode composite.

**Figure 14:**
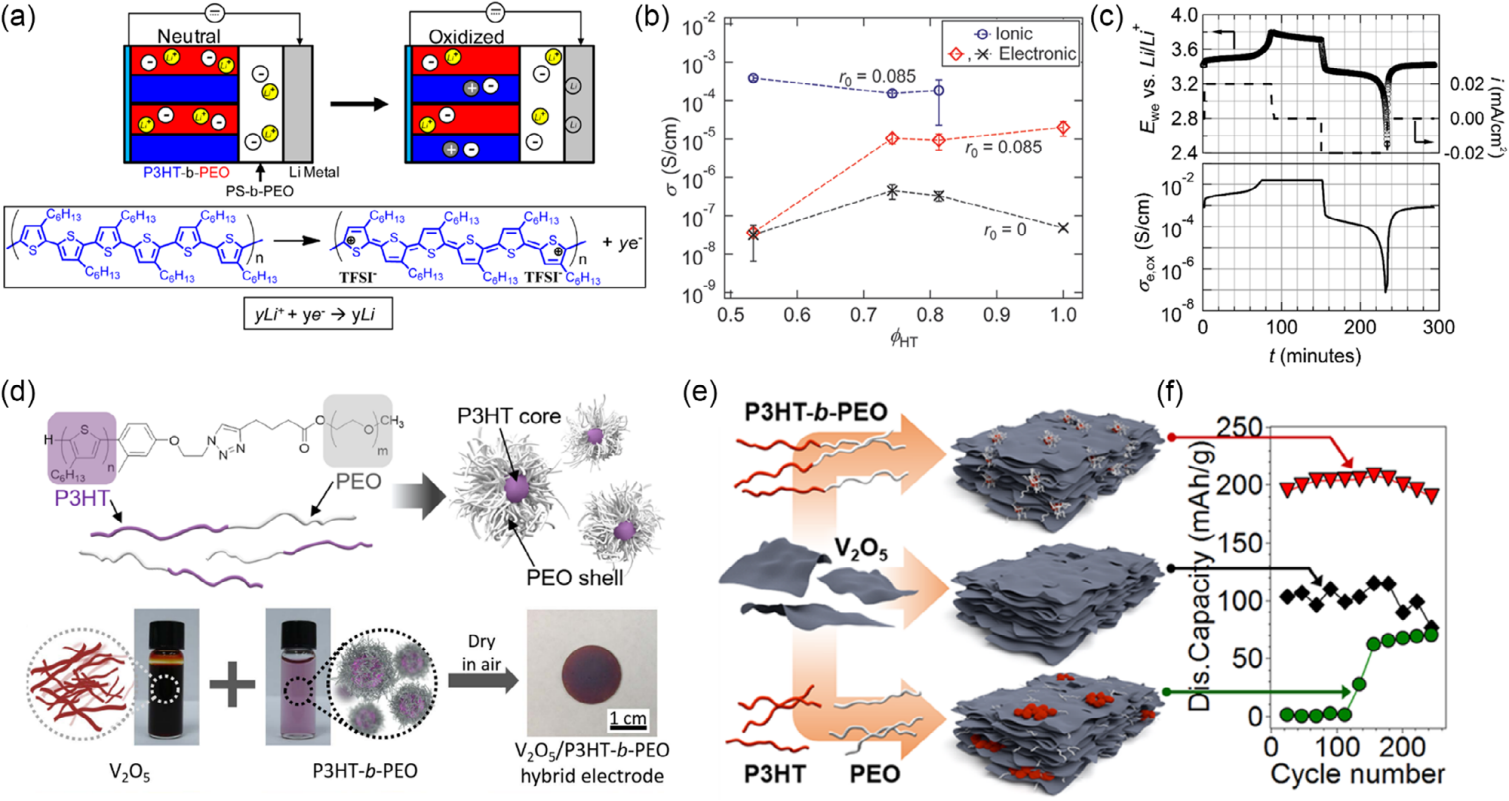
a–c) Nanostructured ion‐ and electron‐conducting P3HT‐*b*‐PEO BCP binder. a) Scheme of a P3HT‐*b*‐PEO electrochemical cell (top) and chemical structure of P3HT (bottom) in its neutral (left) and oxidized (right) state. Adapted with permission.^[^ ^162^ ^]^ Copyright 2013, American Chemical Society. b) Ionic and electronic conductivity of P3HT‐*b*‐PEO polymers as a function of P3HT volume fraction, *Φ*_HT_, at 90 °C for different LiTFSI doping levels (*r*_0_). Adapted with permission.^[^ ^163^ ^]^ Copyright 2012, American Chemical Society. c) Galvanostatic charge/discharge curves showing cell potential versus Li/Li^+^ (top) and electronic conductivity (bottom) of a LiFePO_4_ + P3HT‐*b*‐PEO cathode. Adapted with permission.^[^ ^162^ ^]^ Copyright 2013, American Chemical Society. d–f) Conducting P3HT‐*b*‐PEO BCP micelles as binders for V_2_O_5_ electrodes.^[^ ^165^, ^166^, ^167^ ^]^ d) Schematic (top) and photographs (bottom) of vanadia and P3HT‐*b*‐PEO micellar solutions and dried electrode.^[^ ^166^, ^167^ ^]^ (d) Adapted with permission.^[^ ^166^ ^]^ Copyright 2018, American Chemical Society. e) Scheme of vanadia electrodes with P3HT‐*b*‐PEO BCP (top), no additive (middle), and P3HT + PEO homopolymer (bottom) binders and f) corresponding galvanostatic cycling performance. (e,f) Adapted with permission.^[^ ^165^ ^]^ Copyright 2016, American Chemical Society.

BCPs with one electronically and one ionically conducting block are an intriguing architecture that could satisfy all these necessary functions. Patel et al. studied the properties of the BCP poly(3‐hexylthiopene)‐*block*‐poly(ethylene oxide) (P3HT‐*b*‐PEO) as a multifunctional nanostructured electrode additive, as illustrated in **Figure** **14**a.^[^ ^162^, ^163^ ^]^ The all‐conjugated P3HT is known to exhibit electronic (semi)conductivity when oxidized or doped, while PEO is a well‐studied polymeric lithium‐ion conductor. When doped with lithium bis‐(trifluoromethanesulfonyl)imide (LiTFSI), the ionic conductivity of the BCP was lower than of a PEO homopolymer, typically in the range of 10^−3^ to 10^−4^ S cm^−1^, due to the lower amount of ion‐conducting phase in the BCP. Interestingly, the electronic conductivity of the P3HT‐*b*‐PEO BCPs was an order of magnitude higher than that of a P3HT homopolymer and increased even further upon LiTFSI doping (Figure 14b). Neat P3HT exhibits a low electronic conductivity of 10^−7^ to 10^−8^ S cm^−1^, which increases by orders of magnitude through oxidation and introduction of positive charges along the conjugated polymer chain, balanced by mobile counter ions. The authors concluded that partition of the LiTFSI into the P3HT domain causes the significant increase in electronic conductivity to above 10^−5^ S cm^−1^, which is typically only obtained through oxidative (electro)chemical doping of P3HT.^[^ ^163^ ^]^ When used as the only electrode additive in a solid‐state lithium iron phosphate (LFP) cathode, the multifunctional P3HT‐*b*‐PEO enabled almost theoretical utilization of the LFP capacity.^[^ ^162^, ^164^ ^]^ The electronic conductivity of the P3HT‐*b*‐PEO binder increased even further to 10^−2^ S cm^−1^, due to the oxidative electrochemical potential of LFP. When the battery voltage dropped below 3.2 V versus Li/Li^+^ upon full discharge of the LFP cathode, the electronic conductivity of the P3HT‐*b*‐PEO binder suddenly decreased to 10^−7^ S cm^−1^, due to dedoping of the P3HT domain, as shown in Figure 14c. This is an interesting and potentially useful feature of the investigated system, as it might provide the cathode composite with an internal overdischarge protection by significantly increasing the electronic resistance when full discharge is reached.^[^ ^162^ ^]^ While the amount of P3HT‐*b*‐PEO was high in the system (50 wt%), the study demonstrated the potential of using BCPs as a single multifunctional additive in electrodes, especially for all‐solid‐state batteries.

Similar P3HT‐*b*‐PEO BCPs with varying molar mass and architectures were studied extensively by Lutkenhaus et al. as multifunctional binders in carbon‐free vanadium oxide cathodes.^[^ ^165^, ^166^, ^167^ ^]^ Here, the ionic conductivity was provided by a liquid electrolyte, and the BCP endowed the vanadia xerogel cathode with exceptional structural integrity and flexibility while simultaneously affording the composite with electronic conductivity at only 5–10 wt%.^[^ ^167^ ^]^ A water‐based electrode fabrication method was developed, in which the P3HT‐*b*‐PEO BCP formed micelles and dispersed homogeneously within the vanadia xerogel upon drying, as illustrated in Figure 14d. The benefit of employing BCPs over physical mixtures of the corresponding homopolymers, as well as the importance of regioregularity of the P3HT block for good capacity and reversibility was demonstrated (Figure 14e,f).^[^ ^165^, ^166^ ^]^

### 4.2 BCPs as Charge‐Storing Active Material

**Figure 15:**
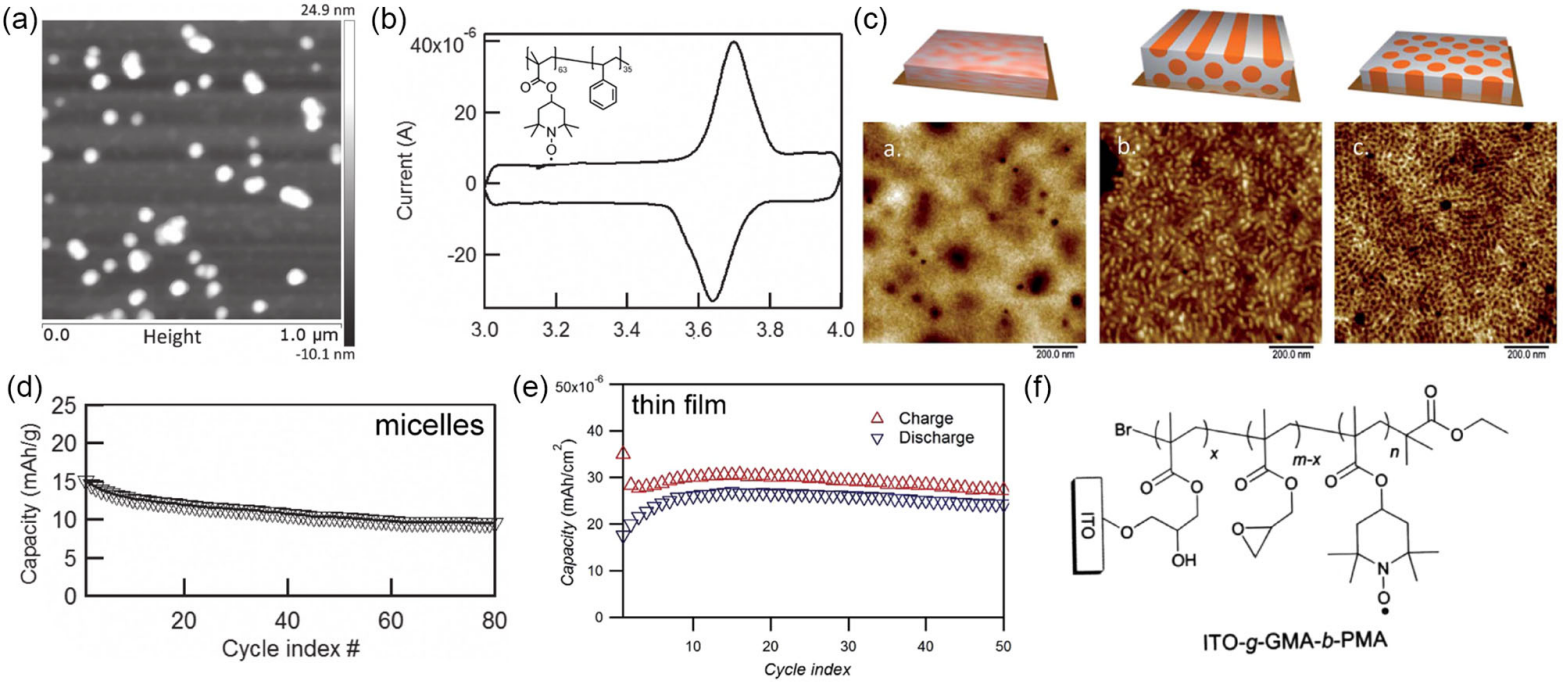
Stable organic radical‐containing BCPs as active electrode materials.^[^ ^168^, ^171^ ^]^ a) AFM height images and b) CV of PTMA‐*b*‐PS BCP micelles. Inset shows the chemical structure of PTMA‐*b*‐PS.^[^ ^168^ ^]^ c) AFM height images of PTMA‐*b*‐PS thin films with 23 wt% PTMA after spin coating (left), and solvent annealed with diethylene carbonate–water (middle) or diethylene carbonate–dimethyl formamide (right).^[^ ^171^ ^]^ d,e) Galvanostatic cycling performance of PTMA‐*b*‐PS micellar (d) and thin film (e) electrodes.^[^ ^168^, ^171^ ^]^ f) Chemical structure of PTMA‐*b*‐PGMA covalently cross‐linked to ITO current collectors enabling structurally stable thick films of organic radical‐containing BCPs as active electrode material.^[^ ^172^ ^]^ (a,b,d) Adapted with permission.^[^ ^168^ ^]^ Copyright 2014, Wiley‐VCH. (c,e) Adapted with permission.^[^ ^171^ ^]^ Copyright 2015, Royal Society of Chemistry. (f) Reproduced with permission.^[^ ^172^ ^]^ Copyright 2015, Elsevier.

Charge storage in polymeric materials could enable lightweight electrodes with molecular tunability and facile processability. Stable organic radicals exhibit highly reversible and fast electrochemical response with decent gravimetric capacity. The most studied organic radical unit in polymeric systems is 2,2,6,6‐tetramethyl‐1‐piperidinyloxy (TEMPO, or simply T) in the form of a poly(methacrylate) (PMA), i.e., PTMA. PTMA has a theoretical capacity of 111 mAh g^−1^ at 3.6 V versus Li/Li^+^, making it a viable candidate for all‐organic, metal‐free lithium‐ion cathodes. However, the solubility of PTMA in common organic electrolytes such as carbonates causes active material loss and capacity fading. To overcome these issues, BCPs of PTMA and poly(styrene) (PS) have been studied recently as active cathode materials in the form of micelles,^[^ ^168^, ^169^, ^170^ ^]^ and self‐assembled thin films (**Figure** **15**).^[^ ^171^ ^]^ The addition of an insoluble minority PS block to PTMA leads to the formation of PTMA‐*b*‐PS micelles with diameters of 30–40 nm in carbonate electrolytes (Figure 15a).^[^ ^169^ ^]^ When infiltrated into carbon nanotubes buckypaper, 65% of the theoretical PTMA capacity could be accessed. A capacity fade of 50% over 80 cycles was observed though, due to the nonpermanent attachment to the carbon nanotubes and structural instability of the micelles (Figure 15d).^[^ ^168^ ^]^ The structural integrity of the PS matrix prevented the dissolution and capacity fade in ordered self‐assembled thin films of PS‐*b*‐PTMA BCPs with hexagonally packed PTMA cylinders or lamellar morphology (Figure 15e).^[^ ^171^ ^]^ However, low areal capacity and high self‐discharge rates were observed, a common problem in thin film electrodes. To overcome this issue, redox‐active films of up to 5 μm thickness could be obtained from a PTMA‐*block*‐poly(glycidyl methacrylate) (PTMA‐*b*‐PGMA) BCP.^[^ ^172^ ^]^ The epoxy groups of the glycidyl methacrylate block were covalently attached to a conductive oxide substrate. The chemical attachment and cross‐linking within the BCP film endowed it with structural stability toward dissolution (Figure 15f).

**Figure 16:**
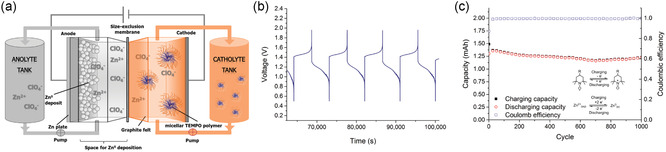
PTMA‐*b*‐PS/zinc hybrid‐flow battery (pHFB). a) Scheme of pHFB with zinc perchlorate as anolyte and electrolyte salt, and PTMA‐*b*‐PS micelles as active catholyte solute. b) Galvanostatic charge–discharge curves of a pHFB at 1 mA cm^−2^ under flow conditions and c) galvanostatic cycling performance of a pHFB at 1.5 mA cm^−2^ under static conditions. The inset in (c) shows Faradaic reactions of the active catholyte (top) and anolyte (bottom) solute. (a,b) Adapted with permission.^[^ ^170^ ^]^ Copyright 2016, Royal Society of Chemistry.

Instead of immobilized solid redox‐active materials, flow batteries take advantage of, and even require, dissolved Faradaic species. PTMA that is dissolved in carbonates functions as a catholyte in redox‐flow batteries, but for high energy density of the catholyte a high PTMA concentration is necessary, leading to an unsustainably high viscosity. Micelles of PTMA‐*b*‐PS BCPs exhibit significantly lower viscosity. A catholyte with PTMA‐*b*‐PS micelles containing the redox‐active TEMPO unit in the corona was successfully applied in zinc hybrid‐flow batteries with good efficiency and only 5% capacity loss over 2000 cycles at a stable potential of 2 V, as shown in **Figure** **16**.^[^ ^173^ ^]^

## 5 Next‐Generation Hierarchical and Multiscale Architectures

### 5.1 Beyond Equilibrium for Hierarchically Structured BCP‐Derived Materials

Materials with tunable porosity on multiple length scales offer fast mass transfer kinetics with control over diffusion pathways. EES materials and electrodes can benefit from such hierarchical porosity featuring macro‐ (>50 nm) and mesopores (2–50 nm), for example, by providing good electrolyte access and fast diffusion times over 10–100s of micrometers through the macropores while at the same time exhibiting high surface area due to the presence of mesopores.^[^ ^83^ ^]^ To achieve hierarchically structured and porous materials, the synthesis of ordered mesoporous functional materials such as metal oxides and carbons from BCP self‐assembly can be simply combined with traditional hard‐templating methods for micrometer scale feature sizes.^[^ ^174^ ^]^ An example of this confined templating method is the self‐assembly of BCPs in closed‐packed films of micrometer‐sized colloids, so‐called inverse opal templating.^[^ ^173^ ^]^ However, the EISA and structure direction of BCPs can be combined with virtually any molding or structuring technique, such as photolithography, controlled crystallization, electrospinning, and casting inside other nonspherical porous templates such as anodized alumina membranes.^[^ ^175^, ^176^, ^177^, ^178^, ^179^ ^]^ An impressive proof‐of‐concept demonstration of a functional hierarchical material featuring all length scales from millimeters to nanometers employed a combination of soft‐lithography, multiscale inverse opal templating, and BCP structure direction.^[^ ^180^ ^]^ The final high‐temperature ceramic decorated with platinum nanoparticles exhibited eight levels of structural length scales imposed on the material by the synthesis method. However, for many EES applications, hierarchically structured materials and architectures should be accessible as bulk materials with dimensions of at least 10–100s of micrometers in thickness, which precludes many of the thin‐film techniques mentioned above. Additionally, tailored and tunable porosity in the mesopore and macropore regime is necessary to obtain a good balance between material loading (energy density) and mass transport such as electrolyte access for fast ion diffusion (power density).

Hard templates offer exquisite control over structural features and porosity on multiple length scales, but the removal of inorganic templating materials is tedious, often involving hazardous chemicals, and the film thickness is often limited to below 10 μm. One‐step structuring methods combining BCP self‐assembly and other soft‐matter phase separation mechanisms represent a facile alternative to tedious multistep combinations of hard‐ and soft‐templating methods and may increase the accessible film thickness to bulk dimensions. Dual‐phase separation of BCPs and solvents to yield macroporous networks of ordered mesoporous silicas or carbons were achieved over a decade ago using water‐ and alcohol‐soluble small BCPs such as the commercially available Pluronics.^[^ ^181^, ^182^, ^183^, ^184^ ^]^ However, the small polymer sizes applicable in these systems limited the mesopore size to below 10 nm. Multiscale phase separation methods with large mesopores from high molar mass BCPs were described only recently.^[^ ^34^, ^185^, ^186^, ^187^ ^]^ Two dual‐phase separation methods to create hierarchically porous materials that move away from simple equilibrium considerations of structure formation have gained increasing interest over the last years: BCP self‐assembly combined with spinodal decomposition, as well as nonsolvent‐induced phase separation (NIPS). In this section, we briefly describe both methods and point toward their potential usefulness to produce structures for novel EES materials and architectures.

#### 5.1.1 Spinodal Decomposition with BCP Microphase Separation

**Figure 17:**
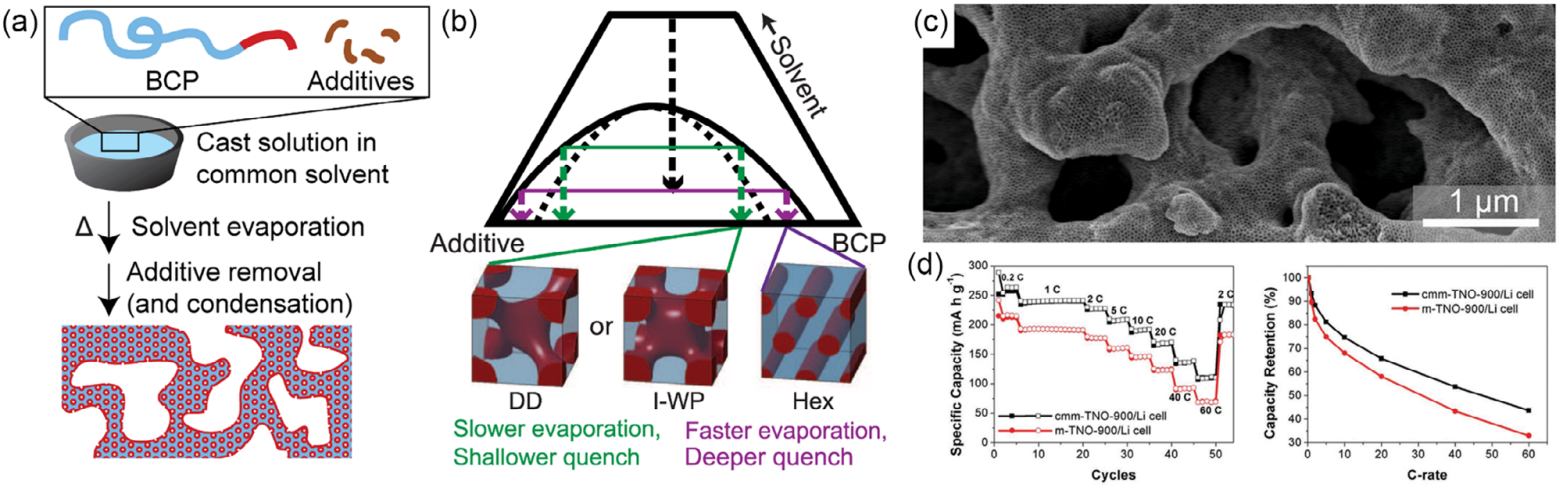
a) Synthesis pathway for hierarchically porous polymer scaffolds with ordered mesostructure through combined spinodal decomposition and BCP microphase separation.^[^ ^34^ ^]^ b) Schematic ternary phase diagram illustrating the formation pathway by solvent evaporation and the influence of quench depth on phase composition. (a,b) Adapted with permission.^[^ ^34^ ^]^ Copyright 2013, American Association for the Advancement of Science. c) SEM image of cocontinuous meso/macroporous titanium‐niobium oxide (cmm‐TNO) heat treated at 500 °C.^[^ ^158^ ^]^ d) Galvanostatic cycling and rate behavior of cmm‐TNO compared to mesoporous TNO as monolithic electrodes in LIBs.^[^ ^158^ ^]^ (c,d) Adapted with permission.^[^ ^158^ ^]^ Copyright 2017, Wiley‐VCH.

Macrophase separation of polymer blends or solvent–polymer mixtures causes a demixing of the constituents on length scales above 100 nm and depends on the composition and phase separation pathway. Slow or shallow transitions from miscible to immiscible conditions of mixtures lead to the nucleation and growth of an often spherical minority phase in a matrix of the majority phase. Spinodal decomposition is obtained when a homogeneous mixture is quenched beyond the thermodynamic stability limit and often results in bicontinuous morphologies with characteristic dimensions of 100s of nanometers to micrometers. Spinodal decomposition can be induced by changes in temperature, solvent conditions such as selective or full solvent removal, as well as chemical changes through polymerization or cross‐linking. Recently, spinodal decomposition has been successfully combined with BCP phase separation through solvent evaporation from mixtures of large molar mass PS‐*b*‐PEO and oligo(ethylene oxide), creating hierarchal porous polymer scaffolds with a bicontinuous macroporous architecture and ordered mesoporous cylinders or ordered cocontinuous cubic networks within its micrometer‐sized struts, as illustrated in **Figure** **17**a.^[^ ^34^ ^]^ The nanostructure from BCP self‐assembly was controlled by the evaporation temperature, which relates to the evaporation rate and hence the quenching depth of the spinodal decomposition, endowing the method with a unique handle over multiple structural parameters at once (Figure 17b). Such hierarchically porous and bicontinuous architectures are promising for use in energy storage devices due to their monolithic nature, eliminating the need for structural additives such as binders, and the facile access to the large surface area of the mesoporous walls through the macropore network.

Indeed, the structure direction of BCPs to create mesoporous inorganic materials was very recently combined with spinodal decomposition to form hierarchically porous metal oxides of interest for high‐rate electrodes in LIBs.^[^ ^157^, ^158^, ^186^ ^]^ In their first report, Lee et al. employed the chemical cross‐linking of phenol–formaldehyde resols to induce spinodal decomposition and phase separation from a PS‐*b*‐PEO silica/titania mixture.^[^ ^186^ ^]^ The hierarchical meso/macroporous titania comprised a 3D network with strut and pore sizes of 100s of nm and ordered mesoporous hexagonally packed cylinders within the crystalline titania struts. Due to the additional macropores, the titania showed significantly improved rate capability as a lithium‐ion anode as compared to a purely mesoporous analogue with 113 mAh g^−1^ at a rate of 2 °C, demonstrating the potential of hierarchical architectures for high‐rate battery applications.^[^ ^186^ ^]^ Unfortunately, through the use of cross‐linking resols to induce spinodal decomposition, calcination in air was necessary to obtain macroporosity, precluding the incorporation of carbon coatings or nanowiring as obtained with the CASH method. To overcome this issue, in their latest iteration of multiscale phase separations for hierarchically ordered macro/mesostructured metal oxides, Lee et al. employed nitric acid as macrophase separation‐inducing porogen.^[^ ^157^, ^158^ ^]^ From a homogenous solution of nitric acid, PS‐*b*‐PEO, and inorganic sol nanoparticles in tetrahydrofuran (THF), evaporation of the THF leads to spinodal decomposition of the nitric acid from a microphase separating PS‐*b*‐PEO‐inorganic hybrid domain, as shown in Figure 17c. The macrostructure of the material is tunable from mesoporous microparticles to monoliths of bicontinuous mesoporous networks and mesoporous bulk material containing spherical voids by decreasing the amount of nitric acid.^[^ ^158^ ^]^ The method is highly versatile, allowing for the incorporation of carbon precursors and multiple transition metal oxides. The advantage of hierarchical porosity is again demonstrated by the superior high‐rate capacity of macro/mesoporous titanium‐niobium oxides over purely mesoporous monolithic electrodes (Figure 17d), as reported for other materials described in the previous sections. Spinodal decomposition of incompatible homopolymers with BCP‐inorganic self‐assembly has also recently been demonstrated to produce ordered mesoporous microparticles and ‐capsules of a variety of inorganic materials of interest for EES.^[^ ^118^ ^]^

While higher porosity essentially always increases the rate capabilities of electrode materials, it also decreases the amount of active material per volume and footprint area on the device scale. Hence, excessive and unnecessary porosity has to be avoided especially for portable and transportation‐related energy storage applications. Combined BCP self‐assembly and spinodal decomposition represents not only a facile one‐step structuring method for hierarchically porous electrode materials, but it also allows for precise tuning and balancing of these architectural parameters to optimally match the requirements of a given EES application. More research is necessary, however, to understand the exact requirements for different types of energy storage devices and truly make use of the full potential of these intriguing architectures for full‐scale devices or even novel and revolutionary interdigitated energy storage architectures as described below.

#### 5.1.2 BCP Self‐Assembly and Nonsolvent‐Induced Phase Separation

**Figure 18:**
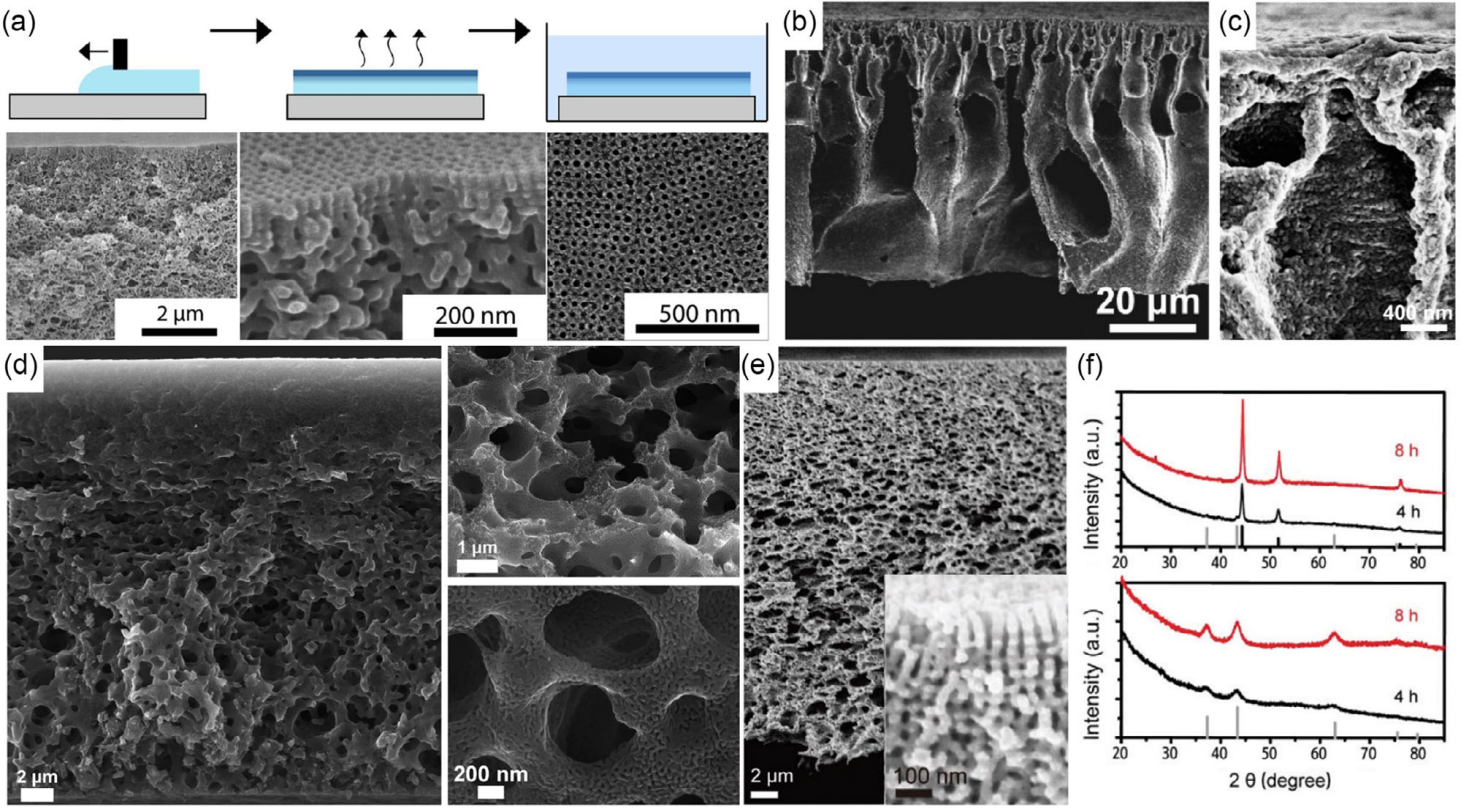
a) Schematic of the SNIPS process (top) to fabricate hierarchically porous BCP membranes with graded porosity along the film normal (SEM bottom left), and a periodically ordered mesoporous separation layer on the top surface (SEM bottom middle and right). Adapted with permission.^[^ ^143^ ^]^ Copyright 2011, American Chemical Society. b,c) Cross‐sectional SEM images of BCP–titania hybrid membranes exhibiting graded macroporosity (b) and mesoporous wall structure (c). b,c) Adapted with permission.^[^ ^185^ ^]^ Copyright 2013, American Chemical Society. d,e) Cross‐sectional SEM images of graded and hierarchically macro/mesoporous carbon (d) and nickel (e) membranes hard‐templated from BCP SNIPS membranes. Inset in (e) shows the ordered mesoporous top layer at higher magnification.^[^ ^191^ ^]^ f) XRD traces of nickel and nickel oxide membranes heat treated at 600 °C in nitrogen (top) and air (bottom), respectively, after electroless deposition of nickel onto ISV SNIPS membranes for indicated times. (d,e) Adapted with permission.^[^ ^191^ ^]^ Copyright 2015, Royal Society of Chemistry.

Phase inversion membranes are very common in filtration and separation applications and are manufactured on the industrial scale.^[^ ^188^ ^]^ Among the various phase inversion processes, nonsolvent‐induced phase separation (NIPS), or also called immersion precipitation, is the most widely applied method. In the NIPS process, a polymer solution is cast on a substrate and after partial evaporation of the solvent(s) is subsequently immersed into a coagulation bath containing a nonsolvent for the polymer that is miscible with the solvent(s) of the original polymer solution. Upon immersion in the coagulation bath, the exchange between solvent and nonsolvent in the casted film causes the polymer to precipitate and solidify. The structure of the asymmetric porous polymer membrane is affected by the mass‐transfer and phase separation kinetics, and typically displays characteristic pore dimensions of 100s of nanometers to 10s of micrometers. From the partial solvent evaporation in the time between casting and precipitation of the polymer solution, a gradient of polymer concentration with its maximum toward the air–solution interface leads to an asymmetric porosity along the cross section of the phase inversion membrane. The pore size gradually increases from the initial air–interface of the film toward the other side, yielding a thin separation layer on top of a macroporous substructure for high flux filtration applications. The introduction of BCPs as phase‐inversion membranes afforded the thin separation layer with periodically ordered and highly homogeneous nanopores, as first demonstrated by Peinemann et al. using diblock copolymer PS‐*b*‐P4VP.^[^ ^144^ ^]^ The method termed self‐assembly and nonsolvent‐induced phase separation (SNIPS)^[^ ^189^ ^]^ has since been applied to many more BCP‐solvent systems, including the use of the triblock terpolymer poly(isoprene)‐*block*‐poly(styrene)‐*block*‐poly(4‐vinylpyridene) (PI‐*b*‐PS‐*b*‐P4VP, or ISV).^[^ ^143^, ^187^, ^189^ ^]^ The self‐assembly of spherical or cylindrical BCP micelles at the solution–air interface during a distinct solvent‐evaporation period before immersion in the coagulation bath causes the formation of almost monodisperse nanopores at ultrahigh pore density arranged in periodic lattices in the thin separation layer on top of the asymmetric meso‐ to macroporous membrane, as shown in **Figure** **18**a, combining high resolution with high flux in separation applications.

While the fabricated asymmetric BCP membranes demonstrate great potential for ultrafiltration and separation applications, their limited chemical functionality renders them less useful for active energy storage materials, except as porous separators.^[^ ^190^ ^]^ However, numerous structural features of asymmetric SNIPS membranes are very intriguing for electrode films if they could be transferred to electrochemically functional materials: 1) The walls in the macroporous substructure of SNIPS membranes can be rendered mesoporous from the BCP microphase separation, yielding an asymmetric/hierarchically porous macro/mesoporous architecture with well‐defined mesopores throughout the material potentially yielding a combination of high surface area and fast diffusion kinetics. 2) The architecture is inherently asymmetric with increasing macropore size along the film normal, a feature that could also be beneficial for the inherently asymmetric design of devices such as batteries, supercapacitors, and fuel cells. Traditional device designs exhibit 2D layered films where the energy‐storing electrode composite interfaces the current collector on one side and the separator/electrolyte on the other. Especially for thicker electrode films that are desirable to increase device‐scale energy densities, a gradient in properties such as electronic and ionic conductivity, as well as the amount of electrochemically active material, is highly desirable to most efficiently utilize charge storage capacities and electrode volume. For example, higher electronic conductivity is necessary toward the current collector side of an electrode that is accomplished by the presence of more conductive material, while higher ionic conductivity is beneficial on the separator side of the electrode film, which can be achieved through higher porosity. Asymmetrically porous membrane architectures through the SNIPS process are ideal candidates to test and apply such advanced electrode structures.

To increase the range of accessible materials and functionalities of asymmetric or graded hierarchical macro/mesoporous films, the Wiesner group pioneered a number of soft‐ and hard‐templating methods for inorganic materials using the SNIPS process.^[^ ^185^, ^191^, ^192^ ^]^ In a first step, the addition of titania sol nanoparticle precursors to the ISV BCP solution and subsequent SNIPS membrane formation yielded organic–inorganic hybrid membranes with titania contents up to 15 wt% in a one‐step fabrication process.^[^ ^185^ ^]^ The hierarchical macro/mesoporous hybrid membranes exhibited a so‐called finger‐like structure with cylindrical macropores of increasing diameter from 200 nm to 20 micrometers along the film normal, as shown in Figure 18b,c. The walls of the cylindrical macropores exhibited a homogeneous and accessible mesoporous substructure. In a similar “one‐pot” approach, through the addition of phenol–formaldehyde resols to an ISV casting solution, graded and hierarchically macro/mesoporous carbon materials were formed after pyrolysis.^[^ ^192^ ^]^ The abundant hydroxyl groups of the resols enabled their homogeneous and sufficient incorporation into the 4‐vinylpyridine domains of the SNIPS membrane through hydrogen bonding. Upon heat treatment at 1100 °C in inert atmosphere, the BCP decomposed and the resols carbonized without loss of the graded and hierarchical superstructure. The one‐pot method yielded monolithic carbon membranes with mesopores throughout the asymmetric structure, characterized by a broad mesopore size distribution around 20 nm from the BCP self‐assembly and graded macropores on the micrometer scale from the phase inversion process.

**Figure 19:**
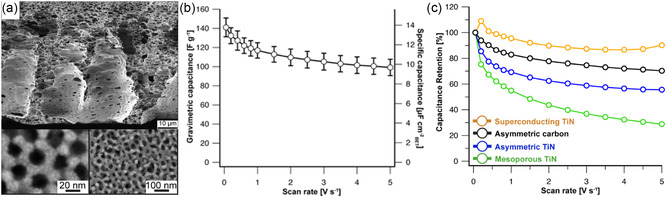
Asymmetric meso‐ and macroporous SNIPS‐derived supercapacitor electrodes: a) SEM images of a SNIPS‐derived asymmetric titanium nitride (TiN) electrode (top: cross section) with mesoporous top surface (bottom left) and mesoporous walls (bottom right). b) Specific and surface‐area‐normalized capacitance of SNIPS‐derived asymmetric carbon electrodes at CV scan rates up to 5 V s^−1^. c) Capacitance retention of SNIPS‐derived asymmetric carbon and titanium nitride electrodes and a titanium nitride electrode with only mesoporosity at CV scan rates up to 5 V s^−1^ relative to the capacitance at 50 mV s^−1^. (a–c) Adapted with permission.^[^ ^194^ ^]^ Copyright 2020, American Chemical Society.

The variety of accessible inorganic materials was further extended to metals and metal oxides, i.e., nickel, copper, and nickel oxide (NiO) by using polymeric ISV SNIPS membranes as hard templates to deposit the functional material of choice.^[^ ^191^ ^]^ Interestingly, both electroless plating of metals and adsorption of carbon‐forming resols onto ISV SNIPS membranes replicated the hierarchical graded structure in great detail and without filling the macropores with excess deposited material, as shown in Figure 18d–f. The metal‐complexing and hydrogen bond‐accepting properties of the 4‐vinylpyridine block play an important role in the deposition process because the architecture is only stable during thermal BCP removal if the functional material is homogeneously dispersed and percolated throughout the mesostructured part of the membrane. These metal, metal oxide, and carbon membranes with asymmetric and hierarchical porosity, as well as mesopores throughout the material, are promising as advanced monolithic electrode architectures, as suggested by supercapacitor electrodes using carbon‐forming PAN in a homopolymer–triblock copolymer mixture subjected to the NIPS process.^[^ ^193^ ^]^ Ongoing research in the Wiesner group on SNIPS‐derived asymmetric hierarchical membrane‐type structures further demonstrates the possibilities of such architectures as EES electrodes.^[^ ^194^, ^195^, ^196^, ^197^ ^]^ Carbon and (super)conducting titanium nitride (TiN) membranes were derived from SNIPS with ISV BCPs and tested as EDLC electrodes with 0.1 m aqueous HClO_4_ (**Figure** **19**).^[^ ^194^ ^]^ The asymmetric carbon electrodes displayed a specific capacitance of almost 100 F g^−1^ at an ultrahigh sweep rate of 5 V s^−1^, a 70% retention of its 50 mV s^−1^ capacitance, as shown in Figure 19b. The superconducting asymmetric TiN electrodes exhibited a capacitance retention of almost 90% of its 50 mV s^−1^ capacitance at sweep rates of up to 5 V s^−1^, which is impressive especially when considering that the electrode is monolithic with a thickness of around 50 μm (Figure 19a,c). High surface area nickel substrates obtained from electroless deposition of nickel on ISV SNIPS membranes have been electrochemically converted to Prussian blue analogues that demonstrated remarkable rate capability for reversibly intercalating sodium ions at rates up to 1667 C.^[^ ^195^ ^]^

### 5.2 3D Interpenetrating Device Architectures from BCP Self‐Assembly

The quest for EES systems that provide high power and energy density at efficient use of volume and footprint area requires rethinking of device architectures beyond the optimization of individual components. The nanostructuring of active materials and the introduction of vast amounts of porosity allowed for ultrahigh rate capabilities in battery electrodes combined with attractive gravimetric energy densities, suggesting a viable path toward devices with battery‐like energy densities at capacitor‐like power capabilities. However, the high porosity of many systems not only yields low volumetric and areal energy densities, but, when considering the weight of the electrolyte that necessarily fills the pores, also translates to massively decreased gravimetric energy density on the full device scale. An intriguing concept to leverage the benefits of nanostructuring and short solid‐state ion‐diffusion lengths without wasting space and mass on excess electrolyte was introduced over 15 years ago and termed 3D batteries.^[^ ^198^ ^]^ In 3D battery architectures, the space within the micro/nanostructure of one electrode is filled with the other components of the battery, namely, an electrolyte separation layer and the second electrode.^[^ ^198^, ^199^, ^200^ ^]^ Conceptually, the energy density (volumetric, gravimetric) is determined by the amount of active materials in the integrated and 3D cocontinuous interpenetrating architecture. The power density, mostly determined by solid‐state ion‐diffusion distances, depends on the characteristic structural length scale, thickness, and separation of the interpenetrating electrodes and electrolyte. If these architectural parameters are controlled independently, the energy and power density in such systems are essentially decoupled. The difficulty of synthesizing multifunctional, multimaterial device architectures with the necessary spatial precision on the nanoscale over macroscopic dimension has hindered the exploration of the full potential of the (nano‐) 3D battery concept.^[^ ^201^, ^202^ ^]^

BCP self‐assembled morphologies offer intriguing design concepts for 3D integrated and interpenetrating multicomponent architectures. Multi‐BCPs such as ABC triblock terpolymers display a variety of morphologies and defined interfaces between multiple (3+) distinct phases that are intriguing for adaptation as 3D battery architectures. Key requirements for 3D batteries are the connectivity of and separation between the two electrode phases throughout the entire architecture for charge extraction and short‐circuit prevention, respectively. The requirements are met in tricontinuous nonfrustrated ABC triblock terpolymer morphologies with anode, electrolyte, and cathode incorporated in the A, B, and C domains, respectively. The A and C domains in nonfrustrated triblock terpolymers share no interface but are completely separated by the B middle block due to the favorable AB and BC and unfavorable AC interfacial energies, ensuring complete separation between the electrodes if adapted as a battery architecture. Thermodynamically stable morphologies such as the core–shell double gyroid, the alternating gyroid, or the orthorhombic O^[^ ^69^ ^]^ phase additionally meet the requirement of cocontinuity of all domains, rendering them prime candidates for the spatially precise formation of 3D batteries with characteristic component dimensions on the nanoscale through self‐assembly, representing a scalable bottom‐up approach for nano‐3D batteries that is not limited to thin films.

**Figure 20:**
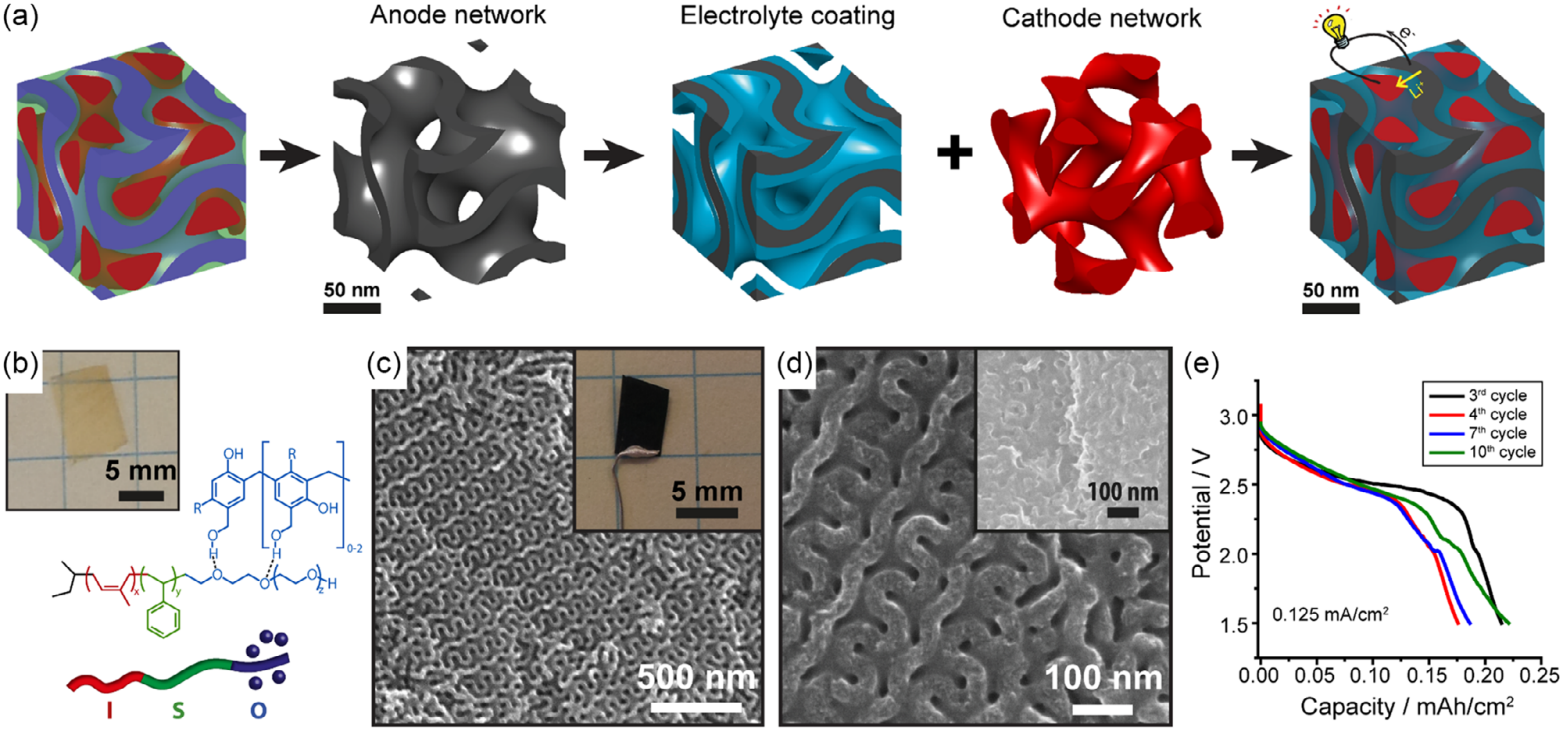
a) Schematic illustration of the bottom‐up step‐by‐step synthesis of a BCP self‐assembly‐derived gyroidal 3D nanointerpenetrating battery with b–e) corresponding characterization data.^[^ ^204^ ^]^ b) Chemical structure of the ISO BCP and resols carbon precursor, inset: photograph of the polymer‐organic hybrid. c) SEM image of the double gyroidal mesoporous carbon (G^D^MC) anode framework after pyrolysis of the hybrid shown in (b). Inset shows photograph of electrically wire‐connected G^D^MC anode monolith. d) SEM image of the G^D^MC anode coated with PPO solid electrolyte (main) and infiltrated with sulfur–PEDOT cathode composite (inset), finalizing the core–shell double‐gyroid 3D battery assembly. e) Selected galvanostatic discharge traces showing the reversibility of the first sulfur discharge plateau in the gyroidal nanointegrated 3D battery. (a–e) Adapted with permission.^[^ ^204^ ^]^ Copyright 2018, Royal Society of Chemistry.

The direct synthesis and self‐assembly of triblock terpolymers with redox‐active and electronically conductive A and C blocks of significantly different electrochemical potentials, and ionically conductive but electronically insulating B block has not yet yielded functioning 3D batteries.^[^ ^203^ ^]^ In an alternative pathway, Werner et al. recently reported a triblock terpolymer self‐assembly‐derived bottom‐up step‐by‐step approach to create a core–shell double‐gyroid electrochemical cell with battery‐like charge/discharge properties and characteristic thickness of all components below 20 nm, as illustrated in **Figure** **20**a.^[^ ^204^ ^]^ First, the coassembly of a large molar mass PI‐*b*‐PS‐*b*‐PEO (ISO) with phenol–formaldehyde resols yielded monolithic polymer–organic hybrids with core–shell double‐gyroid morphology, consisting of a poly(ethylene oxide)–resols matrix separated from two interpenetrating 3D poly(isoprene) core and poly(styrene) shell (core–shell) minority networks (Figure 20b). The hybrid films were converted to double gyroidal mesoporous carbon (G^D^MC) monoliths with ultralarge mesopore size of 40 nm and fully accessible mesoporosity over the macroscopic film dimensions of around 4 × 4 mm^2^ at 70 μm thickness (Figure 20c). These monoliths simultaneously served as the structural framework, active anode material, and anode current collector. The large pore size enabled the successive conformal electrodeposition of a 10 nm poly(phenylene oxide) (PPO) pinhole‐free polymer electrolyte layer followed by infiltration with the redox‐active and conductive cathode composite of sulfur and poly(3,4‐ethylenedioxythiophene) (PEDOT) at a high filling fraction, as shown in Figure 20d.

The final core–shell double gyroidal 3D nanointerpenetrating battery consisted of a gyroidal carbon majority matrix separated from two interpenetrating sulfur–PEDOT minority networks by a PPO shell, resembling the starting polymer–organic morphology. The gyroidal carbon anode and sulfur–PEDOT cathode could be electrically contacted independently and, subsequent to sulfur lithiation and reduction, the gyroidal 3D battery was galvanostatically charged and discharged over 20 cycles. A monolithic gyroidal 3D battery of around 70 μm thickness exhibited a maximum areal capacity of 1.3 mAh cm^−2^ and a reversible capacity of 0.2 mAh cm^−2^, corresponding to only the first discharge plateau of sulfur at 2.5 V (Figure 20e). The second discharge plateau at 2 V proved to be irreversible due to the insulating behavior of the nanointegrated PEDOT current collector at lower potentials. While only 20% of the 3D battery capacity was accessible upon cycling, the work clearly demonstrated the possibility of achieving EES in nanoscaled interpenetrating architectures at overall bulk device dimensions.

The gyroidal 3D battery architecture described above resembled a BCP equilibrium morphology with roughly equal volume fractions of anode, cathode, and electrolyte. In battery architectures, the device‐scale energy density is increased through the use of higher capacity charge‐storing electrode materials, or a decrease of the fraction of noncharge‐storing components such as the electrolyte layer. Redox active anode and cathode materials with higher gravimetric or volumetric capacity typically exhibit large volume changes upon charge and discharge, which could cause structural disruption of the interpenetrating 3D battery architectures during operation. Hence, we believe that a more viable path for high‐energy 3D batteries lies in the development of architectures with small electrolyte (10–50 nm) and larger electrode dimensions (50–1000 nm). In fact, control over these parameters could enable 3D batteries tailored to the specific power and energy requirements of a given application. Hierarchical structures with features on the nano‐ and microscale as described in the previous section are very promising candidates to achieve desirable 3D architectures for next‐generation EES.^[^ ^187^ ^]^ The asymmetric morphologies afforded by phase inversion methods are particularly interesting, as they provide a pathway toward rational 3D battery architectures with a gradient of electrode domain, increasing in fraction toward the respective external contact to accommodate the nonsymmetric current density distribution.^[^ ^191^, ^192^, ^205^ ^]^ A key challenge for the realization of such high‐performing 3D batteries remains the thin solid electrolyte/separator layer between the interdigitated anode and cathode. As thin electrolyte layers will lead to high energy densities, but defects cause short circuits and heterogeneities lead to overactive hot‐spots, novel fabrication methods for conformal and uniform solid electrolyte coatings and interphases need to be developed.

## 6 Conclusions

We have provided an overview of the use of BCP self‐assembly as a tool to generate components as well as entire device concepts in the area of EES. The exquisite structure control that is afforded by this approach allows for systematic studies of structural and compositional effects on EES devices that are difficult to achieve otherwise. In turn, this enables to separate out different contribution to the observed behavior of such devices, which is required to generate a deeper fundamental understanding of the often complex interplay of different factors determining their performance. One of the fascinating features of this approach is that it extends over a wide spectrum of organic, inorganic, as well as organic–inorganic hybrid material classes. From the highlighted work it is evident that substantial progress has been made in this field over the last one to two decades. While significant challenges remain to be overcome, in particular work described in the last section illustrates that there continue to be substantial opportunities for research and development of BCP self‐assembly based EES materials and devices.

It is evident from the work highlighted in this review that the primary dimensions of both material and pore phases have significant impact on the utility and performance of EES materials and architectures. It is well established that increasing porosity, surface area, and pore size exhibit a positive effect on rate capabilities due to increased mass transfer rates and charge transfer kinetics. Our discussion of well‐defined BCP‐derived materials provided here demonstrates, however, that the pore space cannot simply be defined by only those two metrics. The detailed architecture, connectivity, accessibility, tortuosity, and geometric pore size distribution are highly relevant as well, as the fidelity and control of BCP self‐assembly has enabled to elucidate. We anticipate that such structure control will have a similar impact of revealing fundamental structure–property–performance relationships in next‐generation multiscale architected components and devices for EES. As we have highlighted in the last section, future advances likely require further developments in combining equilibrium BCP self‐assembly with complementary nonequilibrium processes to access hierarchical architectures with controlled structural features from the nanometer to the millimeter scale. Such novel material designs will pose novel challenges, including the control over anisotropy, gradation, tortuosity and content of the macropores, as well as their balance with and connectivity to the BCP‐derived mesopore phase. We anticipate that this will enable to establish scale‐bridging correlations and provide new fundamental insights into application‐relevant cell designs for next‐generation advanced EES.

## Conflict of Interest

J.G.W. and U.W. have filed patents on some of the technologies described in this review.
